# Il-10 signaling reduces survival in mouse models of synucleinopathy

**DOI:** 10.1038/s41531-021-00169-8

**Published:** 2021-03-19

**Authors:** Samuel G. Cockey, Karen N. McFarland, Emily J. Koller, Mieu M. T. Brooks, Elsa Gonzalez De La Cruz, Pedro E. Cruz, Carolina Ceballos-Diaz, Awilda M. Rosario, Yona R. Levites, David R. Borchelt, Todd E. Golde, Benoit I. Giasson, Paramita Chakrabarty

**Affiliations:** 1 Center for Translational Research in Neurodegenerative Disease, University of Florida, Gainesville, FL USA; 2 Norman Fixel Institute for Neurological Diseases, University of Florida, Gainesville, FL USA; 3 Department of Neurology, University of Florida, Gainesville, FL USA; 4 McKnight Brain Institute, University of Florida, Gainesville, FL USA; 5 Department of Neuroscience, University of Florida, Gainesville, FL USA; 6 Present Address: Department of Neuroscience, Mayo Clinic, Jacksonville, FL USA

**Keywords:** Chronic inflammation, Parkinson's disease, Neuroimmunology

## Abstract

Parkinson’s disease (PD) and related synucleinopathies are characterized by chronic neuroinflammation leading to the premise that anti-inflammatory therapies could ameliorate synucleinopathy and associated sequelae. To test this idea, we used recombinant adeno-associated viruses (AAV) to express the anti-inflammatory cytokine, Interleukin (Il)-10, in Line M83 transgenic mice that expresses the PD-associated A53T mutant human α-synuclein (αSyn). Contrary to our expectations, we observed that intraspinal Il-10 expression initiated at birth upregulated microgliosis and led to early death in homozygous M83+/+ mice. We further observed that Il-10 preconditioning led to reduced lifespan in the hemizygous M83+/− mice injected with preformed αSyn aggregates in hindlimb muscles. To determine the mechanistic basis for these adverse effects, we took advantage of the I87A variant Il-10 (vIl-10) that has predominantly immunosuppressive properties. Sustained intraspinal expression of vIl-10 in preformed αSyn-aggregate seeded M83+/− mice resulted in earlier death, accelerated αSyn pathology, pronounced microgliosis, and increased apoptosis compared to control mice. AAV-vIl-10 expression robustly induced p62 and neuronal LC3B accumulation in these mice, indicating that Il-10 signaling mediated preconditioning of the neuraxis can potentially exacerbate αSyn accumulation through autophagy dysfunction in the neurons. Together, our data demonstrate unexpected adverse effects of both Il-10 and its immunosuppressive variant, vIl-10, in a mouse model of PD, highlighting the pleiotropic functions of immune mediators and their complex role in non-cell autonomous signaling in neurodegenerative proteinopathies.

## Introduction

α-synucleinopathies are a heterogeneous group of disorders that are characterized by intracellular accumulation of α-synuclein (αSyn). These disorders, such as Parkinson’s disease (PD), multiple systems atrophy (MSA), and dementia with Lewy body (DLB), are associated with robust neuroinflammation^1,2^. Genome-wide association studies and transcriptome analysis have uncovered increased genetic risk of PD in patients carrying specific variants of the major histocompatibility complex (MHC) class II genes and the LRRK2 gene, suggesting that immune pathways influence disease pathogenesis^3–5^. Several studies have reported increased inflammatory plasma cytokines as well as altered T-cell profiles in PD patients associated with specific inflammatory cytokines^6,7^. Preclinical modeling studies in αSyn transgenic mice have shown that αSyn activates immune cells by binding to Toll-like receptors and receptors on T cells^8,9^. These studies confirmed that extracellular αSyn functions as damage-associated molecular patterns (DAMP), resembling pathogen-associated agents that trigger innate immunity^10^. Collectively, cohort analysis data, as well as experimental modeling studies, strongly suggest pathological synergism between chronic neuroinflammation and synuclein proteinopathy leading to the premise that neuroinflammation is a molecular driver in synucleinopathies^11^.

A major immuno-regulator during pathogen infections in the peripheral immune system is interleukin (Il)-10^12^. Il-10 modulates the activity of monocytes and macrophages leading to suppression of inflammatory cytokine expression and establishing anti-inflammatory conditions that foster tissue repair following pathogenic virulence. Based on the hypothesis that chronic neuroinflammation underlies the etiology of synucleinopathy and extracellular αSyn has DAMP-like properties, anti-inflammatory mediators such as Il-10 would be expected to ameliorate αSyn proteinopathy by dampening neuroinflammation. To test this hypothesis, we injected adeno-associated viruses (AAV) expressing Il-10 into neonatal homozygous Line M83+/+ mice or neonatal hemizygous M83+/− mice injected with preformed αSyn aggregates^13–15^. Contrary to our expectations, we found that Il-10 preconditioning reduced lifespan in both mouse models, without altering the pathological burden of αSyn. As Il-10 can have pleiotropic properties, we next used the anti-inflammatory variant, I87A variant (v)-Il-10^16,17^, to fully characterize this unexpected outcome of Il-10 signaling. We found that sustained expression of vIl-10 in hemizygous M83+/− mice seeded with preformed αSyn aggregates was also detrimental but in a manner distinct from Il-10 and more consistent with accelerated αSyn pathology. We further identified neuronal autophagic dysfunction as a possible mechanism underlying the injurious outcome of vIl-10 signaling in αSyn-aggregate-seeded mice. Together these studies document the unexpected adverse effects of Il-10 preconditioning in mouse models of αSyn pathology.

## Results

### Il-10 increases microgliosis and accelerates death in homozygous M83+/+ mice

Recent evidence has established that a robust inflammatory milieu is associated with neurodegenerative phenotype in several proteinopathies, including Parkinson’s disease (PD)^11^. Therefore, we wanted to examine whether suppressing inflammation would have a beneficial effect on proteinopathy and survival in the transgenic Line M83 mice that expresses the PD-associated A53T mutant human αSyn (*SNCA*). Since Il-10 has been shown to attenuate Th1 type immune response and moderate macrophage/monocyte activation^12^, we hypothesized that Il-10 expression would regulate inflammation leading to suppression of αSyn pathologies and extending lifespan in these mice. We used recombinant AAV serotype 1 to express mouse Il-10 under the control of hybrid chicken β-actin promoter, which was injected in the lumbar segment of the spinal cord of neonatal mice as previously described^18^. For the initial dose-finding study, we tested three different doses of AAV-Il-10 delivered into the lumbar spinal cord of nontransgenic (nonTG) mice on neonatal day P0 (Supplementary Fig. 1a–c). We found that injection of the highest dose (1 × 10^10^ viral genomes) resulted in consistent levels of Il-10 protein detectable in the soluble lysates of the spinal cord and CSF (Supplementary Fig. 1a, b), suggesting that the Il-10 was being secreted following cellular production. Further experiments were conducted using this high dose. We did not find reproducibly detectable amounts of Il-10 in the serum of these injected mice (Supplementary Fig. 1c). Injection of this highest dose of Il-10 also resulted in increased astrocytosis in the brains of nonTG mice as revealed by GFAP immunoblotting and immunohistochemistry (Supplementary Fig. 1d–f).

**Fig. 1:**
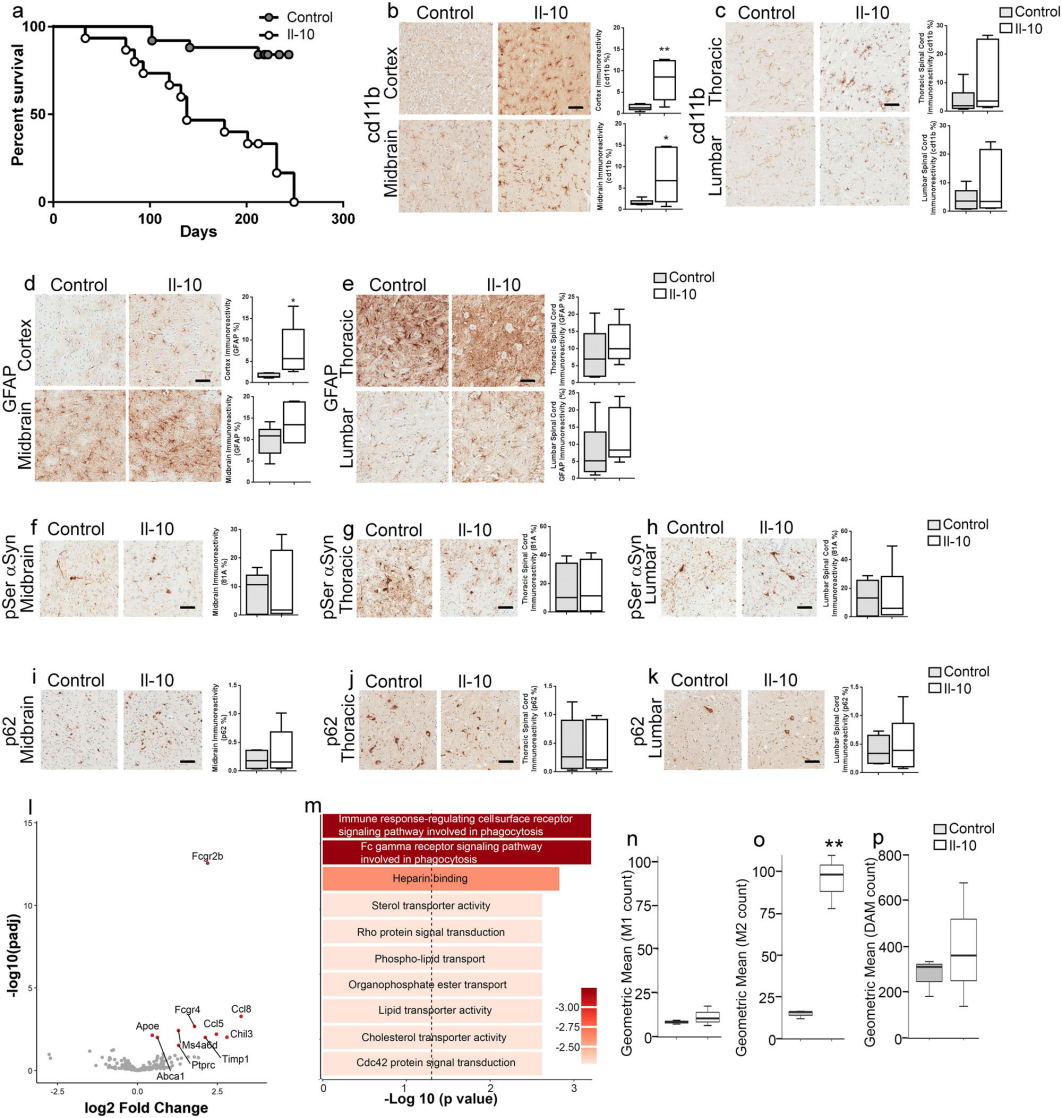
Intraspinal Il-10 expression accelerates death in homozygous M83 mice. **a** Intraspinal expression of AAV-Il-10 leads to early mortality in M83+/+ mice. The median age of survival of Il-10-expressing mice was 138 days, and the median age for the AAV-GFP-expressing mice was undefined (log-rank test, *P* < 0.0001, *n* = 15–16 mice/group). **b**, **c** Microgliosis (cd11b staining) in the brain (**b**, cortex and midbrain) and spinal cord (**c**, thoracic and lumbar segment) of M83+/+ mice. **d**, **e** Astrocytosis (GFAP staining) in the brain (**d**, cortex and midbrain) and spinal cord (**e**, thoracic and lumbar segment) of M83+/+ mice. **f**–**k** Representative images from anti-81A (**f**–**h**) and anti-p62 (**i**–**k**) antibody-stained tissue. Immunostaining analysis is shown from the midbrain (**f**, **i**), the thoracic segment of the spinal cord (**g**, **j**), and the lumbar segment of the spinal cord (**h**, **k**). Scale bar: 50 µm. *n* = 6 mice/group; two-tailed *t* test. **l**–**p** Volcano plot (**l**) showing differential expression of genes in the thoracic spinal cords of Il-10-expressing M83+/+ mice compared to GFP-expressing controls. Red dots, significantly changed genes, *P* < 0.05; gray dots, *P* > 0.05. To avoid skewing the graph, the *x* axis is limited to a range of −3 to +3 and as a result, Il-10 data is not shown (log2 FC = 13.49; *P*adj value = 1.64 × 10^−51^). Gene ontology analysis of overrepresented categories using a list of significantly changed genes from panel l (*P*adj < 0.05; dotted line) (**m**). *n* = 3 mice/group. FDR < 0.05. *P*adj = *P* values adjusted for multiple comparisons. **n**–**p** M1, M2-, and DAM-phenotype profile of microglia in Il-10-expressing M83+/+ mice compared to GFP-expressing M83+/+ control mice. **P < 0.01. *n* = 3 mice/group. In the box plot, the whiskers extend from the minimum to maximum values, with the midline representing the median.

To test the effect of Il-10 signaling on synucleinopathy, we delivered AAV-Il-10 and AAV-green fluorescent protein (GFP) into the spinal cords of neonatal homozygous M83+/+ mice^13^. We detected 7.8 ± 2.6 ng/ml of Il-10 protein in the soluble fraction of spinal cord lysates of AAV-Il-10 injected mice compared to 0.46 ± 0.4 ng/ml of Il-10 protein in control mice injected with AAV-GFP (*n* = 3 mice/group; *P* < 0.01). As these mice aged, the Il-10-expressing M83+/+ mice exhibited an abnormal phenotype marked by hunched posture, loss of body weight (>20%), labored breathing, and unusually high instances of sudden death. Compared to the GFP-expressing control cohort that exhibited ~20% death by 250 days of age, the median age of survival of Il-10-expressing M83+/+ mice was 138 days (Fig. 1a; *P* < 0.0001). We found that Il-10 expression resulted in increased microgliosis (measured by cd11b immunoreactivity) in the cortex and midbrain areas relative to phenotypically matched AAV-GFP mice (Fig. 1b; cortex, *P* < 0.01; midbrain, *P* < 0.05). Microgliosis was not significantly upregulated in either thoracic or lumbar segments of spinal cords of the Il-10-expressing M83+/+ mice (Fig. 1c). Astrocytosis (measured by GFAP immunoreactivity) was upregulated in the cortex of Il-10-expressing mice (Fig. 1d; *P* < 0.05), while midbrain and spinal cord did not show significant changes compared to GFP-expressing control mice (Fig. 1d, e). We wanted to examine whether the early death triggered by Il-10 was accompanied by the accelerated formation of αSyn inclusions in the CNS of M83+/+ mice. We used the pSer129-αSyn epitope-specific antibody to detect pathologic αSyn inclusions and the p62 antibody as a general marker of autophagy failure and protein aggregation in these tissues. Using the pSer129-αSyn-specific 81A antibody (Fig. 1f–h) or anti-p62 antibody (Fig. 1i–k), we did not observe any significant induction of αSyn pathology in the Il-10-expressing mice compared to GFP-expressing controls, suggesting that Il-10-mediated immune dysregulation can cause neurotoxicity and accelerated death independent of obvious proteinopathy abnormalities.

To understand the mechanism underlying Il-10-induced early death, we used a custom NanoString codeset^19^ to analyze gene expression changes in the thoracic spinal cords of Il-10 and GFP-expressing M83+/+ mice that showed end-stage sickness and paralysis phenotype. We identified several genes that were upregulated in the Il-10 mice—Fcgr2b, Ccl8, Chil3, Ccl5, Fcgr4, Ms4a6d, Abca1, Ptprc (Cd45), and Apoe—that broadly indicated an immune response associated with phagocytosis related pathways and Fc gamma receptor signaling pathways (Fig. 1l, m and Supplementary Table 1). Il-10 was upregulated (log2 fold change = 13.49; *P* = 1.64 × 10^−51^) in these mice but was omitted from the volcano plot in Fig. 1l as it skewed the plot (Fig. 1l and Supplementary Table 1). We were curious whether the accelerated death phenotype in Il-10-expressing mice was mediated via transcriptional upregulation of the human *SNCA* transgene driven by mouse prion promoter. We found that Il-10 expression did not change the levels of the *SNCA* transgene (log2 fold change = −0.16; *P* = 0.55) in AAV-Il-10-expressing M83+/+ mice relative to AAV-GFP-expressing M83+/+ mice (Supplementary Table 1). To establish the specific type of microglial profile associated with sustained Il-10 expression in M83+/+ mice, we calculated gene expression scores corresponding to the prevalence of inflammatory phenotype (M1 phenotype), anti-inflammatory phenotype (M2 phenotype), and neurodegeneration-specific damage-associated microglial phenotype (DAM phenotype). These scores were imputed based on expression levels of a set of genes that uniquely characterize each of these phenotypes (reviewed in ref. ^20^) (Fig. 1n–p and Supplementary Table 2). Compared to GFP-expressing M83+/+ mice, we observed that Il-10-expressing mice showed a transcriptional profile consistent with M2-type signature (Fig. 1o; *P* < 0.01). We did not observe any significant changes in the M1-type or DAM signature (Fig. 1n, p). We next compared the top altered genes in the Il-10-expressing M83+/+ mice (normalized to GFP-expressing M83+/+ mice) (Supplementary Table 1) with paralyzed aged M83+/+ mice (normalized to nonTG mice) (Supplementary Table 3). A number of common genes were identified in both groups, such as Fcgr2b, Fcgr4, Ccl5, and Ms4a6d, that are involved in immune signaling (Supplementary Table 4). Such commonalities between these two groups suggest that Il-10-mediated immune dysfunction reinforced the neurodegenerative cascade in the M83+/+ model.

### Il-10 expression reduces lifespan in preformed αSyn fibril-seeded hemizygous M83+/− mice

Next, we examined whether Il-10 preconditioning would modulate the induction and prion-like transmission of αSyn pathology in hemizygous M83+/− mice. In this model, preformed αSyn fibrils injected into the gastrocnemius muscle of 2-month-old M83+/− mice leads to progressive induction of αSyn pathology along the spinal axis and midbrain, with hindlimb paralysis occurring ~4 months after injection^14,15^.

**Fig. 2:**
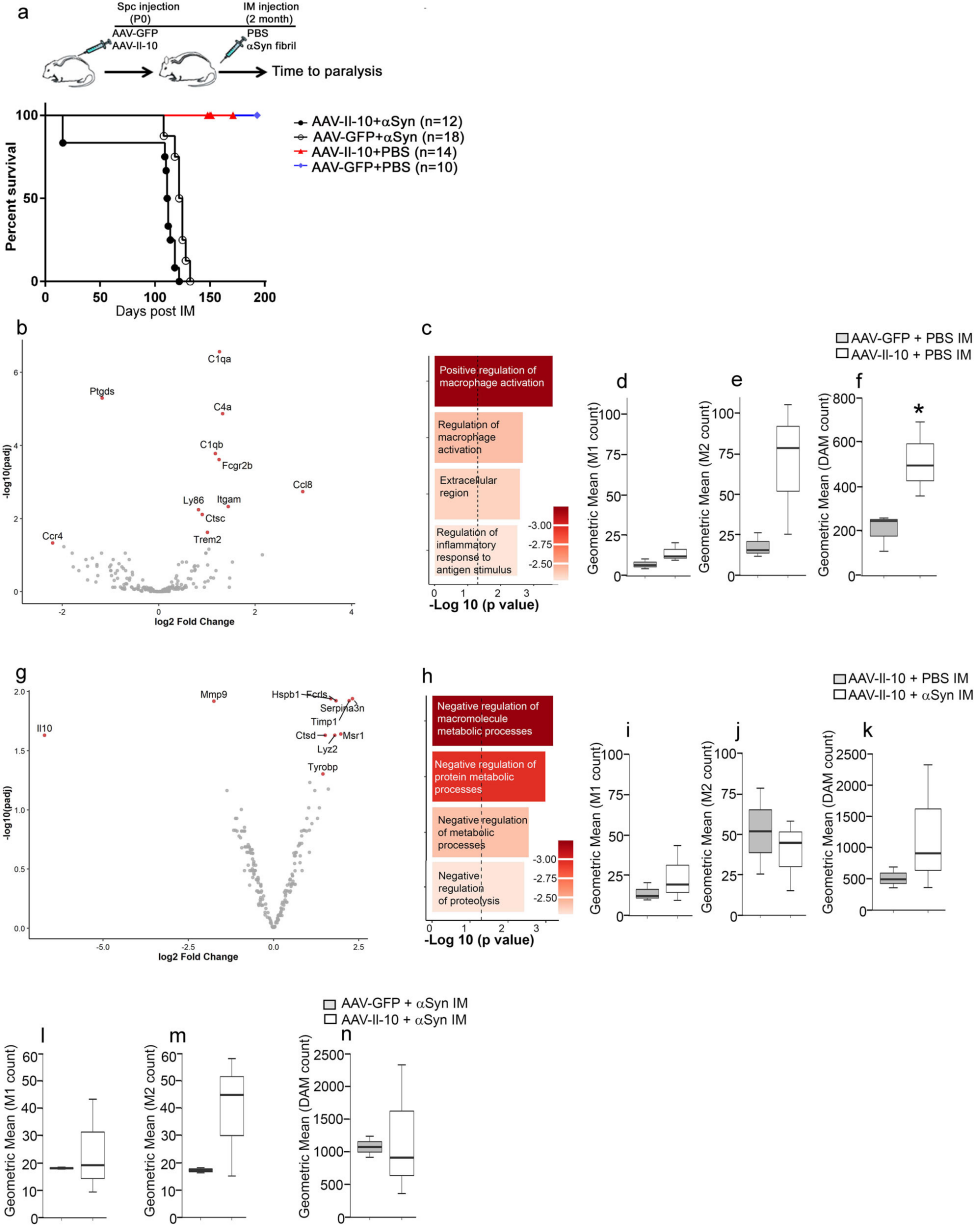
Intraspinal expression of AAV-Il-10 leads to accelerated mortality in αSyn-aggregate-seeded hemizygous M83**+**/− mice. **a** Schematic summarizing the experimental procedure (top panel). M83+/− neonates were injected intraspinally (Spc) with AAV-Il-10 or AAV-GFP (control). Mice were aged to 2 months and injected intramuscularly (IM) with preformed αSyn fibrils or PBS (vehicle control). Mice were aged to paralysis. The median age of survival of αSyn-seeded Il-10 or GFP-expressing mice was 112 days and 124.5 days post αSyn seeding respectively (log-rank test, *P* < 0.001, *n* = 10–18/group). **b–n** NanoString analysis of the αSyn or vehicle (PBS)-seeded thoracic spinal cords of M83+/− mice expressing Il-10 or GFP. **b**, **c** Volcano plot of differentially expressed genes (**b**) and gene ontology pathway analysis of overrepresented functional categories (**c**) accompanying Il-10 overexpression in M83+/− spinal cords (*P*adj < 0.05; dotted line). To avoid skewing the volcano plot, the *x* axis is limited to a range of −2.5 to +4 and as a result, Il-10 data is not plotted (log2 FC = 10.537; *P*adj value = 5.9e-07). **d**–**f** M1-, M2-, and DAM-type microglial profiling in Il-10-expressing M83+/− mice. Two-tailed *t* test, *P < 0.05. **g**, **h** Volcano plot of differentially expressed genes (**g**) and gene ontology pathway analysis of overrepresented functional categories (**h**; *P*adj < 0.05, dotted line) in αSyn-seeded Il-10-expressing M83+/− mice vs vehicle-seeded Il-10-expressing M83+/− mice. **i**–**k** M1-, M2-, and DAM-type microglial profiling in Il-10-expressing M83+/− mice in the presence or absence of intramuscular (IM) αSyn seeding. **l**–**n** M1-, M2-, and DAM-type microglial profiling in Il-10 vs GFP-expressing αSyn-seeded M83+/− mice. *n* = 3 mice/group. Red dots significantly changed genes, *P* < 0.05 and gray dots, *P* > 0.05 in volcano plots after adjusting for multiple testing and FDR < 0.05. In the box plot, the whiskers extend from the minimum to maximum values, with the midline representing the median.

Neonatal M83+/− mice were injected with AAV-Il-10 or AAV-GFP in the lumbar spinal cord and aged to 2 months when they were injected bilaterally in the hindlimb muscle with preformed αSyn fibrils (Fig. 2a). As the control for preformed αSyn fibril injection, mice were injected with the vehicle, PBS, in their hindlimb muscle. Biochemical analysis of the spinal cord lysates of mice revealed that at end stage, the αSyn-seeded AAV-Il-10 and AAV-GFP-expressing M83+/− mice had 4.2 ± 1.4 ng/ml and 1.6 ± 0.06 ng/ml of Il-10 protein, respectively (*n* = 5 mice/group; *P* < 0.01). In the αSyn-seeded cohorts, we observed that Il-10 reduced lifespan compared to the control group expressing GFP (Fig. 2a; median survival of Il-10 mice = 112 days and control mice = 124.5 days post injection; *P* < 0.0001). Non-seeded control mice (injected with vehicle PBS) expressing AAV-GFP or AAV-Il-10 did not develop paralysis (Fig. 2a).

Using the custom neurodegeneration NanoString codeset, we examined gene expression changes in M83+/− mice expressing Il-10. First, we examined the gene expression changes in PBS injected (non-seeded) M83+/− mice injected with AAV-Il-10 or AAV-GFP. We found that Il-10 expression leads to upregulation of various immune genes, such as complement factors, Ccl8, Fcgr2b, and Trem2 (Fig. 2b and Supplementary Table 5). Il-10 was also robustly upregulated (log2 fold change = 10.53; *P* = 5.89 × 10^−7^) in these mice but was omitted from the volcano plot in Fig. 2b as it skewed the plot (Fig. 2b and Supplementary Table 5). GO pathway analysis indicates that Il-10 expression induced robust inflammatory signaling characteristic of macrophage activation and presence of an antigenic stimulus (Fig. 2c). Neither M1-type nor M2-type gene expression signatures were significantly enhanced in these mice, though there was a suggestive trend in the M2 signature (Fig. 2d, e; *P* = 0.32 for M2). Interestingly, we observed that Il-10 expression led to induction of DAM signature (Fig. 2f; *P* < 0.05), primarily driven by increased expression of Trem2.

Next, we compared the gene expression changes in Il-10-expressing M83+/− mice that had been seeded with preformed αSyn aggregates relative to Il-10-expressing non-seeded (PBS injected) M83+/− mice (Supplementary Table 6). Here, we found that αSyn seeding in the presence of Il-10 results in an exacerbated inflammatory milieu characterized by increased Lyz2, Msr1, Ctsd, and Tyrobp (Fig. 2g and Supplementary Table 6). Overall, GO pathway analysis indicated reduced metabolic processes at different cellular and physiologic levels, indicative of a degenerative signature (Fig. 2h). There were no significant changes in M1-, M2-, or DAM-type profiles in the αSyn-seeded mice compared to mice injected with PBS vehicle control (Fig. 2i–k).

When we compared the DEG in αSyn-seeded M83+/− mice expressing Il-10 to αSyn-seeded M83+/− mice expressing GFP at end stage, we observed that both the DEGs were extremely similar to each other such that there were no differentially expressed genes that could be identified from this comparison (Supplementary Table 7). Consistent with this observation, microglial profiling analysis did not show significant levels of difference between the two groups (Fig. 2l–n). To understand this neurodegenerative cascade, we performed an additional comparison. There were ten genes that were significantly altered in response to αSyn seeding in Il-10-expressing mice (Il-10-expressing αSyn-seeded mice normalized to Il-10-expressing non-seeded mice) (Fig. 2g and Supplementary Table 6). When we compared this list with genes altered in αSyn-seeded naive M83+/− mice normalized to non-seeded mice (Supplementary Table 8), we found that the differentially expressed genes (except for Il-10) were represented in both the cases (Supplementary Table 9). The similarities in the list of altered genes indicate that the cellular pathways affected by Il-10 preconditioning synergized with the normally ongoing neurodegenerative cascade in the αSyn-seeded M83+/− mice.

### An immunosuppressive variant of cellular Il-10 reduces lifespan in preformed αSyn fibril-seeded M83+/− mice

Il-10 has pleiotropic properties—in addition to its well-recognized immunosuppressive properties, it also has immunostimulatory functions^21^. For example, Il-10 can exert stimulatory effects on lymphocytes, murine thymocytes, and murine mast cells^22,23^. Indeed, our gene expression data identified several genes indicative of an activated immune signature, such as Ccl8, Ly86, and Itgam, in Il-10-expressing mice (Fig. 2b). In order to understand whether the unexpected detrimental phenotype in Il-10-expressing M83+/− mice was related to the immunosuppressive or immunostimulatory function of Il-10, we decided to use a variant Il-10 (vIl-10) that lacks immunostimulatory function^16,17^.

**Fig. 3:**
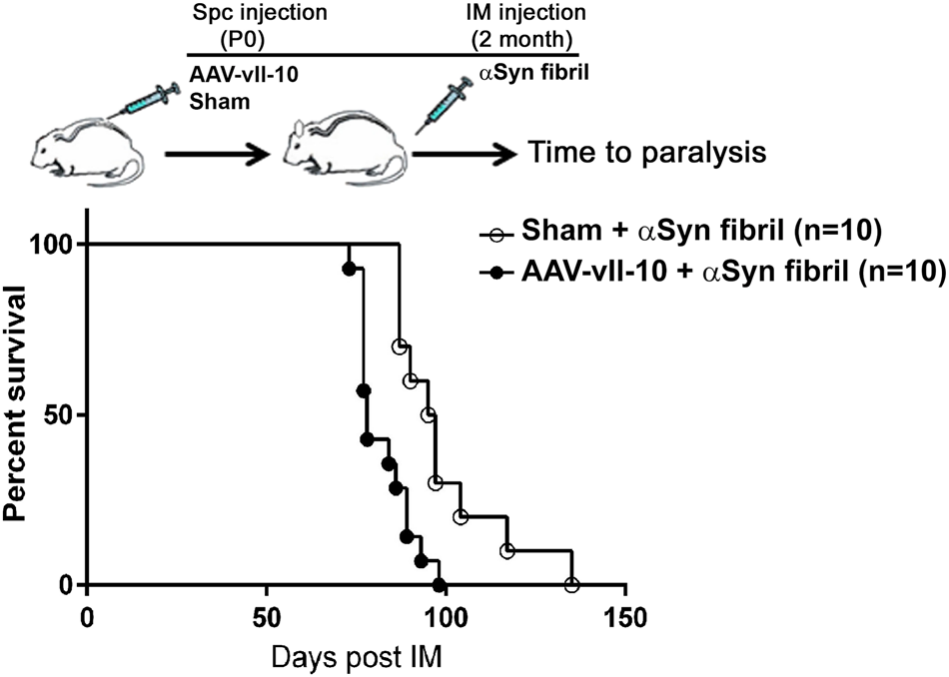
Intraspinal expression of AAV-vIl-10 leads to early mortality in αSyn-seeded M83**+**/− mice. M83+/− neonates were intraspinally injected with AAV-vIl-10 or sham injected, aged to 2 months, and then injected with αSyn fibril in hindlimb muscles at 2 months of age. The median age of survival of vIl-10-expressing mice was 78 days post αSyn fibril injection, while the median age of sham injected mice was 96 days (log-rank test, *P* < 0.0019, *n* = 10 mice/group).

Some viruses have appropriated the immunosuppressive properties of Il-10 by expressing close homologs that could be beneficial in preserving viral pathogenesis through ineffective host immune response^17^. One such homolog, produced by the Epstein Barr Virus and called *BCRF-I*, encodes a single point mutation in cellular Il-10 (I87A Il-10), referred to as vIl-10^16^. This vIl-10 has lost the stimulatory activities of cellular Il-10 while retaining its immunosuppressive properties. In the next experiment, we delivered AAV-vIl-10 into neonatal M83+/− mice, aged these to 2 months, seeded with preformed αSyn fibrils in the hindlimb muscle and aged them to paralysis. Analysis of the RIPA-soluble spinal cord lysates showed that the vIl-10 levels in the AAV-vIl-10 and control mice were 5.08 ± 0.91 pg/ml and 0.47 ± 0.13 pg/ml, respectively (*n* = 3 mice; *P* = 0.007). In this cohort of αSyn-seeded mice, we observed that vIl-10 expression resulted in an accelerated paralysis phenotype (Fig. 3; *P* < 0.0019). This data shows that an anti-inflammatory variant of Il-10 robustly reduces survival in αSyn-seeded M83+/− mice.

### vIl-10 expression exacerbates microgliosis in preformed αSyn fibril-seeded M83+/− mice

**Fig. 4:**
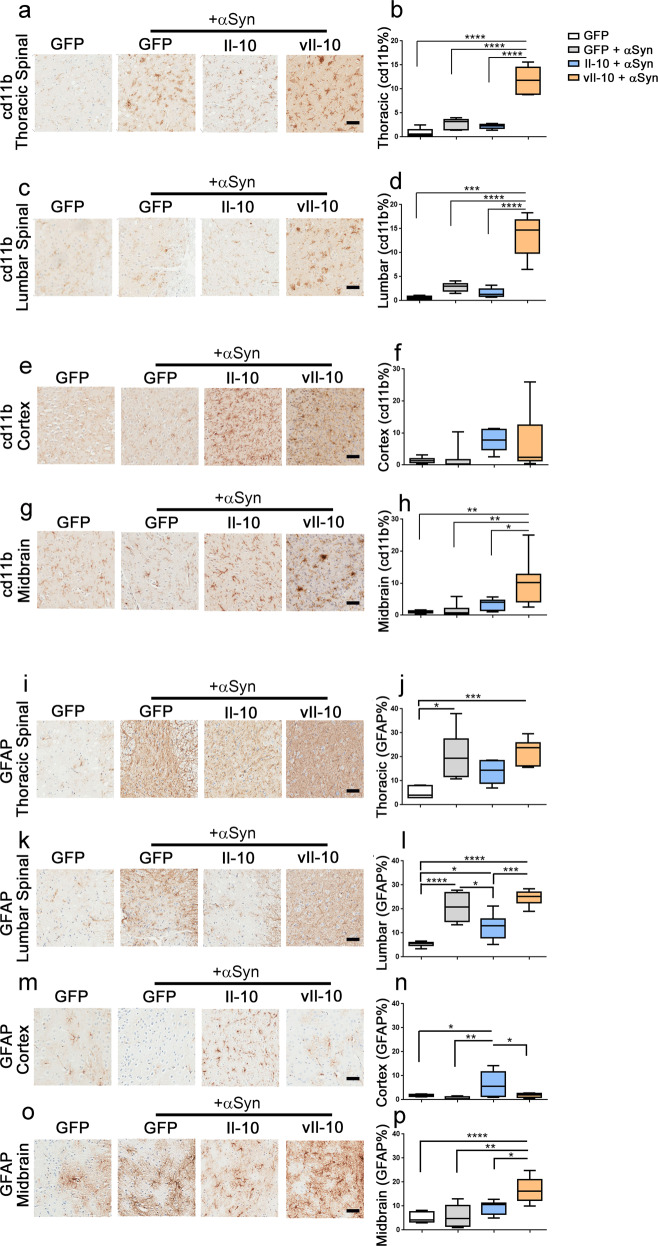
Intraspinal vIl-10 expression induces microgliosis but not astrogliosis in spinal cords of αSyn fibril-seeded hemizygous M83**+**/− mice. Neonatal M83+/− mice were injected with AAV-GFP, AAV-Il-10, or AAV-vIl-10 and were aged to 2 months when they were injected with preformed αSyn fibrils in the muscle. PBS (vehicle) injection was the control for αSyn fibril injection and is represented by the immunohistochemistry panel on the far left of each panel. **a**–**h** Representative cd11b staining patterns and immunostaining analysis (% immunoreactivity burden) for microgliosis in thoracic segment of the spinal cord (**a**, **b**), lumbar segment of the spinal cord (**c**, **d**), cortex (**e**, **f**), and midbrain (**g**, **h**). **i**–**p** Representative GFAP staining and immunostaining analysis (% immunoreactivity burden) for astrogliosis in thoracic segment of the spinal cord (**i**, **j**), lumbar segment of the spinal cord (**k**, **l**), cortex (**m**, **n**), and midbrain (**o**, **p**). The whiskers in the box plot extend from the minimum to maximum values, with the midline representing the median. *n* = 6–7 mice/group; one-way ANOVA; ****P < 0.0001; ***P < 0.001; **P < 0.01; *P < 0.05. Scale bar, 50 µm.

We next conducted an extensive neuropathological analysis to characterize how Il-10 and vIl-10 expression modulate the induction and transmission of the αSyn pathology in hemizygous M83+/− mice. We examined microgliosis using cd11b antibody in the spinal cords (Fig. 4a–d) and brains (Fig. 4e–h) of αSyn-seeded M83+/− mice. Contrary to our expectation that vIl-10 would dampen gliosis, we observed robust microgliosis in vIl-10-expressing αSyn-seeded mice in both thoracic and lumbar segments of the spinal cord compared to control GFP-expressing PBS-injected mice, GFP-expressing αSyn-seeded mice or Il-10-expressing αSyn-seeded mice (Fig. 4a, b, *P* < 0.0001 against all groups in the thoracic segment; Fig. 4c, d, *P* < 0.001 against control and *P* < 0.0001 against αSyn-seeded mice in the lumbar segment). While vIl-10 expression also enhanced microgliosis in the midbrain area (Fig. 4g, h; *P* < 0.05 compared to Il-10-expressing αSyn-seeded mice and *P* < 0.01 compared to GFP-expressing αSyn-seeded mice), there was no difference observed in the cortex relative to other mouse cohorts (Fig. 4e, f). We did not observe increased microglia numbers in the Il-10-expressing αSyn-seeded mice relative to control αSyn-seeded mice in the spinal cord and the brain (Fig. 4a–h).

### Il-10 expression suppresses astrocytosis in spinal cords of αSyn-seeded M83+/− mice

We observed a complex pattern of astrocytosis in the αSyn-seeded hemizygous M83+/− mice (Fig. 4i–p). GFAP immunostaining in the thoracic and lumbar spinal cord showed that compared to PBS injected GFP-expressing hemizygous M83+/− mice, αSyn seeding induced astrocytosis in GFP-expressing mice as expected (Fig. 4i–l; *P* < 0.05 in the thoracic segment and *P* < 0.0001 in the lumbar segment). Within the αSyn-seeded cohorts, compared to GFP or vIL-10-expressing mice, expression of Il-10 reduced astrocytosis in the lumbar spinal cord (Fig. 4k, l; *P* < 0.05 against GFP and *P* < 0.001 against vIl-10), and there was a suggestive lowering trend in the thoracic spinal cord (Fig. 4i, j). In general, we observed that vIl-10 did not induce astrocytic proliferation in the spinal cord compared to the GFP-expressing mice that were seeded with preformed fibrils (Fig. 4j, l). In the brain, αSyn seeding, by itself, did not significantly alter astrocytosis in GFP-expressing mice (Fig. 4m–p). In the cortex, the Il-10-expressing mice had a higher astroglial burden compared to all other groups examined including vIl-10-expressing mice (Fig. 4m, n; *P* < 0.05 relative to vIl-10 and *P* < 0.01 relative to control seeded mice), consistent with our data in non-seeded homozygous M83+/+ mice (Fig. 1d). In the midbrains of mice seeded with preformed fibrils, we observed that vIl-10 expression caused the highest level of astrocyte proliferation compared to non-seeded (PBS-injected) and αSyn-seeded control mice (Fig. 4o, p; *P* < 0.0001 against non-seeded mice; *P* < 0.01 and *P* < 0.05 against seeded GFP-expressing and Il-10-expressing mice respectively). This finding suggests that there is a selective vulnerability of astrocytes in different areas of the neuraxis to Il-10- and vIl-10-mediated signaling.

### vIl-10 worsens pathological pSer129-αSyn inclusion pathology and increases αSyn levels

**Fig. 5:**
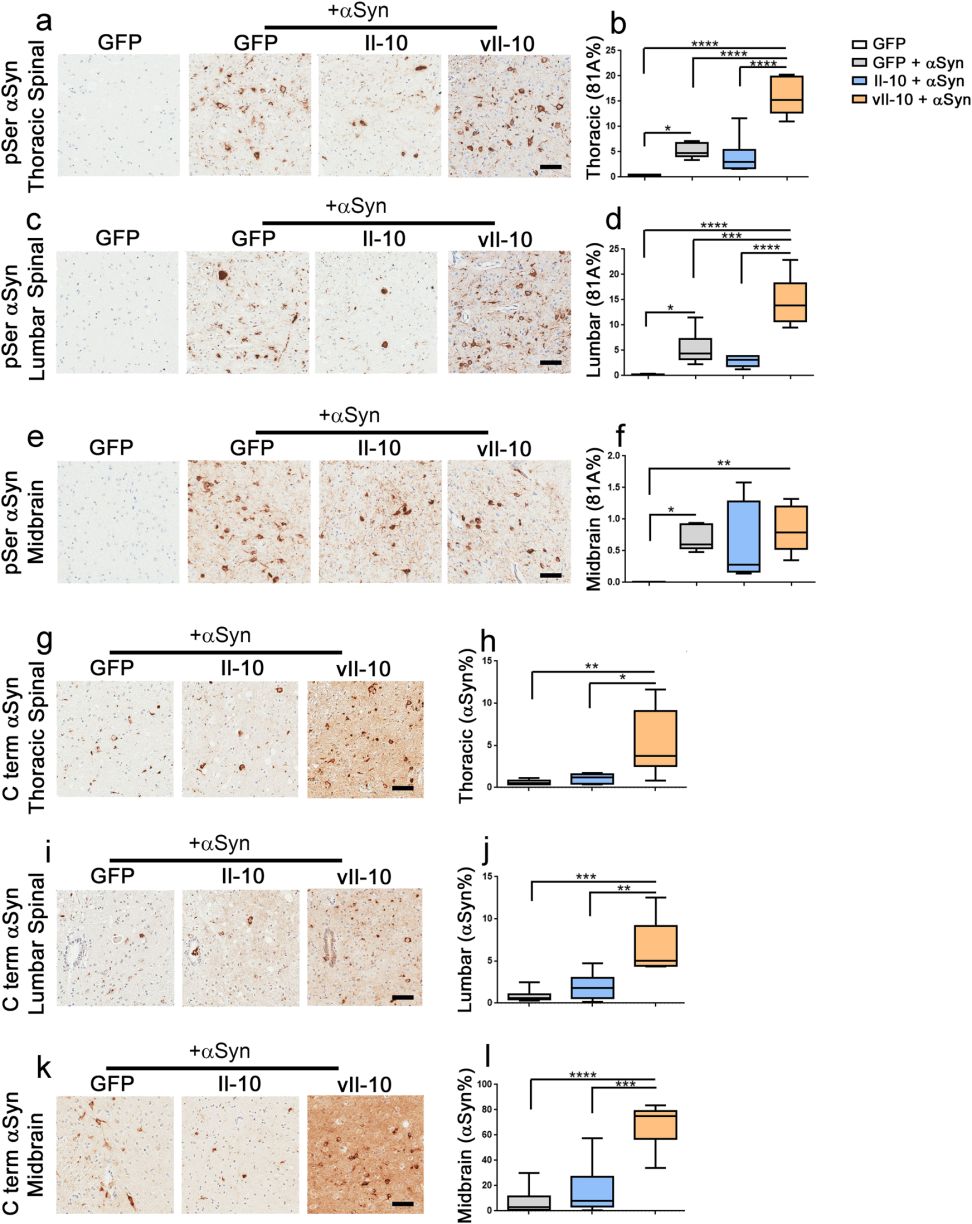
vIl-10 induces pathological αSyn inclusions and parenchymal αSyn accumulation in αSyn fibril-seeded hemizygous M83**+**/− mice. Neonatal M83+/− mice were injected with AAV-GFP, Il-10, or vIl-10 and were aged to 2 months when they were injected with αSyn fibrils in the muscle. PBS (vehicle) injection was the control for αSyn fibril injection and is represented by the immunohistochemistry panel on the far left (**a**, **c**, **e**). Representative images and % immunoreactivity burden analysis of 81A antibody (specific to pSer129-αSyn epitope) stained tissue from the thoracic and lumbar segment of the spinal cord (**a**–**d**) and midbrain (**e**, **f**). Representative images and % immunoreactivity burden analysis from 15-4E7 antibody (specific to the C terminus of αSyn) stained spinal cord (**g**–**j**) and midbrain (**k**, **l**). In the box plot, the whiskers extend from the minimum to maximum values, with the midline representing the median. *n* = 5–7 mice/group; one-way ANOVA; ****P < 0.0001; ***P < 0.001; **P < 0.01; *P < 0.05. Scale bar, 50 µm.

As both Il-10 and vIl-10 caused accelerated paralysis in hemizygous M83+/− mice seeded with preformed αSyn fibrils, we next examined if this phenotype correlated with exacerbated αSyn proteinopathy. Consistent with our previous data^14,15^, injection of preformed αSyn fibrils in the gastrocnemius muscle of adult hemizygous M83+/− mice expressing AAV-GFP induced pSer129-αSyn reactive intracellular inclusions in the thoracic spinal cord, lumbar spinal cord, and midbrain compared to GFP-expressing non-seeded mice (Fig. 5a–f; *P* < 0.05). These αSyn inclusions resemble Lewy bodies (LB) and were not observed in control GFP-expressing mice injected with the vehicle PBS (Fig. 5a, c, e). Expression of Il-10 did not alter the levels of pSer129-αSyn immunoreactive LB-type pathology in the spinal cord or brain, compared to GFP-expressing αSyn-seeded mice (Fig. 5b, d, f). On the other hand, intraspinal vIl-10 expression dramatically increased αSyn inclusion pathology in the thoracic and lumbar spinal cords compared to GFP-expressing and Il-10-expressing seeded mice (Fig. 5b, d; *P* < 0.0001 in thoracic; *P* < 0.001 and *P* < 0.0001 in lumbar segment respectively). In the midbrains of αSyn-seeded mice, the αSyn pathology burden was similar between all three cohorts (Fig. 5f).

The role of soluble αSyn in synucleinopathies is unclear. While features like LBs, that are characterized by clusters of aggregated αSyn inside neurons, are clearly neurotoxic, some studies have indicated that even modest increases in soluble αSyn protein levels can lead to cellular dysfunction^24^. Indeed, the early age of disease onset and disease progression severity in patients with duplication of wild-type αSyn loci indicates that simple intracellular accumulation of αSyn protein may have a profound pathogenic effect^25^. To investigate whether Il-10-associated signaling pathways affect the accumulation of total αSyn levels, we used an antibody specific to the C terminus of human αSyn, antibody 15-4E7, that was characterized previously in our lab^26^. Compared to both GFP and Il-10-expressing αSyn-seeded mice, we observed that vIl-10-expressing mice showed higher levels of 15-4E7-immunoreactive intracellular αSyn staining in both the thoracic and lumbar segments of the spinal cord (Fig. 5g–j; *P* < 0.05 relative to Il-10 and *P* < 0.01 relative to GFP for the thoracic segment; *P* < 0.01 relative to Il-10 and *P* < 0.001 relative to GFP for the lumbar segment). Most of these stained structures were intracellular with some degree of parenchymal staining visible (Fig. 5g, i, k). Increased parenchymal staining with 15-4E7 antibody in the midbrain of vIl-10-expressing mice compared to GFP-expressing mice (*P* < 0.0001) and Il-10-expressing mice (*P* < 0.001) indicates dramatic accumulation of extracellular αSyn in this brain area (Fig. 5k, l).

### vIl-10 induces cell death in spinal cords of αSyn fibril-seeded M83+/− mice

**Fig. 6:**
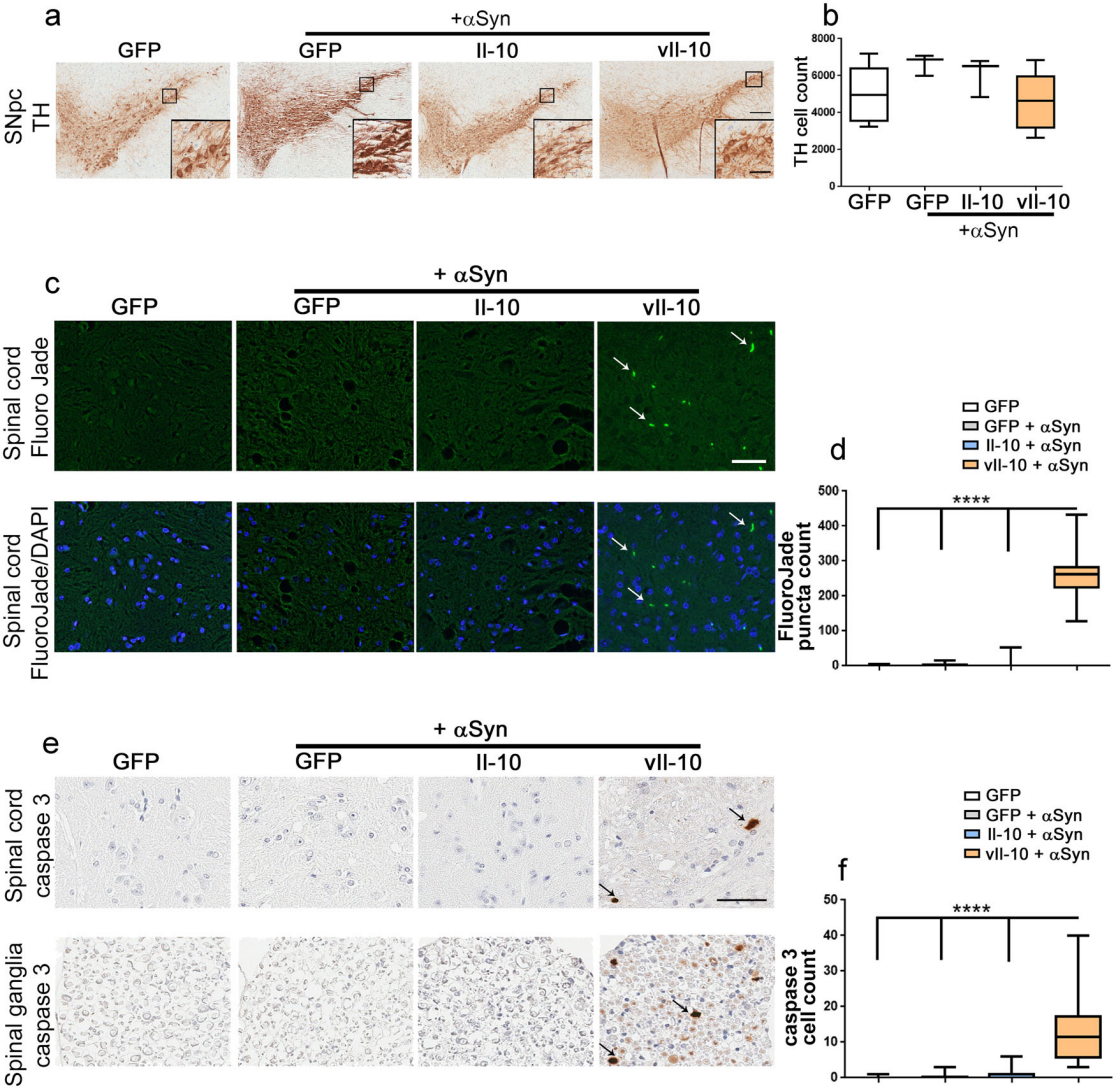
Cell death analysis in αSyn aggregate-seeded hemizygous M83+/− mice. **a**, **b** Midbrains of vehicle-injected AAV-GFP-expressing M83+/− mice or αSyn aggregate-seeded M83+/− mice expressing AAV-GFP, AAV-Il-10, or AAV-vIl-10 were stained for tyrosine hydroxylase (TH) (**a**), and the number of TH-positive dopaminergic neuronal cell bodies was manually counted (**b**). Scale bar, 200 µm (main panel), 50 µm (inset). *n* = 3–6 mice/group; one-way ANOVA. **c**–**f** Cell death was analyzed in the lumbar spinal cord of M83+/− mice. Punctate Fluoro-Jade C staining was observed in the vIL-10-expressing αSyn-seeded M83+/− mice indicated by arrows (**c**, top panel). The bottom panel depicts a dual-color image incorporating the nuclear stain DAPI. The number of Fluoro-Jade C-labeled puncta was manually counted (**d**). Anti cleaved caspase 3 antibody was used to stain the spinal cord (**e**, top panel) and accompanying spinal ganglia (**e**, bottom panel) of mice. The number of cleaved caspase 3-positive cells from the spinal cord and spinal ganglia (arrows, **e**) was manually counted (**f**). In the box plot, the whiskers extend from the minimum to maximum values, with the midline representing the median. Scale bar, 20 µm (**c**), 50 µm (**e**). *n* = 5–7 mice/group. One-way ANOVA, ****P < 0.0001.

We wanted to examine whether Il-10-mediated signaling pathways resulted in cell death in the brain and spinal cord. Since peripheral αSyn seeding induced midbrain αSyn pathology in our mice, we first quantified whether this led to a loss in dopaminergic neurons in these mice. Using tyrosine hydroxylase (TH) as the marker of dopaminergic cell bodies in the substantia nigra, we did not observe dopaminergic cell loss in any of our αSyn-seeded mouse cohorts compared to the control mice receiving vehicle injection (Fig. 6a, b).

We also assessed the effects of the Il-10 signaling pathways on cell death in the spinal cords of M83+/− mice (Fig. 6c–f). We used two different reagents to investigate cell death in these mice. Fluoro-Jade C is an anionic dye used to detect degenerating or necrotic neurons and processes, irrespective of the type of death pathway involved^27^. Histochemical staining revealed increased Fluoro-Jade C punctate staining in the lumbar spinal cords of seeded vIl-10-expressing mice compared to control GFP-expressing mice or Il-10-expressing mice (Fig. 6c, d; *P* < 0.0001). Such punctate staining is consistent with dying dendritic or axonal terminals. We further probed for activated caspase 3 to detect apoptotic cells in the lumbar spinal cords of seeded M83+/− mice (Fig. 6e, f). We found sparse activated caspase 3 immunopositive cells in the gray matter of the spinal cord as well as the spinal ganglia of vIl-10-expressing αSyn-seeded M83+/− mice (Fig. 6e, f; *P* < 0.0001), albeit to a lesser extent than observed with Fluoro-Jade C technique. None of the other αSyn-seeded mice demonstrated any detectable Fluoro-Jade C or activated caspase 3 reactivity (Fig. 6d, f).

### vIl-10 increases autophagy dysfunction in αSyn fibril-seeded M83+/− mice

**Fig. 7:**
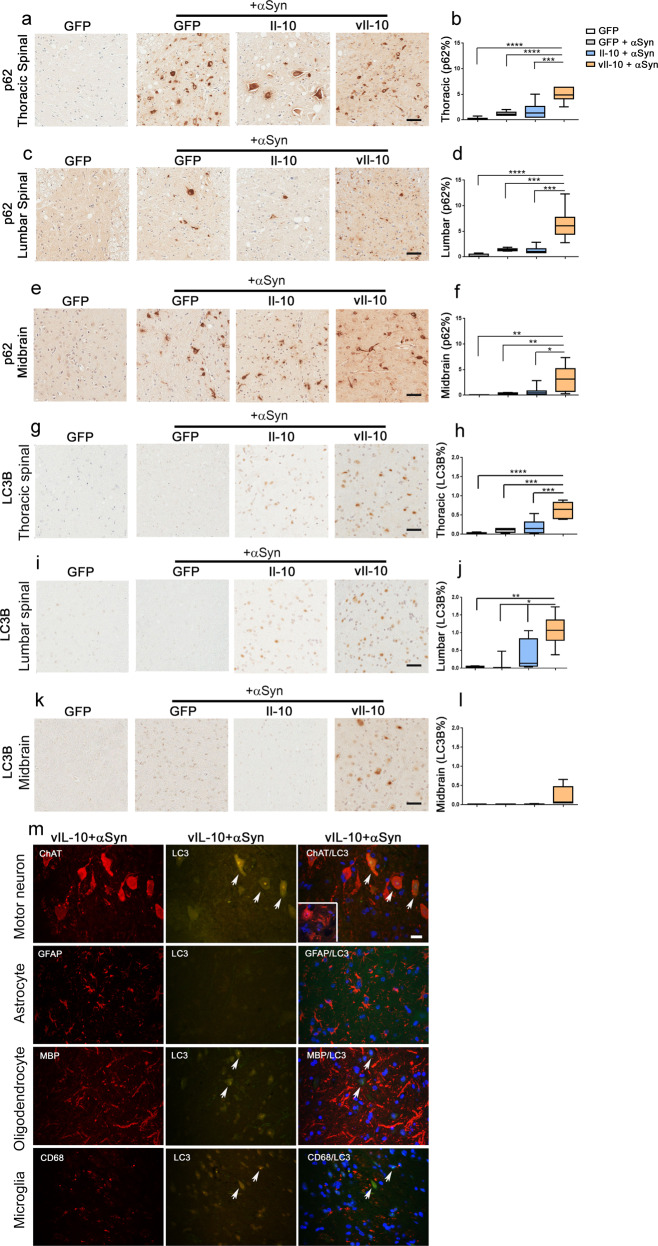
Accumulation of autophagic markers in vIl-10-expressing αSyn fibril-seeded hemizygous M83+/− mice. Neonatal M83+/− mice were injected with AAV-GFP, Il-10, or vIl-10 and were aged to 2 months when they were injected with αSyn fibrils in the muscle. PBS (vehicle) injection was the control for αSyn fibril injection and is represented by the immunohistochemistry panel on the left of each panel. Representative images of anti-p62/sequestosome antibody-stained tissue and immunostaining analysis (% immunoreactivity burden) from the thoracic spinal cord (**a**, **b**), lumbar spinal cord (**c**, **d**), and midbrain (**e**, **f**) are shown. Representative images from MAP1LC3B antibody-stained tissue and immunostaining analysis (% immunoreactivity burden) derived from the thoracic spinal cord (**g**, **h**), lumbar spinal cord (**i**, **j**), and midbrain (**k**, l) are shown. Of note, 2/5 mice in the Il-10-expressing seeded mice showed LC3B staining in the spinal cord. *n* = 5–7 mice/group; one-way ANOVA; ****P < 0.0001; ***P < 0.001; **P < 0.01; *P < 0.05. Scale bar, 50 µm. In the box plot, the whiskers extend from the minimum to maximum values, with the midline representing the median. **m** Representative images from the lumbar spinal cord showing robust localization of LC3B (arrows, middle panels) in ChAT-immunopositive neurons (arrows, top panel) but not in GFAP-immunopositive astrocytes, MBP-immunopositive oligodendrocytes, or CD68-immunopositive microglia of vIl-10-expressing Line M83+/− mice seeded with αSyn fibril. The right panels show merged images of LC3B (detected with Alexa fluor 488 nm) with cell type-specific markers (detected with Alexa fluor 594 nm). Slides were counterstained with DAPI. Inset in ChAT panel (right) denotes triple-color merged image from M83+/− mice seeded with αSyn fibril showing no detectable LC3B. Arrows (denoting LC3B signal) in MBP- and CD68-stained panels do not colocalize with oligodendrocyte or microglia, respectively. Scale bar, 10 µm.

We wanted to explore whether the increased parenchymal accumulation of soluble or cell-free αSyn, as well as intracellular inclusion pathology, could be due to impaired autophagic clearance in the seeded M83+/− mice. Because of previously reported data that IL-10 can inhibit autophagy^28,29^, we reasoned that this might explain mechanistically the effect of Il-10-mediated worsening of αSyn proteinopathy. Using p62 as a marker of autophagic dysfunction^30^, we observed that αSyn seeding induced modest p62 accumulation in the spinal cords of GFP and Il-10-expressing mice (Fig. 7a–d) and the midbrain (Fig. 7e, f) compared to non-seeded genotype-matched and age-matched M83+/− mice. vIl-10, on the other hand, resulted in a profound upregulation of intracellular p62-immunoreactive inclusions in the thoracic and lumbar spinal cords (Fig. 7a–d; *P* < 0.001 relative to Il-10, *P* < 0.0001 relative to GFP) as well as in the midbrain (Fig. 7e, f; *P* < 0.05 relative to Il-10, *P* < 0.01 relative to GFP) of αSyn-seeded mice.

To further confirm autophagic disturbance in these mice, we assessed the levels of microtubule-associated protein 1 light chain 3 β (MAP1LC3B)^30^ or LC3B, a key regulatory component of autophagic degradation pathway. Cytoplasmic and nuclear accumulation of LC3B indicates abnormalities in autophagosome assembly, which is widely used for the detection of dysfunctional autophagy^31^. Anti MAP1LC3B staining revealed sparse staining in the thoracic and lumbar spinal cords of Il-10-expressing αSyn-seeded mice (Fig. 7g–j) but not in the midbrain (Fig. 7l). On the other hand, vIl-10 expression led to nuclear staining and diffuse cytoplasmic staining suggestive of accumulation of autophagic debris. Compared to GFP-expressing mice seeded with αSyn, vIl-10 mice showed increased LC3B levels in the thoracic and lumbar segments of spinal cords (Fig. 7g–j; *P* < 0.001 in the thoracic spinal cord, *P* < 0.05 in the lumbar spinal cord), whereas there was a trend toward increased LC3B staining in the midbrains (Fig. 7k, l; *P* = 0.22 relative to control αSyn-seeded mice). To identify which cells preferentially accumulated LC3B in response to vIl-10, we performed a co-immunofluorescence analysis localizing LC3B in motor neurons (stained with choline acetyltransferase, ChAT), astrocytes (stained with GFAP), oligodendrocytes (stained with myelin basic protein, MBP), and microglia (stained with CD68). Representative images from the lumbar spinal cord of vIL-10-expressing mice seeded with αSyn fibrils showed robust LC3B staining in the ChAT-immunopositive neurons (arrows, top panel, Fig. 7m). None of the other cell types tested (astrocytes, oligodendrocytes, and microglia) show appreciable LC3B colocalizing with the cell type-specific markers (Fig. 7m). Together, these observations indicate that preconditioning the neuraxis with Il-10-related immunosuppressive signaling can lead to exacerbation of αSyn proteinopathy via altering neuronal autophagic pathways.

## Discussion

Gliosis is a cardinal feature in post mortem brains of PD patients^32^ as well as a prodromal feature in the early-stage PD patients and DLB patients^33,34^. Several cytokines with stimulatory properties—for example, IL-6 and TNF-α—are increased in PD patient sera and are correlated with the severity of the nonmotor symptoms in PD patients^35^. Recent genome-wide association studies have also uncovered genetic overlaps in PD patients and patients suffering from autoimmune disorders^36^. In mouse models of synucleinopathy, we, as well as other groups, have reported robust inflammation in the end-stage mice^15,37^, and treatment with immunomodulatory agents prevented αSyn pathology^38^. These lines of evidence generally point to inflammatory signaling as a possible etiological factor in synucleinopathies. Our study was designed to test whether Il-10 overexpression initiated before the onset of neuropathology or phenotypes would be able to suppress inflammation and ameliorate αSyn pathologies in the M83 model. Our approach in this study was to use recombinant AAVs to express Il-10 based on its future translational potential as a vector for gene therapy^39^. Further, the AAV gene targeting approach allowed us to restrict Il-10 expression to the CNS, thereby limiting its effect only to the target organ and minimizing off-target effects on peripheral immunity. Contrary to our expectations, we uncovered a detrimental effect of intraspinal Il-10 expression in our synucleinopathy models. We specifically found that Il-10 overexpression in the spinal cord of homozygous M83+/+ mice leads to gliosis, moribund condition, and early death. In the second model where hemizygous M83+/− mice were seeded peripherally with preformed αSyn fibrils, we found that Il-10 expression similarly led to reduced lifespan. In both cases, Il-10-mediated accelerated death was not accompanied by increased αSyn inclusion pathology in mice phenotypically matched for paralysis with control cohorts, revealing an interesting dichotomy between neurodegenerative cascade and proteinopathy in the context of neuroinflammation. Previously, we have reported that AAV-Il-10 worsened Alzheimer’s disease (AD) pathology without affecting lifespan^19^. Intracranial Il-10 expression in this model worsened amyloid β pathology by reducing microglial phagocytic activity and increasing ApoE sequestration within the extracellular amyloid β plaques^19^. On the other hand, intraspinal AAV-Il-10 (the same construct used in this study and ref. ^19^) expression delayed paralysis and increased lifespan in the SOD1 model of ALS through moderating inflammatory chemokine signaling pathways^18^. Thus, the phenotypic and neuropathologic outcomes of Il-10 signaling were widely disparate in these different neurodegenerative models, in spite of the fact that these experiments were performed on similar genetic backgrounds of mixed C57Bl6 lineage. Taken together, these observations serve as exemplars of the immunoproteostasis paradigm, whereby the same immune mediator can differentially influence the pathologic outcome based on the type of neurodegenerative proteinopathy and the context of the proteinopathy^40^.

To disentangle the pleiotropic actions of Il-10 in the αSyn fibril-seeded M83+/− mouse model, we used a naturally occurring variant vIl-10 that has predominantly immunosuppressive properties^16^. Contrary to our expectation that this vIl-10 would ameliorate synucleinopathy, we observed dramatic upregulation of gliosis, accumulation of soluble αSyn and pathological αSyn aggregates, and neuronal-predominant autophagic impairment in vIl-10-expressing mice compared to control mice. This unexpected effect on autophagy impairment is consistent with previous observations that Il-10 can impair lymphocyte- and dendritic cell-mediated autophagy^28,29^. Interestingly, while vIl-10 and Il-10 both reduced lifespan in αSyn-seeded M83 mice, only vIl-10 exacerbated intracellular and parenchymal αSyn accumulation. Though our data primarily suggests that the injurious outcome of vIl-10 signaling in the αSyn model is mediated by neuronal autophagic impairment and a failure to clear neuronal αSyn, it is also possible that this phenotype can be influenced partly by vIl-10-induced microgliosis. Taken together, our study points to a novel aspect of immune signaling in synucleinopathies, whereby Il-10 conditioning can exacerbate the pathologic landscape through a combination of cell-autonomous and non-cell-autonomous means, as shown in other models of neurodegenerative diseases^41^.

Our study opens up an intriguing question surrounding the relative roles of immune cells in the αSyn aggregate-seeded model. Extensive immunohistochemical examination of microglial and astrocytic proliferation showed that, in spite of having predominantly immunosuppressive properties in the peripheral immune system^16,17^, vIl-10 robustly upregulated microglial number in the spinal cord and midbrain of αSyn-seeded mice coinciding with increased intracellular αSyn inclusions and parenchymal αSyn levels. First, this would suggest that preconditioning the CNS of a neurodegenerative proteinopathy model with a cytokine having immunosuppressive properties does not necessarily dampen gliosis. Second, it is tempting to suggest that chronic immunosuppression can result in reduced microglial clearance of αSyn leading to intracellular pathologies, which then leads to increased microgliosis as a form of non-cell-autonomous response to the escalating proteinopathy. This would be consistent with our earlier report where we found that inflammatory preconditioning of the CNS by Interleukin-6 restricted the seeding and transmission of αSyn in the brain^42^. Such non-cell-autonomous effects of immune cells on neuronal proteinopathy is especially apparent in mouse models of ALS^41^. It is noteworthy that Il-10 itself did not increase microgliosis in the brain or spinal cord or promoted synucleinopathy in the seeded model. Interestingly, neither Il-10 nor vIl-10 triggered astrogliosis in the spinal cord relative to GFP-expressing control mice. This highlights a clear disconnect between how microglia and astrocytes respond to the immune stimulus in different areas of the neuraxis. Such differential proliferation properties of microglia and astrocytes in response to vIl-10 and Il-10 underscores the complex role of immune signaling in neurodegenerative cascades. Future work based on spatial profiling techniques or single-cell-based techniques could clarify how microglial and astrocytic functions are differentially regulated in a spatial–temporal context and contribute to the pathologic trajectory of synucleinopathies.

Given the rich dataset implicating chronic neuroinflammation in PD^11^, our data seem somewhat counter-intuitive. To illustrate this point, our data do not agree with at least two earlier studies where IL-10 showed a beneficial effect in acute toxin models of PD^43,44^. In the first study, human IL-10 infusion was shown to reverse lipopolysaccharide-mediated dopaminergic neurodegeneration^43^, and in the second study, AAV-IL-10 rescued toxin-induced reductions in dopamine levels^44^. Unlike the experimental models used in our study, these acute toxin models of PD used in these previous studies do not capture the gradual physiological process that is inherent in neurodegenerative synucleinopathies—thus, any direct comparisons would necessitate cautious interpretation. These acute toxin models also cause both neurodegeneration and neuroinflammation that are relatively nonspecific in nature in that neither of these models robustly recapitulate PD-type protein aggregation and extranigral pathology. In addition, because of their fast trajectory, these models cannot fully recapitulate the insidious nature of synucleinopathies as observed in our peripherally seeded M83+/− model. Using Il-10 infusion is also ineffective as a therapy, as IL-10 has an extremely short half-life (2.0–4.5 h) that can severely limit the efficacy of the experimental paradigm^45^. Taken together, it would seem that the outcomes of Il-10 signaling in mouse models that recapitulate PD-relevant proteinopathy as reported in our study are physiologically distinct from the acute toxin models of dopaminergic neurodegeneration.

In conclusion, we have revealed an unexpected detrimental outcome following the expression of Il-10 and vIl-10 in mouse models of synucleinopathy. While the adverse effects of Il-10 occurred without exacerbation of αSyn pathology, expression of vIl-10 shortened lifespan via microgliosis, autophagic impairment, and accumulation of αSyn in αSyn-seeded mice. Our data demonstrate that immunosuppressive conditioning of the neuraxis exacerbates the pathologic landscape by altering intracellular protein-quality maintenance in conjunction with dysfunctional microgliosis.

## Methods

### Animals

All animals were treated ethically as per protocols approved by the University of Florida Institutional Animal Care and Use Committee regulatory policies. Animals were maintained in specific pathogen-free conditions on a 12-h circadian cycle with unlimited access to food and water. Experiments were performed using standard blinding protocols of NC3R. Homozygous Line M83+/+ (Fig. 1) and hemizygous Line M83+/− (Figs. 2–7) express the human αSyn (*SNCA*) gene with the A53T mutation under control of the mouse prion protein promoter^13^. Intramuscular (IM) injection of preformed αSyn fibrils in M83+/− mice lead to induction of αSyn inclusions and paralysis within ~4 months of fibril injection^14,15^ (Figs. 2–7). Mice used for all analyses were phenotype-matched for paralysis and moribund condition.

### αSyn fibril preparation and IM injection

Recombinant mouse αSyn was expressed in *E. coli* and purified using size-exclusion and ion-exchange chromatography, as previously described^15^. In total, 5 mg/ml mouse αSyn protein solubilized in sterile PBS (Invitrogen) was incubated at 37 °C with continuous shaking at 1050 rpm. αSyn fibril formation was validated using K114 fluorometry, as previously described^15^. Immediately before injection, mouse αSyn fibrils were diluted to 1 mg/ml in sterile PBS and fragmented by water bath sonication for 1 h^15^. Two-month-old M83 +/− mice were anesthetized with isoflurane. After shaving the back of the hindlimb, a 10-μl Hamilton syringe with a 27-gauge needle was inserted ~1 mm into the gastrocnemius muscle to deliver 5 µg of αSyn fibril or 5 µl of sterile PBS in each hindlimb^15^.

### AAV preparation and injection

GFP and murine Il-10-expressing recombinant AAVs plasmids have been generated previously and described earlier^18,19^. I87A vIl-10 was a kind gift from Dr. Scott Loiler and Dr. Terrence Flotte at the University of Florida. AAV serotype 1 was packaged by methods described earlier^18,19^. Briefly, AAV vectors expressing GFP, Il-10 and, vIl-10 under the control of the cytomegalovirus enhancer/chicken beta-actin (CBA) promoter, a woodchuck hepatitis virus post-transcriptional regulatory element (WPRE), and the bovine growth hormone polyA were transfected in HEK293T cells using linear polyethylenimine (PEI, Polysciences). Cells were co-transfected with the helper plasmid pDP1rs. After 4 days, the packaged virus was purified from the cell lysates using a discontinuous Iodixanol gradient followed by buffer exchange in sterile PBS. The genomic titer was determined by quantitative PCR as described earlier^19^. AAVs were then aliquoted and stored at −80 °C until further use. For neonatal injections, AAV was diluted in sterile 1× DPBS, pH 7.2 to 1E13 vector genomes per ml, and used immediately as described earlier^18^. Briefly, neonatal mice were cryoanesthetized for 3–4 min, resulting in the body temperature being lowered to <10 °C and injected with AAV using 10-μl syringes (1 inch, 33 gauge, 30 degrees beveled needle; Hamilton Company). In total, 1 μl of AAV was slowly injected into the midline, which can be seen as a white line down their back, about 5 mm from the base of the tail. Injected pups were allowed to recover on a heated pad and returned to their home cage.

### Tissue processing and immunohistochemistry

Mice were euthanized with CO_2_ inhalation as per humane conditions and subsequently perfused using ice-cold saline containing heparin. Each spinal cord (cervical, thoracic, and lumbar segments) was divided into three sections—12 mm from the proximal section (containing cervical and thoracic segments, referred to as “thoracic” henceforth), 4 mm from the midline (containing thoracic segment), and 12 mm from the distal section containing lumbar segment (referred to as “lumbar” henceforth). The proximal and distal sections were fixed in 10% normal buffered formalin, while the midline section was flash-frozen for RNA analysis or biochemical analysis. Following paraffin processing, spinal cord fragments representing the thoracic and lumbar sections of each mouse were each serially dissected every ~2 mm into three to four coronal sections and embedded in a paraffin block. Each paraffin block representing the thoracic or lumbar spinal cord was cut at 5 µm thickness and used for immunohistochemistry. The entire brain was fixed in 10% normal buffered formalin. Each brain was then sectioned coronally distanced at 4 mm using a brain matrix and processed for paraffin embedding. Thus, each brain slide contained four coronal sections representative of various anatomical levels of the whole brain. The brain paraffin blocks were sectioned at 10 µm thickness. Paraffin-embedded tissues were immunostained using primary antibodies (Supplementary Table 10) followed by detection with ImmPRESS Polymer Reagents (Vector Laboratories) and visualized using 3,3′-diaminobenzidine (Vector Laboratories). Briefly, slides were deparaffinized, subjected to antigen retrieval, treated with 3% hydrogen peroxide, and blocked in 2% FBS prepared in 0.1 M Tris buffer (pH 7.6) in preparation for primary antibody incubation overnight at 4 °C. Following secondary antibody incubation on the subsequent day, the sections were counterstained with Mayer’s hematoxylin (Sigma), dehydrated, and mounted in Cytoseal 60 (Fisher Scientific). Stained slides were scanned using a whole-slide imager (Aperio ScanScope CS, Leica Biosystems) and images visualized using the Aperio ImageScope software (Leica Biosystems). Quantification of immunostaining was done using the Positive Pixel count program configured to detect brown color (ImageScope, Aperio Technologies). The total number of brown pixels (positive) divided by the total number of pixels (positive+negative) was represented as a % immunoreactivity burden. For the spinal cord, the % immunoreactivity values on all serial sections on each slide were counted for any given sample and averaged for the cohort. For brain sections, the immunoreactivity value from bilateral midbrain or cortex areas were tabulated. For cleaved caspase 3, the total number of positive cells was counted as the staining pattern was sparse. The total number of cells on each slide were counted for any given sample and averaged for the cohort. For co-immunofluorescence staining, the method used was identical as described above, except that slides were not incubated in hydrogen peroxide, Alexa Fluor-conjugated secondary antibodies (1:500; Invitrogen) were used to detect signal, and slides were counterstained and mounted with 4’,6-diamidino-2-phenylindole (DAPI; Southern Biotech). Images were captured using an Olympus BX60 Microscope fitted with an Olympus DP71 camera. Analyses were done on phenotype-matched mice. For the M83+/+ cohort, Il-10-expressing mice typically were 4–6 months old and controls were typically 8–12 months old (Fig. 1), whereas for αSyn-seeded M83+/− cohort, end-stage paralyzed mice typically aged 5–7 months were used (Figs. 2–7).

### Biochemical analysis of the injected spinal cord

Biochemical analysis was done using the thoracic spinal cord segment. The frozen tissue was cryopulverized in liquid nitrogen and divided into aliquots for protein extraction or RNA extraction. The cryopulverized tissue was homogenized in RIPA buffer containing protease and phosphatase inhibitors (Pierce), and homogenates were cleared by centrifugation at 22,000 rpm at 4 °C for 30 min (TLA-55 rotor, Beckman Coulter). Il-10 and vIl-10 levels were analyzed using RIPA-solubilized protein lysates using mouse-specific BD OptiEIA kits (BD Biosciences) as per the manufacturer’s recommendations. GFAP immunoblots were done using RIPA-solubilized lysates using anti-rabbit GFAP antibody (1:500, Dako) as previously described^19^. All blots derive from the same experiment and processed in parallel.

### Quantification of TH neurons in the substantia nigra (SN)

Quantification of TH neurons were done based on a previously published protocol^46^. Dopaminergic neurons stained with the anti-TH polyclonal antibody (Millipore, 1:1000) were counted manually. Serial sections were cut at 10 μm intervals and slides containing the SN were delineated. Every 10th section was used for manual counting, whereby the number of TH neurons in the sections were averaged as representative of this particular area. The final count is a summation from each mouse and then averaged for each cohort.

### Fluoro-Jade C staining

Paraffin-embedded slides were deparaffinized, rehydrated, and incubated at room temperature in 0.06% KMnO_4_ solution for 10 min. Slides were incubated in 0.0001% Fluoro-Jade C (EMD Millipore) dissolved in 0.1% acetic acid for 10 min with gentle shaking. Slides were washed, counterstained with DAPI, and dried on a slide warmer at 50 °C for 5 min. Slides were cleared in xylene for 1 min and then mounted using Cytoseal 60 mounting medium (Fisher Scientific). Slides were imaged using an Olympus BX51 microscope mounted with a DP71 Olympus digital camera. One slide was stained per mouse (each slide contained 2–4 lumbar spinal cord coronal sections). The total number of Fluoro-Jade C-labeled puncta was manually counted for all the spinal cord sections of any one sample and then averaged for each cohort.

### RNA isolation and NanoString analysis

RNA was extracted from cryopulverized aliquots of the thoracic spinal cords obtained from the midline section. For the M83+/+ group, all mice displayed moribund condition (average age of 3–4 months for Il-10 group and 8 months for GFP group). For the preformed αSyn seed-injected M83+/− cohort, the analyses were done on the end-stage paralyzed mice (average age of 5–7 months). RNA was extracted, and 100 ng of the total RNA was analyzed on a custom NanoString codeset as described earlier^19^. Raw count data for each sample was exported from RCC files using nSolver version 4.0. All samples passed QC analysis in nSolver. Count data were imported into R version 3.6 and Bioconductor version 3.9. Count matrices were normalized and differentially expressed genes were analyzed with DESeq2 version 1.24.0 using default normalization parameters and housekeeping genes included in the custom gene panel^47^. For gene ontology analysis, a false discovery rate (FDR) of less than 0.05 was used as a cutoff for significantly changed genes. Goseq version 1.36.0 was used for gene ontology analysis using the list of genes included on the custom NanoString array as background for analysis^48^. The *P* values reported were adjusted for multiple comparisons (*P*adj). Graphs were generated with ggplot2 version 3.2.0. To calculate the M1-, M2-, and DAM-type signature, we used the geometric means of a set of genes previously identified to be increased under these specific conditions (Supplementary Table 2)^20^. Geometric means, rather than arithmetic means, were used so as to reduce the effects of outliers which can skew the values. Briefly, the geometric mean of the counts for the selected genes within each list for each sample was calculated and then within the group, the mean and standard deviation of that value was calculated for the group score followed by a pairwise two-tailed *t* test to test the significance of the association.

### Statistics

For statistical comparisons, we used one-way ANOVA, multiple *t* test with adjustments for multiple comparisons, or two-tailed *t* test depending on the variables tested. Graphical data are plotted as box and whiskers plot, with the whiskers extending from the minimum to maximum values, with the midline representing the median. Graphical representation of data was done using Prism 6 (GraphPad Software, La Jolla, CA), and final images were created using Adobe Photoshop CC (Adobe Systems).

### Reporting summary

Further information on research design is available in the Nature Research Reporting Summary linked to this article.

## Supplementary information

Supplementary Figure and Tables

Reporting Summary

## Data Availability

All data are contained in this paper and Supplementary information files. All materials used to generate the data will be available following the execution of the institutional material transfer agreement.

## Code availability

R Codes will be available on request from the corresponding author.

## References

[1] Tan EK (2020). Parkinson disease and the immune system—associations, mechanisms and therapeutics. Nat. Rev. Neurol..

[2] Doorn KJ (2014). Microglial phenotypes and toll-like receptor 2 in the substantia nigra and hippocampus of incidental Lewy body disease cases and Parkinson’s disease patients. Acta Neuropathol. Commun..

[3] Hamza TH (2010). Common genetic variation in the HLA region is associated with late-onset sporadic Parkinson’s disease. Nat. Genet.

[4] International Parkinson Disease Genomics, C. (2011). Imputation of sequence variants for identification of genetic risks for Parkinson’s disease: a meta-analysis of genome-wide association studies. Lancet.

[5] Nalls MA (2014). Large-scale meta-analysis of genome-wide association data identifies six new risk loci for Parkinson’s disease. Nat. Genet..

[6] Lindestam Arlehamn CS (2020). Alpha-Synuclein-specific T cell reactivity is associated with preclinical and early Parkinson’s disease. Nat. Commun..

[7] Sulzer D (2017). T cells from patients with Parkinson’s disease recognize alpha-synuclein peptides. Nature.

[8] Kim C (2013). Neuron-released oligomeric alpha-synuclein is an endogenous agonist of TLR2 for paracrine activation of microglia. Nat. Commun..

[9] Mao X (2016). Pathological α-synuclein transmission initiated by binding lymphocyte-activation gene 3. Science.

[10] Tang D, Kang R, Coyne CB, Zeh HJ, Lotze MT (2012). PAMPs and DAMPs: signal 0s that spur autophagy and immunity. Immunol. Rev..

[11] Hirsch, E. C. & Standaert, D. G. Ten unsolved questions about neuroinflammation in Parkinson’s Disease. *Movement Disord.*10.1002/mds.28075 (2020). 10.1002/mds.28075 32357266

[12] Ouyang W, Rutz S, Crellin NK, Valdez PA, Hymowitz SG (2011). Regulation and functions of the IL-10 family of cytokines in inflammation and disease. Annu. Rev. Immunol..

[13] Giasson BI (2002). Neuronal alpha-synucleinopathy with severe movement disorder in mice expressing A53T human alpha-synuclein. Neuron.

[14] Sacino AN (2014). Intramuscular injection of alpha-synuclein induces CNS alpha-synuclein pathology and a rapid-onset motor phenotype in transgenic mice. Proc. Natl Acad. Sci. USA.

[15] Sorrentino ZA (2018). Motor neuron loss and neuroinflammation in a model of alpha-synuclein-induced neurodegeneration. Neurobiol. Dis..

[16] Ding Y, Qin L, Kotenko SV, Pestka S, Bromberg JS (2000). A single amino acid determines the immunostimulatory activity of interleukin 10. J. Exp. Med..

[17] Ouyang P (2014). IL-10 encoded by viruses: a remarkable example of independent acquisition of a cellular gene by viruses and its subsequent evolution in the viral genome. J. Gen. Virol..

[18] Ayers JI (2015). Widespread and efficient transduction of spinal cord and brain following neonatal AAV injection and potential disease modifying effect in ALS mice. Mol. Ther..

[19] Chakrabarty P (2015). IL-10 alters immunoproteostasis in APP mice, increasing plaque burden and worsening cognitive behavior. Neuron.

[20] Butovsky O, Weiner HL (2018). Microglial signatures and their role in health and disease. Nat. Rev. Neurosci..

[21] Mosser DM, Zhang X (2008). Interleukin-10: new perspectives on an old cytokine. Immunol. Rev..

[22] Groux H, Cottrez F (2003). The complex role of interleukin-10 in autoimmunity. J. Autoimmun..

[23] Santin AD (2000). Interleukin-10 increases Th1 cytokine production and cytotoxic potential in human papillomavirus-specific CD8(+) cytotoxic T lymphocytes. J. Virol..

[24] Nemani VM (2010). Increased expression of alpha-synuclein reduces neurotransmitter release by inhibiting synaptic vesicle reclustering after endocytosis. Neuron.

[25] Farrer M (2004). Comparison of kindreds with parkinsonism and alpha-synuclein genomic multiplications. Ann. Neurol..

[26] Dhillon JS (2017). A novel panel of alpha-synuclein antibodies reveal distinctive staining profiles in synucleinopathies. PLoS ONE.

[27] Schmued LC, Stowers CC, Scalett AC, Xu L (2005). Fluoro-Jade C results in ultra high resolution and contrast labeling of degenerating neurons. Brain Res..

[28] Park HJ (2011). IL-10 inhibits the starvation induced autophagy in macrophages via class I phosphatidylinositol 3-kinase (PI3K) pathway. Mol. Immunol..

[29] Santarelli R (2014). STAT3 activation by KSHV correlates with IL-10, IL-6 and IL-23 release and an autophagic block in dendritic cells. Sci. Rep..

[30] Deng Z (2017). Autophagy receptors and neurodegenerative diseases. Trends Cell Biol..

[31] Martinet, W., Roth, L. & De Meyer, G. R. Y. Standard immunohistochemical assays to assess autophagy in mammalian tissue. *Cells* **6**, 10.3390/cells6030017 (2017). 10.3390/cells6030017 PMC5617963 28665306

[32] Croisier E, Moran LB, Dexter DT, Pearce RK, Graeber MB (2005). Microglial inflammation in the parkinsonian substantia nigra: relationship to alpha-synuclein deposition. J. Neuroinflamm.

[33] Terada T (2016). Extrastriatal spreading of microglial activation in Parkinson’s disease: a positron emission tomography study. Ann. Nucl. Med..

[34] Iannaccone S (2013). In vivo microglia activation in very early dementia with Lewy bodies, comparison with Parkinson’s disease. Parkinsonism Relat. Dis..

[35] Lindqvist D (2012). Non-motor symptoms in patients with Parkinson’s disease—correlations with inflammatory cytokines in serum. PLoS ONE.

[36] Witoelar A (2017). Genome-wide pleiotropy between Parkinson disease and autoimmune diseases. JAMA Neurol..

[37] Liu Z (2019). IL-17A exacerbates neuroinflammation and neurodegeneration by activating microglia in rodent models of Parkinson’s disease. Brain Behav. Immun..

[38] Gordon, R. et al. Inflammasome inhibition prevents alpha-synuclein pathology and dopaminergic neurodegeneration in mice. *Sci. Transl. Med.* **10**, 10.1126/scitranslmed.aah4066 (2018). 10.1126/scitranslmed.aah4066 PMC6483075 30381407

[39] Naso MF, Tomkowicz B, Perry WL, Strohl WR (2017). Adeno-associated virus (AAV) as a vector for gene therapy. BioDrugs.

[40] Golde TE (2019). Harnessing immunoproteostasis to treat neurodegenerative disorders. Neuron.

[41] Chen H, Kankel MW, Su SC, Han SWS, Ofengeim D (2018). Exploring the genetics and non-cell autonomous mechanisms underlying ALS/FTLD. Cell Death Differ..

[42] Koller EJ, Brooks MM, Golde TE, Giasson BI, Chakrabarty P (2017). Inflammatory pre-conditioning restricts the seeded induction of alpha-synuclein pathology in wild type mice. Mol. Neurodegen..

[43] Arimoto T (2007). Interleukin-10 protects against inflammation-mediated degeneration of dopaminergic neurons in substantia nigra. Neurobiol. Aging.

[44] Joniec-Maciejak I (2014). The influence of AAV2-mediated gene transfer of human IL-10 on neurodegeneration and immune response in a murine model of Parkinson’s disease. Pharm. Rep..

[45] Huhn RD (1997). Pharmacodynamics of subcutaneous recombinant human interleukin-10 in healthy volunteers. Clin. Pharm. Ther..

[46] Kitada T, Tong Y, Gautier CA, Shen J (2009). Absence of nigral degeneration in aged parkin/DJ-1/PINK1 triple knockout mice. J. Neurochem..

[47] Love MI, Huber W, Anders S (2014). Moderated estimation of fold change and dispersion for RNA-seq data with DESeq2. Genome Biol..

[48] Young MD, Wakefield MJ, Smyth GK, Oshlack A (2010). Gene ontology analysis for RNA-seq: accounting for selection bias. Genome Biol..

