# Supplementary material for: Il-10 signaling reduces survival in mouse models of synucleinopathy
Source: NPJ Parkinsons Dis. 2021 Mar 19;7:30. doi: 10.1038/s41531-021-00169-8 (PMC7979923; doi:10.1038/s41531-021-00169-8)
Supplement: Supplementary file 1 — Supplementary Figure and Tables [file 41531_2021_169_MOESM1_ESM.pdf]

**Supplementary Figure Legend and Table Legends.**

**Supplementary Figure 1. Validation of AAV-Il-10 in nontransgenic mice.**

a-c. Neonatal wild type mice were injected with different amounts of AAV-Il-10 into the spinal cord and Il-10 levels were measured in lumbar spinal cord lysate prepared in RIPA buffer (a), CSF (b) and serum (c) of 3 week old mice. The three different doses injected were: High, 10^10 viral genomes; Medium, 0.5x10^10 viral genomes and Low, 10^9 viral genomes. 1-way Anova, **p<0.01; *p<0.05. Nd, not detected. d-f. Expression of Il-10 in the high dose injected mice increased GFAP expression as observed by immunoblotting (d). The values of GFAP protein band were normalized to actin and plotted (e). Representative brain images stained with GFAP are shown (f). 2 tailed unpaired t test; **p<0.01. n=4 mice/group.

**Supplementary Tables 1-10**

**Supplementary Table 1:** Differential gene expression in spinal cord between Il10-injected vs GFP-injected homozygous M83 transgenic mice

**Supplementary Table 2:** List of genes from NanoString codeset used to impute M1-, M2- and DAM-type immune profile.

**Supplementary Table 3.** Differential gene expression in spinal cord between homozygous M83 and non-transgenic mice.

**Supplementary Table 4:** Overlapping gene expression changes in Il-10 expressing homozygous M83 mice vs aged, paralyzed homozygous M83 mice.

**Supplementary Table 5:** Differential gene expression in spinal cord between Il-10 injected vs GFP-injected hemizygous M83 transgenic mice

**Supplementary Table 6:** Differential gene expression in spinal cord between Il-10 injected, α-synuclein seeded vs Il-10 injected, non-seeded (PBS) hemizygous M83 mice

**Supplementary Table 7:** Differential gene expression in spinal cord between Il-10-injected, α-synuclein seeded vs GFP-injected, α-synuclein seeded hemizygous transgenic M83 mice.

**Supplementary Table 8.** Differential gene expression in spinal cord between α-synuclein seeded versus sham-seeded (PBS) hemizygous M83 transgenic mice

**Supplementary Table 9:** Overlapping gene expression changes in Il-10 expressing α-synuclein seeded M83+/- mice vs control α-synuclein seeded M83+/- mice.

**Supplementary Table 10:** List of antibodies used in the study.

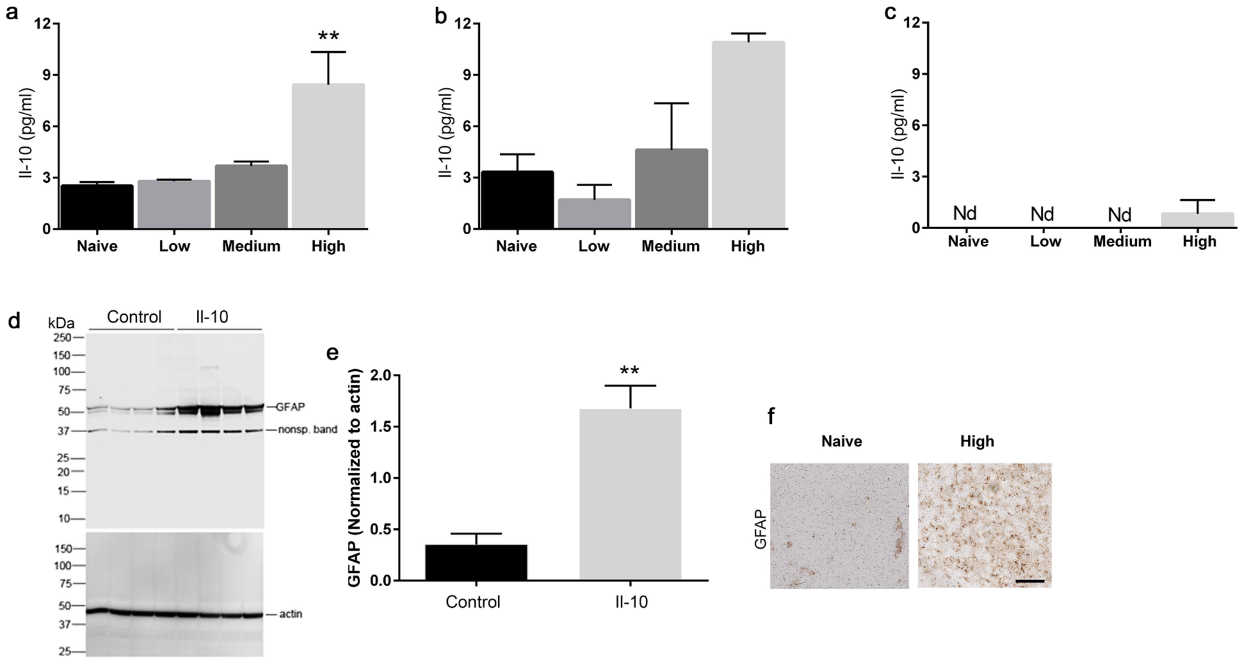


**Supplementary Figure 1. Validation of AAV-Il-10 in nontransgenic mice.**

a-c. Neonatal wild type mice were injected with different amounts of AAV-Il-10 into the spinal cord and Il-10 levels were measured in lumbar spinal cord lysate prepared in RIPA buffer (a), CSF (b) and serum (c) of 3 week old mice. The three different doses injected were: High, 10^10 viral genomes; Medium, 0.5x10^10 viral genomes and Low, 10^9 viral genomes. 1-way Anova, **p<0.01; *p<0.05. Nd, not detected. d-f. Expression of Il-10 in the high dose injected mice increased GFAP expression as observed by immunoblotting (d). The values of GFAP protein band were normalized to actin and plotted (e). Representative brain images stained with GFAP are shown (f). 2 tailed unpaired t test; **p<0.01. n=4 mice/group. 'nonsp.'=nonspecific.

**Supplementary Table 1: Differential gene expression in spinal cord between Il-10 injected vs GFP-injected homozygous M83 transgenic mice**

*Base Mean=gene counts normalized to library depth; lfcSE-log (Fold Change) Standard Error; stat=Wald test statistics*

| | baseMean | log2FoldChange | lfcSE | stat | pvalue | padj |
|---|---|---|---|---|---|---|
| Il10 | 12490.74227 | 13.49551668 | 0.87320897 | 15.45508251 | 6.97E-54 | 1.64E-51 |
| Fcgr2b | 243.5036657 | 2.199739729 | 0.277773436 | 7.919186811 | 2.39E-15 | 2.81E-13 |
| Ccl8 | 41.31312411 | 3.247579667 | 0.721083293 | 4.503751092 | 6.68E-06 | 0.000522988 |
| Fcgr4 | 118.1682517 | 1.785232879 | 0.432490065 | 4.127800899 | 3.66E-05 | 0.002151713 |
| Ms4a6d | 62.8482313 | 1.284898175 | 0.325842477 | 3.943310853 | 8.04E-05 | 0.003777126 |
| Ccl5 | 44.78764641 | 2.47553824 | 0.656390166 | 3.771443216 | 0.000162306 | 0.006356989 |
| Apoe | 8313.289629 | 0.465819669 | 0.125993137 | 3.697182877 | 0.000218005 | 0.007318749 |
| Chil3 | 20.62164335 | 2.799246164 | 0.780006138 | 3.588748894 | 0.000332269 | 0.009760393 |
| Timp1 | 20.74719081 | 2.122012956 | 0.599813707 | 3.5377867 | 0.000403496 | 0.01003646 |
| Abca1 | 205.7215932 | 0.625682925 | 0.177611739 | 3.522756603 | 0.000427083 | 0.01003646 |
| Ptprc | 46.74273194 | 1.293153969 | 0.40498975 | 3.193053571 | 0.001407768 | 0.030075049 |
| Igfbp4 | 117.8473054 | -0.744805204 | 0.24987528 | -2.980707832 | 0.00287583 | 0.056318342 |
| Igfbp5 | 101.6032407 | -0.797798997 | 0.272929342 | -2.923097209 | 0.003465683 | 0.062648885 |
| Cd36 | 40.34741544 | -1.494348257 | 0.519281264 | -2.877724196 | 0.004005552 | 0.067236047 |
| Itgb2 | 61.89588174 | 0.808158199 | 0.296127287 | 2.729090613 | 0.006350925 | 0.099497825 |
| IL6 | 4.433168601 | -2.763456086 | 1.029827726 | -2.683415892 | 0.00728743 | 0.107034132 |
| C5ar1 | 18.23531296 | 1.173156767 | 0.448906724 | 2.613364215 | 0.00896557 | 0.123935823 |
| Fcgr3 | 67.43194505 | 0.634858787 | 0.248195989 | 2.557893017 | 0.010530848 | 0.137486077 |
| Ms4a4a | 13.45679775 | 1.78195407 | 0.7126177 | 2.500575091 | 0.012399184 | 0.149576639 |
| Ccl2 | 31.16525728 | 1.58428251 | 0.635942175 | 2.491236738 | 0.012729927 | 0.149576639 |
| C4a | 645.7671657 | 0.798183858 | 0.322706723 | 2.473403252 | 0.013383304 | 0.149765539 |
| Ctsc | 128.3164067 | 1.021455828 | 0.419595834 | 2.43438029 | 0.014917313 | 0.159344028 |
| P2ry12 | 123.7628662 | -1.104767183 | 0.457249674 | -2.416113661 | 0.015687164 | 0.16028189 |
| Gjc2 | 150.5616757 | -0.871815006 | 0.365295973 | -2.386599004 | 0.017005028 | 0.165125941 |
| Il1b | 10.37484179 | 1.90820501 | 0.803580217 | 2.374629153 | 0.017566589 | 0.165125941 |
| C1qa | 407.390513 | 0.761988136 | 0.328749546 | 2.317837833 | 0.020458136 | 0.180635058 |
| Myl2 | 28.60339256 | -2.722247533 | 1.177222061 | -2.312433333 | 0.020753815 | 0.180635058 |
| C1qb | 498.4449195 | 0.61522738 | 0.269285968 | 2.284661855 | 0.022332663 | 0.181281874 |
| Cfb | 12.0440646 | 1.379296251 | 0.603892438 | 2.284009806 | 0.022370955 | 0.181281874 |
| S100a8 | 361.8696406 | 0.999865711 | 0.440267241 | 2.271042716 | 0.023144392 | 0.181297739 |
| Ece2 | 302.3938394 | -0.408577132 | 0.181794831 | -2.247462861 | 0.024610462 | 0.186563176 |
| Prph | 220.2561612 | -0.849634405 | 0.38385957 | -2.213399044 | 0.026870147 | 0.194030155 |
| Hspb1 | 358.7311702 | 0.738758277 | 0.334588019 | 2.207963929 | 0.027246788 | 0.194030155 |
| Msr1 | 15.81251604 | 1.254410692 | 0.576270976 | 2.176772289 | 0.029497562 | 0.203880205 |
| Trf | 8329.280448 | -0.713467905 | 0.333286804 | -2.140702531 | 0.032298034 | 0.21685823 |
| LRRK2_h | 21.90343444 | -0.881989057 | 0.420195662 | -2.098996101 | 0.035817244 | 0.233807012 |
| Hmox1 | 72.37793693 | -0.496320686 | 0.243227281 | -2.040563395 | 0.041294244 | 0.252994754 |
| Ncf1 | 49.57640442 | 0.600758332 | 0.294889175 | 2.037234267 | 0.041626571 | 0.252994754 |
| Fcer1g | 42.81724101 | 0.890954317 | 0.438104886 | 2.033655285 | 0.041986364 | 0.252994754 |
| Tubb5 | 831.2721472 | -0.286523829 | 0.143399264 | -1.998084374 | 0.045707514 | 0.263821506 |
| Mapt_ex1 | 250.0790438 | -0.372783696 | 0.186846588 | -1.995132477 | 0.046028433 | 0.263821506 |
| Alox5ap | 25.92755227 | 0.802600351 | 0.404968208 | 1.981884837 | 0.047492131 | 0.265729783 |
| Ptpn6 | 43.32816504 | 0.760932029 | 0.392417236 | 1.939089212 | 0.052490476 | 0.286866555 |
| Gusb | 71.26381719 | 0.514487246 | 0.268860172 | 1.913586689 | 0.055672987 | 0.297344363 |
| Sox10 | 171.4381861 | -0.373129818 | 0.198431749 | -1.880393739 | 0.060054436 | 0.312642752 |
| Cat | 502.207505 | -0.376808723 | 0.201280145 | -1.872061065 | 0.061198156 | 0.312642752 |
| Maff | 16.24422212 | 1.068068458 | 0.575977843 | 1.85435685 | 0.063688124 | 0.318440619 |
| Grn | 329.85229 | 0.362809017 | 0.196690903 | 1.844564297 | 0.065100945 | 0.318723375 |
| Crhr1 | 33.38100618 | -0.635426872 | 0.357551294 | -1.777162839 | 0.075541452 | 0.362290636 |
| Dnm1l | 525.2029786 | -0.248125647 | 0.141399008 | -1.754790579 | 0.079295136 | 0.372687138 |
| C1ra | 48.4228978 | 0.811221395 | 0.471624067 | 1.72005937 | 0.08542165 | 0.390352738 |
| Serpina3n | 467.8558077 | 0.396328775 | 0.231729519 | 1.710307674 | 0.087208994 | 0.390352738 |
| Cd14 | 18.46103212 | 0.740008784 | 0.433807831 | 1.705844687 | 0.088037 | 0.390352738 |
| Mef2c | 95.25050685 | -0.378854594 | 0.225593733 | -1.679366661 | 0.093080607 | 0.40507301 |
| Tnfsf9 | 7.122622711 | 1.337268045 | 0.802493372 | 1.66639139 | 0.095635485 | 0.408624343 |
| Tgfbr1 | 159.3850787 | -0.317386406 | 0.191806211 | -1.654724337 | 0.097980428 | 0.411167868 |
| Th | 10.44275149 | -0.990585589 | 0.603674302 | -1.640927211 | 0.100812526 | 0.415630588 |
| Ccl19 | 32.09534692 | 0.609982563 | 0.381732961 | 1.597930031 | 0.11005855 | 0.445926882 |
| Myc | 25.38263129 | 0.566354696 | 0.361303992 | 1.567529583 | 0.116990957 | 0.458558384 |
| Creb1 | 105.6138777 | -0.474874164 | 0.303016942 | -1.567153843 | 0.117078736 | 0.458558384 |
| Fcrls | 42.5241686 | 0.727724248 | 0.469164059 | 1.551108262 | 0.120875741 | 0.46566884 |
| Flii | 224.9672853 | -0.253613256 | 0.168114423 | -1.508575236 | 0.131407367 | 0.488562681 |

| | | | | | | |
|---|---|---|---|---|---|---|
| Mme | 71.61838902 | -0.436180128 | 0.289139178 | -1.508547303 | 0.13141451 | 0.488562681 |
| Scna | 937.4981845 | -0.226434293 | 0.150738966 | -1.502161642 | 0.133055368 | 0.488562681 |
| Mmp3 | 3.655021446 | -1.299522013 | 0.879953186 | -1.476808124 | 0.139727078 | 0.498144421 |
| Lrp1 | 589.7526722 | 0.195956608 | 0.133020328 | 1.473132798 | 0.140715231 | 0.498144421 |
| Mrc1 | 65.65435182 | 0.512334967 | 0.348931941 | 1.468294833 | 0.142024154 | 0.498144421 |
| Serping1 | 56.3437624 | 0.544249295 | 0.37353988 | 1.457004523 | 0.145115132 | 0.501500823 |
| Ccr4 | 11.61389963 | -0.874346114 | 0.604843146 | -1.445574972 | 0.148296451 | 0.505067622 |
| Zbp1 | 29.92884353 | 1.093065555 | 0.771231345 | 1.417299183 | 0.156395474 | 0.525041949 |
| SCNA_h | 31074.72019 | -0.167789201 | 0.121206384 | -1.384326431 | 0.166258524 | 0.550292299 |
| C3ar1 | 43.37187021 | 0.53921199 | 0.398054292 | 1.354619208 | 0.17553891 | 0.566720917 |
| Ppp2ca | 1838.479322 | -0.159771243 | 0.11810614 | -1.352776772 | 0.176126956 | 0.566720917 |
| C2 | 56.03953294 | 0.64244556 | 0.477469469 | 1.345521759 | 0.178456799 | 0.566720917 |
| Adam10 | 454.7865363 | -0.216458556 | 0.162313138 | -1.333586173 | 0.182339517 | 0.56847721 |
| Tubb3 | 1339.118136 | -0.329920992 | 0.248247537 | -1.329000064 | 0.183847949 | 0.56847721 |
| Mapt_e9 | 42.98489992 | -0.382559868 | 0.292083691 | -1.30976114 | 0.190276653 | 0.574938026 |
| Ftl | 546.8371831 | -0.268096806 | 0.204947196 | -1.308126243 | 0.190830494 | 0.574938026 |
| Cebpd | 13.41962529 | 0.809282236 | 0.634218351 | 1.276030936 | 0.201944584 | 0.60072123 |
| App | 1971.041157 | -0.174506645 | 0.138463762 | -1.260305532 | 0.207559165 | 0.603044817 |
| Npy | 139.0644673 | -0.40438262 | 0.321071793 | -1.259477255 | 0.207858001 | 0.603044817 |
| Cd55 | 126.5633909 | -0.69395008 | 0.554706941 | -1.251021087 | 0.210926784 | 0.603972531 |
| Igfbp3 | 212.4392228 | 0.500756441 | 0.402377678 | 1.244493588 | 0.213317958 | 0.603972531 |
| FTL_h | 5.38572549 | -0.90454014 | 0.738198098 | -1.225335236 | 0.220448938 | 0.616732147 |
| Mertk | 77.92042948 | 0.398357961 | 0.328121466 | 1.214056386 | 0.224726193 | 0.621301828 |
| Mmp2 | 43.58141005 | -0.369752092 | 0.311845919 | -1.18568841 | 0.235745373 | 0.642126647 |
| APP_h | 3156.880733 | -0.153058249 | 0.12963391 | -1.180696073 | 0.237723482 | 0.642126647 |
| Cd68 | 40.64837332 | 0.488367292 | 0.41890775 | 1.165811068 | 0.243690841 | 0.650140857 |
| Nlrp3 | 10.5859416 | -0.725724526 | 0.62585611 | -1.15957089 | 0.246223559 | 0.650140857 |
| Il1rn | 6.485408147 | 0.789725003 | 0.693503276 | 1.138747329 | 0.254808556 | 0.65087786 |
| Ccl11 | 9.681112864 | -0.791984601 | 0.705900316 | -1.121949633 | 0.261883856 | 0.65087786 |
| H2Eb1 | 47.39582465 | 0.659638787 | 0.588209259 | 1.121435573 | 0.262102501 | 0.65087786 |
| IL10rb | 106.5379911 | -0.235445986 | 0.211297712 | -1.114285547 | 0.265156706 | 0.65087786 |
| P2ry6 | 34.52673845 | 0.367088935 | 0.329913222 | 1.112683307 | 0.265844467 | 0.65087786 |
| Tnf | 2.623088044 | -1.212914746 | 1.091973555 | -1.110754689 | 0.266673955 | NA |
| Treml2 | 6.59670853 | 0.944296464 | 0.861809669 | 1.095713472 | 0.273204185 | 0.65087786 |
| Casp8 | 35.85306863 | 0.389255261 | 0.355553683 | 1.094786188 | 0.273610319 | 0.65087786 |
| Cxcl9 | 76.91839919 | 0.749720071 | 0.690960465 | 1.085040475 | 0.277903729 | 0.65087786 |
| Plat | 169.6599912 | 0.217977765 | 0.202042612 | 1.078870259 | 0.280645569 | 0.65087786 |
| Treml1 | 2.745689612 | 1.099248538 | 1.018928995 | 1.07882742 | 0.280664669 | NA |
| Ide | 185.0938011 | -0.228319155 | 0.212874774 | -1.072551485 | 0.283472402 | 0.65087786 |
| B2m | 2778.128103 | 0.495105716 | 0.462736191 | 1.069952438 | 0.284640718 | 0.65087786 |
| Crh | 32.47894871 | -0.489278499 | 0.458288476 | -1.067621213 | 0.285691411 | 0.65087786 |
| Map2k6 | 42.94567021 | 0.326646524 | 0.309381405 | 1.05580529 | 0.291057181 | 0.65087786 |
| Egr1 | 52.04247795 | -0.416402395 | 0.395711034 | -1.052289069 | 0.292666945 | 0.65087786 |
| Cd33 | 16.08607508 | 0.529709406 | 0.503863626 | 1.051295189 | 0.293123036 | 0.65087786 |
| Itgax | 3.729260537 | -0.899105832 | 0.855257958 | -1.051268595 | 0.293135246 | 0.65087786 |
| Itgam | 58.7643004 | 0.516533101 | 0.498996148 | 1.035144466 | 0.300601449 | 0.65087786 |
| Cfl1 | 1681.24713 | -0.134058483 | 0.130066564 | -1.030691355 | 0.30268558 | 0.65087786 |
| Tnfsf1a | 103.4842309 | 0.238121712 | 0.231678584 | 1.027810634 | 0.304038913 | 0.65087786 |
| Card9 | 36.04870983 | 0.336494512 | 0.32752366 | 1.027389938 | 0.304236888 | 0.65087786 |
| Il12a | 5.16988072 | 0.873052414 | 0.854414384 | 1.021813807 | 0.306869033 | 0.65087786 |
| Igf1 | 45.55120612 | -0.357930263 | 0.352677541 | -1.014893839 | 0.310156435 | 0.65087786 |
| Pgk1 | 3035.80998 | -0.141321707 | 0.139446883 | -1.01344472 | 0.310847788 | 0.65087786 |
| Bace1 | 365.3799489 | -0.150465967 | 0.149124061 | -1.008998588 | 0.312975312 | 0.65087786 |
| Gapdh | 8400.488752 | -0.11189469 | 0.112937523 | -0.990766283 | 0.321799717 | 0.662580346 |
| GRN_h | 3.559611368 | 1.037869271 | 1.052841457 | 0.985779258 | 0.324241446 | 0.662580346 |
| Trem1 | 21.84435414 | -0.456399264 | 0.471235155 | -0.968517011 | 0.332786227 | 0.66847389 |
| Eif2s1 | 207.6531903 | -0.165179058 | 0.171091098 | -0.965445073 | 0.334321935 | 0.66847389 |
| Vwf | 135.0111334 | -0.301833205 | 0.313502577 | -0.962777429 | 0.33565923 | 0.66847389 |
| S100a9 | 54.53827016 | 0.422758622 | 0.44496088 | 0.950102899 | 0.342059971 | 0.670245183 |
| Mafk | 30.69890456 | 0.402396292 | 0.425486087 | 0.945733137 | 0.344284716 | 0.670245183 |
| Casp1 | 28.1494098 | 0.371623936 | 0.396165148 | 0.938053077 | 0.348217134 | 0.670245183 |
| Tardbp | 437.6973295 | -0.147183237 | 0.157746087 | -0.93303891 | 0.350799879 | 0.670245183 |
| Ece1 | 101.1018265 | -0.216120652 | 0.231635383 | -0.933020893 | 0.350809181 | 0.670245183 |
| H2-Ea-ps | 156.9035967 | -0.756187153 | 0.819737969 | -0.922474232 | 0.356281265 | 0.675210463 |
| Pink1 | 698.3537904 | 0.14605467 | 0.168034707 | 0.869193469 | 0.384741319 | 0.72331368 |

| | | | | | | |
|---|---|---|---|---|---|---|
| Ptger4 | 13.97580854 | 0.475969773 | 0.567336773 | 0.838954561 | 0.401494808 | 0.744097957 |
| Tgfb3 | 66.89987424 | -0.222563322 | 0.267614855 | -0.831655335 | 0.405603519 | 0.744097957 |
| Cx3cr1 | 124.0992195 | -0.580973494 | 0.701816226 | -0.827814281 | 0.407775686 | 0.744097957 |
| Gba | 110.9234987 | -0.172000947 | 0.208924657 | -0.823267821 | 0.410355708 | 0.744097957 |
| Tnfsf11 | 19.71552873 | -0.501829755 | 0.611219071 | -0.821030918 | 0.411628657 | 0.744097957 |
| Ccr1 | 32.40356603 | 0.294237823 | 0.361934933 | 0.81295779 | 0.41624226 | 0.74669413 |
| Sod1 | 2899.765646 | -0.09939919 | 0.12422645 | -0.800145142 | 0.423626709 | 0.751983916 |
| Mmp9 | 15.97755784 | 0.357672285 | 0.449038555 | 0.796529119 | 0.425724558 | 0.751983916 |
| Calb | 154.1059021 | -0.26490337 | 0.335475908 | -0.789634558 | 0.42974122 | 0.751983916 |
| Il7 | 9.570324492 | -0.498270529 | 0.63410171 | -0.785789601 | 0.43199076 | 0.751983916 |
| Ptger1 | 3.826649087 | 0.728380576 | 0.940059576 | 0.774823846 | 0.438443757 | 0.757605021 |
| Fcgr1 | 45.71263632 | 0.285952978 | 0.381177033 | 0.750184175 | 0.453143788 | 0.773399261 |
| Ppp1r2 | 471.3484572 | -0.129090788 | 0.172468944 | -0.748487148 | 0.454166374 | 0.773399261 |
| TARDBP_h | 3.1420944 | 0.831956486 | 1.134751229 | 0.733162005 | 0.463459626 | 0.782286273 |
| Ptgs2 | 27.14829829 | -0.341091743 | 0.467933165 | -0.72893261 | 0.466042886 | 0.782286273 |
| Tlr8 | 3.06850258 | -0.694523119 | 0.966436707 | -0.71864315 | 0.47236082 | 0.787268033 |
| Gls | 583.883255 | -0.113920942 | 0.161178027 | -0.706801936 | 0.479689571 | 0.793664691 |
| Csf1 | 152.3433474 | -0.129743028 | 0.185981575 | -0.697612265 | 0.485419709 | 0.793664691 |
| Mmp11 | 17.17951772 | -0.343248632 | 0.493062316 | -0.696156695 | 0.486330704 | 0.793664691 |
| Tyrobp | 269.0420835 | 0.220530661 | 0.321006584 | 0.686997314 | 0.492084421 | 0.797516131 |
| Crp | 2.839884083 | -0.671668898 | 0.982591056 | -0.683569114 | 0.494247293 | NA |
| Entpd1 | 54.96156414 | -0.196678712 | 0.293834314 | -0.669352431 | 0.503270688 | 0.798074519 |
| Bsn | 95.64058619 | 0.267992698 | 0.401253343 | 0.66788901 | 0.504204443 | 0.798074519 |
| Ccl4 | 20.84590255 | -0.386797109 | 0.580852983 | -0.665912237 | 0.505467199 | 0.798074519 |
| Il1a | 4.611776947 | 0.596035865 | 0.907151357 | 0.657041254 | 0.511154391 | 0.798074519 |
| Aqp4 | 949.8680406 | -0.14662841 | 0.223280883 | -0.656699347 | 0.511374255 | 0.798074519 |
| Tlr9 | 9.217495408 | 0.409233197 | 0.625927705 | 0.653802658 | 0.513238954 | 0.798074519 |
| Cxcl10 | 36.09345709 | 0.430268796 | 0.662755512 | 0.649211946 | 0.516201391 | 0.798074519 |
| Treml4 | 17.66862837 | -0.378086072 | 0.594778723 | -0.635675179 | 0.524988157 | 0.802503373 |
| Ccl3 | 20.28092565 | 0.358009117 | 0.574202301 | 0.62348952 | 0.532962887 | 0.802503373 |
| Fos | 39.82746028 | 0.201928956 | 0.324747409 | 0.621803133 | 0.534071326 | 0.802503373 |
| Tnfrsf1b | 6.524483684 | 0.424430852 | 0.685635391 | 0.619032881 | 0.535894698 | 0.802503373 |
| Hspb2 | 19.811502 | -0.241571722 | 0.394409266 | -0.612489977 | 0.540213628 | 0.802503373 |
| Il1r1 | 54.65914365 | 0.204363552 | 0.334977771 | 0.610080935 | 0.541808195 | 0.802503373 |
| Arc | 22.31475034 | -0.29201309 | 0.48177095 | -0.606124321 | 0.544432202 | 0.802503373 |
| Ly96 | 38.21870869 | 0.234971992 | 0.389551774 | 0.603185527 | 0.546385275 | 0.802503373 |
| Clu | 5298.302556 | -0.100976661 | 0.170561197 | -0.592025988 | 0.553833186 | 0.806070747 |
| Mapk8 | 284.2163151 | -0.128663791 | 0.218341137 | -0.589278743 | 0.555674302 | 0.806070747 |
| Adam17 | 124.4796425 | 0.123894756 | 0.212542822 | 0.582916679 | 0.559949392 | 0.807289001 |
| Tgfb1 | 34.01027417 | 0.202149948 | 0.355232193 | 0.569064268 | 0.569312526 | 0.810435451 |
| Ccnd1 | 42.8871477 | -0.252585847 | 0.44420011 | -0.568630761 | 0.569606745 | 0.810435451 |
| Tlr6 | 21.01319729 | 0.246725453 | 0.437141741 | 0.564406072 | 0.572477808 | 0.810435451 |
| Cd163 | 28.08037039 | 0.225788678 | 0.411347033 | 0.548900709 | 0.583073591 | 0.819558173 |
| Cxcr2 | 20.42140123 | -0.218584785 | 0.41706886 | -0.524097591 | 0.600210664 | 0.819558173 |
| Stat6 | 67.55556278 | 0.153292721 | 0.292557336 | 0.523974968 | 0.60029595 | 0.819558173 |
| Dock2 | 10.72704255 | -0.312970953 | 0.603855179 | -0.518288099 | 0.604257275 | 0.819558173 |
| Tlr1 | 9.284370372 | 0.359171321 | 0.695100821 | 0.51671831 | 0.605352813 | 0.819558173 |
| Stat3 | 341.4660603 | 0.097373297 | 0.188867858 | 0.51556309 | 0.606159595 | 0.819558173 |
| Plg | 16.5663441 | -0.278818745 | 0.541515352 | -0.514886132 | 0.606632592 | 0.819558173 |
| Mpo | 42.41776707 | 0.306012412 | 0.594642927 | 0.514615407 | 0.606821796 | 0.819558173 |
| Mef2b | 3.512013142 | 0.43924238 | 0.876818105 | 0.500950399 | 0.616406031 | 0.827745242 |
| Cltc | 3631.218697 | -0.057770852 | 0.118473366 | -0.487627333 | 0.625813832 | 0.834663877 |
| Syp | 811.2302683 | -0.086522361 | 0.178908877 | -0.483611335 | 0.628661729 | 0.834663877 |
| Csf1r | 130.3622671 | -0.157716752 | 0.331318164 | -0.476028086 | 0.634054369 | 0.837094251 |
| Grin2a | 82.85197237 | -0.101817181 | 0.22609408 | -0.450331035 | 0.652471765 | 0.856597009 |
| Jak3 | 18.71362119 | 0.189921094 | 0.433423937 | 0.438187828 | 0.661250131 | 0.859541941 |
| Cxcr4 | 34.87868155 | 0.243682948 | 0.565822216 | 0.430670519 | 0.666707958 | 0.859541941 |
| Ctsd | 2774.889069 | 0.071740194 | 0.169353297 | 0.423612621 | 0.67184835 | 0.859541941 |
| Atp13a2 | 38.8490069 | -0.143118886 | 0.338040946 | -0.423377366 | 0.672019956 | 0.859541941 |
| Mfn1 | 192.7199935 | 0.083542333 | 0.199877175 | 0.417968349 | 0.675970259 | 0.859541941 |
| MAPT_h_ex1 | 6.053942943 | 0.385789418 | 0.925100702 | 0.417024241 | 0.676660677 | 0.859541941 |
| Cxcl15 | 3.040577238 | -0.451054183 | 1.110746085 | -0.406082173 | 0.684682224 | NA |
| Gfap | 1818.02684 | -0.090939561 | 0.225115365 | -0.403968697 | 0.686235738 | 0.867018271 |
| Hck | 11.02257035 | 0.256688371 | 0.655736098 | 0.391450726 | 0.695464104 | 0.873978954 |
| Tlr2 | 13.89391611 | 0.17478767 | 0.465340375 | 0.375612517 | 0.707204984 | 0.877498331 |

| | | | | | | |
|---|---|---|---|---|---|---|
| IL6ra | 47.37062545 | 0.106343047 | 0.284750873 | 0.373459951 | 0.708806147 | 0.877498331 |
| Fcgrt | 128.6882536 | -0.092779206 | 0.249795601 | -0.371420498 | 0.71032436 | 0.877498331 |
| ITM2b_h | 3.86703571 | 0.365841365 | 1.006054829 | 0.36363959 | 0.71612716 | 0.877498331 |
| Dlg4 | 303.9755288 | -0.056955669 | 0.15838677 | -0.359598655 | 0.71914729 | 0.877498331 |
| Marco | 25.32121385 | 0.198256094 | 0.556990039 | 0.355941902 | 0.721884075 | 0.877498331 |
| Map2k4 | 943.1683369 | 0.047382502 | 0.135455861 | 0.349800312 | 0.726488565 | 0.877498331 |
| Ly86 | 192.7761376 | 0.196881509 | 0.566394489 | 0.347604917 | 0.728136913 | 0.877498331 |
| Serpina1a | 350.1069493 | -0.137165038 | 0.412112512 | -0.332833956 | 0.739259625 | 0.886357204 |
| Ccl7 | 25.22467325 | -0.127542429 | 0.397238237 | -0.321072891 | 0.748155153 | 0.886838886 |
| Ptgds | 12310.12107 | 0.105371013 | 0.335900151 | 0.313697427 | 0.753750856 | 0.886838886 |
| Snap91 | 831.2436929 | -0.049029926 | 0.159487631 | -0.307421496 | 0.758522568 | 0.886838886 |
| Ubc | 1179.009162 | 0.052725681 | 0.172290372 | 0.306028016 | 0.759583315 | 0.886838886 |
| Itm2b | 3558.995834 | -0.0459977 | 0.151806903 | -0.303001374 | 0.761888814 | 0.886838886 |
| Lrrk2 | 56.7949121 | -0.097249191 | 0.323248496 | -0.300849632 | 0.76352916 | 0.886838886 |
| Cxcl5 | 30.11348958 | -0.178935062 | 0.601473681 | -0.297494416 | 0.76608907 | 0.886838886 |
| Igfbp2 | 211.1120549 | -0.117689545 | 0.414098302 | -0.284206781 | 0.776251919 | 0.886838886 |
| C1s1 | 15.45931097 | 0.231172584 | 0.823146543 | 0.280840132 | 0.778833021 | 0.886838886 |
| Bdnf | 25.88418457 | -0.125470357 | 0.448522692 | -0.279741381 | 0.779675928 | 0.886838886 |
| Ace | 82.81591093 | 0.090915514 | 0.327280542 | 0.277790771 | 0.781172976 | 0.886838886 |
| Aifm1 | 182.0824006 | 0.05103999 | 0.187337032 | 0.272450083 | 0.785275968 | 0.887210829 |
| Tlr7 | 32.95475786 | 0.137459038 | 0.551324963 | 0.249324894 | 0.803109477 | 0.903017833 |
| Ctsb | 190.0199941 | -0.042459659 | 0.184727989 | -0.229849624 | 0.818208622 | 0.908676245 |
| Hprt1 | 1596.964125 | -0.031029372 | 0.138737738 | -0.22365488 | 0.823025857 | 0.908676245 |
| Masp2 | 3.453505113 | 0.219832051 | 0.985726719 | 0.223015209 | 0.823523669 | 0.908676245 |
| SOD1_h | 4.325624518 | 0.231401332 | 1.038111817 | 0.22290598 | 0.823608682 | 0.908676245 |
| Arg1 | 23.14783947 | 0.116826315 | 0.560146613 | 0.208563816 | 0.83478876 | 0.916707283 |
| APOE_h | 3.759498009 | -0.182230833 | 0.924643156 | -0.197082336 | 0.843763106 | 0.922252697 |
| Ptafr | 14.7536887 | 0.085747074 | 0.461222122 | 0.185912751 | 0.852513183 | 0.927502768 |
| Ccl17 | 13.34300592 | 0.092647563 | 0.656195107 | 0.141189049 | 0.887720597 | 0.957138898 |
| C3 | 145.0121541 | -0.061590986 | 0.453679062 | -0.135758935 | 0.892011856 | 0.957138898 |
| Tlr4 | 23.29699242 | 0.047485439 | 0.360362691 | 0.131771242 | 0.895165234 | 0.957138898 |
| Trem2 | 60.46664707 | 0.043407286 | 0.332702646 | 0.130468714 | 0.896195605 | 0.957138898 |
| Ifih1 | 29.87111195 | -0.041594062 | 0.331393876 | -0.125512465 | 0.900117857 | 0.957138898 |
| MAPT_h_ex10 | 6.274858549 | -0.078287549 | 0.675773275 | -0.115848838 | 0.907772344 | 0.960930184 |
| Ccl21b | 3.760918574 | 0.101280844 | 0.919955398 | 0.110093211 | 0.912335452 | 0.961429737 |
| Ifng | 5.062862528 | 0.07775592 | 0.767990602 | 0.101245926 | 0.91935524 | 0.96450215 |
| Ackr3 | 34.43441439 | 0.03085824 | 0.329130151 | 0.093756951 | 0.925302229 | 0.966426772 |
| Axin1 | 33.18670191 | -0.025310557 | 0.393559374 | -0.064311914 | 0.948721867 | 0.982193232 |
| Lyz2 | 129.3367445 | 0.011407351 | 0.188953737 | 0.06037113 | 0.951860052 | 0.982193232 |
| Alox5 | 18.57257417 | 0.030351307 | 0.521338152 | 0.058218082 | 0.953574918 | 0.982193232 |
| Prkcb | 421.6214797 | 0.011262478 | 0.209444544 | 0.05377308 | 0.957115958 | 0.982193232 |
| Ltbr | 42.88683041 | 0.015364202 | 0.351306458 | 0.043734471 | 0.965116061 | 0.985763162 |
| S100a11 | 337.6206824 | 0.011190521 | 0.287804893 | 0.038882317 | 0.968984215 | 0.985763162 |
| Ubqln1 | 105.0272715 | 0.004343134 | 0.220431879 | 0.01970284 | 0.984280425 | 0.99109069 |
| Limk1 | 289.6805036 | 0.004536493 | 0.239019508 | 0.018979591 | 0.984857387 | 0.99109069 |
| Mbl2 | 2.280481042 | 0.022326652 | 1.271579773 | 0.0175582 | 0.985991303 | NA |
| Nos2 | 3.369637547 | -0.010968419 | 0.916934088 | -0.011962059 | 0.990455886 | 0.99109069 |
| Il4 | 16.80863532 | 0.008378184 | 0.750303324 | 0.011166396 | 0.99109069 | 0.99109069 |

**Supplementary Table 2: List of genes from NanoString codeset used to impute M1-, M2- and DAM-type immune profile**

| M1 signature | M2 signature | DAM signature |
|---|---|---|
| Ccl2 | Arg1 | Apoe |
| Ccl3 | Chil3 (Ym1) | B2m |
| Cd68 | Il10 | Csf1 |
| Ifng | IL4 | Ctsb |
| Il1b | Tgfb1 | Ctsd |
| IL6 | Tgfb3 | Itgax |
| Nos2 | | Lyz2 |
| Tnf | | Trem2 |
| | | Tyrobp |

**Supplementary Table 3: Differential gene expression in spinal cord between homozygous M83 and non-transgenic mice**

*Base Mean=gene counts normalized to library depth; lfcSE-log (Fold Change) Standard Error; stat=Wald test statistics*

| | baseMean | log2FoldChange | lfcSE | stat | pvalue | padj |
|---|---|---|---|---|---|---|
| SCNA_h | 16832.80687 | 8.170713759 | 0.145300779 | 56.23310373 | 0 | 0 |
| Scna | 551.9144861 | 3.311700513 | 0.178576628 | 18.54498291 | 8.95E-77 | 9.89E-75 |
| Serpina1a | 191.7085641 | 4.943368854 | 0.455253776 | 10.85849062 | 1.82E-27 | 1.34E-25 |
| SOD1_h | 67.67994199 | -5.736938639 | 0.561502031 | -10.21712892 | 1.66E-24 | 9.18E-23 |
| Ptgds | 7177.22474 | 1.85848357 | 0.184525363 | 10.07169711 | 7.37E-24 | 3.26E-22 |
| Gjc2 | 315.1958931 | -1.689718258 | 0.193311775 | -8.740896711 | 2.31E-18 | 8.52E-17 |
| Limk1 | 368.3116851 | -1.173682407 | 0.146565573 | -8.007899674 | 1.17E-15 | 3.68E-14 |
| H2-Ea-ps | 101.29495 | 6.694603698 | 0.901391989 | 7.426961606 | 1.11E-13 | 3.07E-12 |
| S100a8 | 129.9976343 | 3.497464848 | 0.512861196 | 6.819515453 | 9.13E-12 | 2.24E-10 |
| Serpina3n | 247.4349268 | 1.741805235 | 0.260016583 | 6.698823643 | 2.10E-11 | 4.64E-10 |
| Ftl | 401.3877729 | 1.093688716 | 0.170935508 | 6.398253531 | 1.57E-10 | 3.16E-09 |
| Ubc | 1408.131298 | -1.054696379 | 0.177635306 | -5.937425418 | 2.90E-09 | 5.33E-08 |
| Pink1 | 746.5777756 | -0.855546045 | 0.149255916 | -5.73207461 | 9.92E-09 | 1.69E-07 |
| S100a11 | 216.5307944 | 1.374243445 | 0.243609829 | 5.641165842 | 1.69E-08 | 2.67E-07 |
| App | 2196.89874 | -0.666620021 | 0.12294915 | -5.421916477 | 5.90E-08 | 8.69E-07 |
| APP_h | 3512.307587 | -0.680107508 | 0.128570177 | -5.289776571 | 1.22E-07 | 1.69E-06 |
| Snap91 | 896.7041629 | -0.697035259 | 0.134202507 | -5.193906393 | 2.06E-07 | 2.68E-06 |
| Dlg4 | 360.650091 | -0.945728148 | 0.184000158 | -5.139822467 | 2.75E-07 | 3.38E-06 |
| Lyz2 | 81.29337046 | 1.508629587 | 0.297990705 | 5.062673311 | 4.13E-07 | 4.81E-06 |
| Igfbp3 | 107.4077908 | 1.76228994 | 0.355887162 | 4.951822171 | 7.35E-07 | 8.12E-06 |
| Ppp2ca | 2001.150337 | -0.611912119 | 0.125964623 | -4.857809315 | 1.19E-06 | 1.25E-05 |
| Cltc | 3759.102924 | -0.56462451 | 0.118099895 | -4.780906104 | 1.75E-06 | 1.75E-05 |
| C2 | 24.64643092 | 2.936644198 | 0.640411517 | 4.585558067 | 4.53E-06 | 4.35E-05 |
| Mrc1 | 30.43328613 | 2.805655128 | 0.626787125 | 4.47624882 | 7.60E-06 | 7.00E-05 |
| Serping1 | 25.58632651 | 2.868982747 | 0.647355854 | 4.431848004 | 9.34E-06 | 8.26E-05 |
| Trf | 10474.74524 | -0.55668934 | 0.128357314 | -4.337028584 | 1.44E-05 | 0.000122759 |
| Lrp1 | 566.2657238 | -0.611886325 | 0.145409323 | -4.208026779 | 2.58E-05 | 0.000210859 |
| Bace1 | 404.8843732 | -0.667537044 | 0.161986443 | -4.120943889 | 3.77E-05 | 0.000297816 |
| Mapt_ex1 | 303.057969 | -0.716139833 | 0.175595654 | -4.078345998 | 4.54E-05 | 0.000345653 |
| Hmox1 | 53.74427778 | 1.470708954 | 0.365333574 | 4.025660541 | 5.68E-05 | 0.000418542 |
| Igf1 | 30.31122807 | 2.073412297 | 0.521759848 | 3.973882438 | 7.07E-05 | 0.000504097 |
| Fcgr2b | 57.11601421 | 1.238220275 | 0.338150694 | 3.661741041 | 0.000250507 | 0.001679516 |
| Pgk1 | 3084.569401 | -0.424728559 | 0.115999958 | -3.661454423 | 0.000250787 | 0.001679516 |
| C3 | 173.3493866 | -0.938332168 | 0.261272569 | -3.591391827 | 0.000328917 | 0.002137959 |
| Bsn | 101.0299229 | -0.974105636 | 0.275590719 | -3.534609725 | 0.000408378 | 0.002578614 |
| Ece1 | 122.3386623 | -0.830309743 | 0.236126738 | -3.516373247 | 0.000437485 | 0.002685675 |
| Syp | 816.2057005 | -0.461125556 | 0.135012786 | -3.415421372 | 0.000636834 | 0.003803793 |
| Tubb3 | 1493.228507 | -0.525683811 | 0.154690623 | -3.398291382 | 0.000678082 | 0.00394358 |
| Ubqln1 | 116.5749302 | -0.819487459 | 0.246546029 | -3.323872063 | 0.000887769 | 0.005030692 |
| S100a9 | 27.60991217 | 1.988652863 | 0.605807287 | 3.282649292 | 0.001028365 | 0.005681718 |
| Ece2 | 342.6790774 | -0.496098199 | 0.153537447 | -3.231121838 | 0.001233054 | 0.00664646 |
| Map2k4 | 895.8558269 | -0.421047043 | 0.130970835 | -3.21481528 | 0.001305285 | 0.006868285 |
| Mapt_e9 | 57.75492694 | -0.996202515 | 0.316531631 | -3.147244752 | 0.001648169 | 0.008470824 |
| Vwf | 101.5271005 | 1.004310317 | 0.325215055 | 3.088142147 | 0.002014121 | 0.010116381 |
| Cd36 | 35.24305784 | 1.966408735 | 0.640904947 | 3.068175312 | 0.002153702 | 0.010577071 |
| Fcgr4 | 32.79813803 | 1.731137258 | 0.571557167 | 3.028808592 | 0.002455202 | 0.011795644 |
| H2Eb1 | 21.14925237 | 2.375979159 | 0.79233299 | 2.998712903 | 0.002711227 | 0.012748533 |
| Ppp1r2 | 483.5866223 | -0.477162077 | 0.160609352 | -2.970948273 | 0.002968818 | 0.013668932 |
| C1ra | 21.63475553 | 1.742964536 | 0.606341667 | 2.874558407 | 0.004045929 | 0.018247967 |
| Dnm1l | 547.3444049 | -0.391886783 | 0.13685888 | -2.863437022 | 0.004190721 | 0.018522987 |
| Gfap | 1948.747866 | -0.628620116 | 0.220307497 | -2.853375965 | 0.004325741 | 0.018744878 |
| Stat6 | 42.47588209 | 1.139285801 | 0.408454124 | 2.789262573 | 0.005282822 | 0.022451992 |
| Hprt1 | 1515.649519 | -0.32691555 | 0.119792109 | -2.729024071 | 0.006352207 | 0.02564728 |
| Cxcl9 | 35.38025541 | 1.690802131 | 0.62036167 | 2.725510315 | 0.006420217 | 0.02564728 |

| | | | | | | |
|---|---|---|---|---|---|---|
| Cfl1 | 1643.888738 | -0.311767855 | 0.114401628 | -2.725204698 | 0.006426163 | 0.02564728 |
| Cd163 | 15.13231943 | 2.041097061 | 0.749992815 | 2.72148882 | 0.006498858 | 0.02564728 |
| Igfbp5 | 94.4112119 | 0.662821966 | 0.252792306 | 2.622002131 | 0.008741488 | 0.033870809 |
| Mpo | 22.52174356 | 1.971208038 | 0.754645844 | 2.61209686 | 0.008998875 | 0.033870809 |
| Mapk8 | 287.7355335 | -0.414754741 | 0.158948158 | -2.609371162 | 0.00907088 | 0.033870809 |
| Prkcb | 423.0504557 | -0.537987268 | 0.206545464 | -2.604691758 | 0.009195695 | 0.033870809 |
| Ccl5 | 7.624058467 | 2.85716738 | 1.126912012 | 2.535395265 | 0.011232051 | 0.040579498 |
| C1qa | 217.7239149 | 0.701361929 | 0.277763614 | 2.525031698 | 0.011568785 | 0.040579498 |
| B2m | 1495.04158 | 1.285557041 | 0.509139619 | 2.524959741 | 0.011571154 | 0.040579498 |
| Zbp1 | 11.63776236 | 1.918925358 | 0.761623682 | 2.519519027 | 0.011751529 | 0.040579498 |
| Mmp9 | 19.18729103 | -1.326096287 | 0.528737664 | -2.508042035 | 0.012140222 | 0.041276756 |
| Ccl19 | 16.20575406 | 1.543636658 | 0.618004364 | 2.497776306 | 0.012497503 | 0.041847699 |
| Grin2a | 87.98988819 | -0.620068073 | 0.252850346 | -2.452312538 | 0.014194132 | 0.046817442 |
| Ms4a6d | 24.35410604 | 1.208626676 | 0.493923241 | 2.446992923 | 0.014405367 | 0.046817442 |
| Tnfsf11 | 13.70557618 | 1.792050918 | 0.736018215 | 2.434791532 | 0.014900371 | 0.047724378 |
| Tardbp | 440.5455382 | -0.388380867 | 0.160709794 | -2.4166596 | 0.015663658 | 0.049452405 |
| Fcgrt | 98.57220111 | 0.60169015 | 0.254262689 | 2.3664115 | 0.017961468 | 0.055908231 |
| Adam17 | 120.5300511 | -0.539283937 | 0.228981753 | -2.355139352 | 0.018515761 | 0.056833099 |
| Apoe | 6439.702315 | -0.272513486 | 0.116087206 | -2.347489411 | 0.01890041 | 0.057219051 |
| Sod1 | 2778.195675 | -0.285198675 | 0.123376807 | -2.311606876 | 0.020799357 | 0.062117 |
| Map2k6 | 41.64077416 | -0.807787573 | 0.351819306 | -2.296029694 | 0.021674184 | 0.063866594 |
| Ccl11 | 6.826978397 | 2.704764853 | 1.232630703 | 2.194302679 | 0.028213657 | 0.081443358 |
| Tgfb3 | 75.95893296 | -0.652322133 | 0.297585983 | -2.1920459 | 0.028376193 | 0.081443358 |
| Mfn1 | 182.6115942 | -0.455122615 | 0.208364115 | -2.184265822 | 0.028942721 | 0.082004375 |
| P2ry6 | 19.87870079 | 1.229648634 | 0.571044354 | 2.153332969 | 0.031292519 | 0.087539833 |
| Cat | 542.003672 | -0.375179995 | 0.174997452 | -2.143916905 | 0.032039545 | 0.088509244 |
| Arc | 28.23195534 | -0.967378044 | 0.45559534 | -2.123327346 | 0.033726431 | 0.091168748 |
| Ctsd | 2467.524839 | -0.235102444 | 0.110786379 | -2.122124099 | 0.033827318 | 0.091168748 |
| Gls | 573.5314787 | -0.353339346 | 0.169706714 | -2.082058736 | 0.037337105 | 0.099415665 |
| Cxcr2 | 14.12644643 | 1.382134749 | 0.666891325 | 2.072503716 | 0.03821849 | 0.099437764 |
| Hspb2 | 13.54617795 | 1.541686685 | 0.743979691 | 2.072216088 | 0.038245294 | 0.099437764 |
| IL6ra | 31.57382726 | 0.855351787 | 0.420141389 | 2.035866519 | 0.041763761 | 0.107323152 |
| Ccl7 | 17.26462058 | 1.180749424 | 0.584082143 | 2.021546863 | 0.043223186 | 0.10979683 |
| Aifm1 | 174.2078758 | -0.440965995 | 0.221812874 | -1.988009018 | 0.046810693 | 0.117345145 |
| Cd55 | 109.8327142 | 0.835556247 | 0.42114883 | 1.983992803 | 0.047256642 | 0.117345145 |
| Masp2 | 5.435618255 | -1.914729807 | 0.987296338 | -1.939366869 | 0.052456682 | 0.128810296 |
| Casp8 | 21.47352773 | 0.974097346 | 0.525124121 | 1.854984958 | 0.063598375 | 0.152852846 |
| Fcgr3 | 38.2105756 | 0.708442436 | 0.381959869 | 1.854756202 | 0.063631049 | 0.152852846 |
| Stat3 | 306.7519221 | -0.311109558 | 0.1746556 | -1.781274452 | 0.074867626 | 0.17791124 |
| Ccr1 | 19.99209853 | 0.908525597 | 0.524739789 | 1.731383089 | 0.083383461 | 0.196039839 |
| Ly86 | 133.5121134 | 0.563700319 | 0.327314027 | 1.72220031 | 0.085033239 | 0.197814166 |
| Tlr7 | 20.82497919 | 1.073592106 | 0.630600457 | 1.702491798 | 0.088663215 | 0.204110109 |
| C1s1 | 9.414461744 | 1.346719114 | 0.800861052 | 1.681588973 | 0.09264857 | 0.211085918 |
| C3ar1 | 25.13796422 | 0.867515439 | 0.524606037 | 1.653651271 | 0.098198389 | 0.221447387 |
| Ccnd1 | 48.6291091 | -0.598500769 | 0.365494177 | -1.637511093 | 0.101523723 | 0.226633765 |
| Ccl21b | 5.553062117 | -1.656138813 | 1.016173542 | -1.629779506 | 0.103148107 | 0.227957316 |
| Tyrobp | 195.7146895 | 0.312043209 | 0.195819308 | 1.593526259 | 0.111042182 | 0.242973488 |
| Csf1 | 149.9672132 | -0.351346755 | 0.221399594 | -1.586934957 | 0.112527377 | 0.243809316 |
| LRRK2_h | 19.76827858 | 0.883688279 | 0.564504885 | 1.565421846 | 0.117484029 | 0.252077383 |
| Ide | 189.4454331 | -0.347807144 | 0.227673141 | -1.527659967 | 0.126596985 | 0.268522942 |
| Ace | 79.68512021 | -0.514717744 | 0.337803456 | -1.523719592 | 0.127578773 | 0.268522942 |
| Myl2 | 28.28987771 | 2.579495615 | 1.721277272 | 1.498593896 | 0.133979017 | 0.279333611 |
| Clu | 4950.778411 | -0.200465808 | 0.134616176 | -1.48916582 | 0.136443709 | 0.281813642 |
| Calb | 157.8278431 | -0.319412638 | 0.215936408 | -1.479197696 | 0.139087485 | 0.284057987 |
| Ptgs2 | 20.83009553 | 0.91122323 | 0.619397634 | 1.47114419 | 0.141252127 | 0.284057987 |
| Axin1 | 34.64509211 | -0.670725794 | 0.456471159 | -1.469371681 | 0.141732004 | 0.284057987 |
| Ccl2 | 10.73390245 | 1.045401262 | 0.713139453 | 1.465914216 | 0.142671659 | 0.284057987 |

| | | | | | | |
|---|---|---|---|---|---|---|
| Tlr6 | 13.23899156 | 1.007808754 | 0.707122148 | 1.425225836 | 0.15409193 | 0.300915551 |
| Cx3cr1 | 140.3520798 | -0.329619908 | 0.231593685 | -1.423268116 | 0.154658446 | 0.300915551 |
| C1qb | 309.7404124 | 0.310621569 | 0.218544247 | 1.421321189 | 0.155223406 | 0.300915551 |
| Cxcl5 | 22.15726799 | 0.79280397 | 0.572533967 | 1.384728271 | 0.166135571 | 0.319269228 |
| Cxcl10 | 22.35991372 | 0.730732387 | 0.53027081 | 1.378036229 | 0.168192103 | 0.320434954 |
| Trem2 | 45.51483432 | 0.479282112 | 0.354974793 | 1.350186328 | 0.176956222 | 0.334250642 |
| Ccl4 | 15.98951828 | 0.969079784 | 0.727875218 | 1.331381754 | 0.18306343 | 0.342856085 |
| Adam10 | 443.7494934 | -0.213697803 | 0.161674422 | -1.321778673 | 0.186241862 | 0.345877744 |
| Itgax | 2.947336264 | 1.942483241 | 1.47243537 | 1.319231581 | 0.187091703 | NA |
| Tlr4 | 16.0243138 | 0.785514557 | 0.598383642 | 1.312727323 | 0.189274847 | 0.346355885 |
| Igfbp2 | 173.6833261 | 0.300754321 | 0.22929232 | 1.3116633 | 0.189633765 | 0.346355885 |
| Flii | 223.760153 | -0.240422082 | 0.184666891 | -1.301923047 | 0.192942693 | 0.349510944 |
| Eif2s1 | 200.8619943 | -0.235974319 | 0.182777113 | -1.291049608 | 0.196686476 | 0.353396026 |
| Tnfsf9 | 4.99069777 | -1.23201105 | 0.978591108 | -1.258964075 | 0.208043308 | 0.367866558 |
| Mmp2 | 37.42215025 | 0.498344626 | 0.395859693 | 1.258892066 | 0.20806932 | 0.367866558 |
| Marco | 16.87389813 | 0.719849025 | 0.576268882 | 1.249154773 | 0.21160847 | 0.371154539 |
| Cd68 | 25.06931553 | 0.585925868 | 0.472008913 | 1.241344924 | 0.214478356 | 0.373226115 |
| Atp13a2 | 40.36251813 | -0.49645125 | 0.41163744 | -1.206040077 | 0.227802038 | 0.391446013 |
| Ccl3 | 12.14955493 | 0.820812224 | 0.681593484 | 1.204254799 | 0.228491111 | 0.391446013 |
| Ctsb | 177.204747 | -0.246988816 | 0.208029958 | -1.187275228 | 0.235119076 | 0.399702429 |
| IL10rb | 89.83963178 | 0.33900868 | 0.290988305 | 1.165025102 | 0.244008832 | 0.409829468 |
| Trem1 | 17.94692973 | 0.668876259 | 0.575075845 | 1.16310964 | 0.244785022 | 0.409829468 |
| Ptprc | 19.83045719 | 0.648618536 | 0.561896323 | 1.154338459 | 0.248361435 | 0.412690806 |
| Crh | 27.70898389 | 0.555220688 | 0.484701691 | 1.14548948 | 0.252006451 | 0.415622579 |
| Bdnf | 27.06112498 | -0.494697045 | 0.436989227 | -1.132057758 | 0.257610153 | 0.421717361 |
| Ccl8 | 5.257021038 | 1.274627335 | 1.139502402 | 1.118582403 | 0.263318334 | 0.427892293 |
| Mef2c | 99.68049373 | -0.262257473 | 0.23867797 | -1.098792119 | 0.271858751 | 0.438545868 |
| Fos | 35.22356523 | -0.417829285 | 0.382205815 | -1.093204938 | 0.274303829 | 0.439283669 |
| Lrrk2 | 56.65083977 | -0.385464253 | 0.355544595 | -1.08415163 | 0.278297574 | 0.441685874 |
| Nlrp3 | 9.098592334 | 0.876463999 | 0.810965201 | 1.080766472 | 0.279801006 | 0.441685874 |
| Arg1 | 15.75262126 | 0.660895138 | 0.61846341 | 1.068608307 | 0.285246204 | 0.447088021 |
| Ptger1 | 3.824300835 | -1.281037354 | 1.204793054 | -1.063284146 | 0.287653113 | 0.447685478 |
| Myc | 20.152012 | -0.52900663 | 0.507793047 | -1.041776041 | 0.297515524 | 0.45979672 |
| C4a | 472.0131281 | -0.532386225 | 0.530867026 | -1.00286173 | 0.31592758 | 0.484861077 |
| Ifih1 | 22.79110323 | 0.513773963 | 0.524563469 | 0.979431458 | 0.32736684 | 0.496011017 |
| P2ry12 | 153.6963153 | -0.214196294 | 0.21883682 | -0.978794584 | 0.327681486 | 0.496011017 |
| MAPT_h_ex1 | 3.245298758 | 1.385301184 | 1.415799145 | 0.978458837 | 0.327847441 | NA |
| Tgfbr1 | 143.8234157 | 0.19537934 | 0.206838405 | 0.94459895 | 0.344863663 | 0.518468501 |
| GRN_h | 2.876445238 | -1.205608574 | 1.28306122 | -0.939634489 | 0.347405079 | NA |
| Msr1 | 6.390367524 | 0.878097812 | 0.936151012 | 0.937987355 | 0.348250908 | 0.520023316 |
| Plg | 13.05582922 | 0.608699916 | 0.679032077 | 0.896422919 | 0.370026931 | 0.548831892 |
| Mef2b | 3.611741276 | -1.041287707 | 1.171479024 | -0.888865857 | 0.374075177 | NA |
| Abca1 | 144.7153889 | -0.185676079 | 0.210686885 | -0.881289213 | 0.378161303 | 0.552498491 |
| Sox10 | 173.1788479 | -0.185019051 | 0.211170157 | -0.876160979 | 0.380942526 | 0.552498491 |
| Itgam | 38.15112747 | 0.338624931 | 0.387794004 | 0.873208268 | 0.382549569 | 0.552498491 |
| MAPT_h_ex10 | 7.129635318 | -0.716090507 | 0.826458551 | -0.866456649 | 0.38623979 | 0.552498491 |
| Ackr3 | 26.2008251 | 0.410743552 | 0.47410923 | 0.866347936 | 0.386299386 | 0.552498491 |
| Ccl17 | 12.45967453 | -0.53827014 | 0.622881106 | -0.864161932 | 0.387498941 | 0.552498491 |
| Th | 10.2026853 | 0.653909054 | 0.770411442 | 0.848779 | 0.396004277 | 0.56100606 |
| Egr1 | 54.86254172 | -0.303589743 | 0.373110541 | -0.813672382 | 0.41583266 | 0.585344062 |
| Ncf1 | 30.83522444 | 0.329533214 | 0.416655508 | 0.790900894 | 0.429001826 | 0.600059517 |
| Il12a | 3.957208592 | -0.87926295 | 1.12981417 | -0.778236787 | 0.436429433 | 0.606609464 |
| Il1rn | 3.380245781 | 0.938999538 | 1.244329807 | 0.754622715 | 0.45047539 | NA |
| Chil3 | 3.541800182 | 0.953993835 | 1.26536329 | 0.753928806 | 0.450891972 | NA |
| Igfbp4 | 133.1702533 | -0.169658548 | 0.23166914 | -0.732331236 | 0.463966419 | 0.640853616 |
| Gapdh | 7591.996137 | -0.076305815 | 0.106404855 | -0.717127193 | 0.473295618 | 0.649679079 |
| Ctsc | 68.32087438 | 0.224164323 | 0.317462546 | 0.706112661 | 0.480118079 | 0.653679334 |

| | | | | | | |
|---|---|---|---|---|---|---|
| Grn | 255.6359105 | -0.137862373 | 0.196700315 | -0.700875205 | 0.483380899 | 0.653679334 |
| Tgfb1 | 29.69575508 | -0.315034509 | 0.451241779 | -0.698150136 | 0.485083307 | 0.653679334 |
| Prph | 258.9469802 | -0.256605704 | 0.370245737 | -0.693068626 | 0.488266486 | 0.653981172 |
| Alox5ap | 18.22681665 | -0.357197641 | 0.523114456 | -0.682828846 | 0.494715 | 0.658626596 |
| Ptpn6 | 25.58409619 | 0.333816251 | 0.497186204 | 0.67141093 | 0.501958784 | 0.66426881 |
| TARDBP_h | 2.353536372 | -0.891539149 | 1.382705972 | -0.644778548 | 0.519070706 | NA |
| Maff | 7.389834769 | 0.557846778 | 0.878357738 | 0.635102025 | 0.525361877 | 0.689822116 |
| Tlr8 | 2.57132713 | 0.902375312 | 1.430179609 | 0.630952439 | 0.528071623 | NA |
| Tlr1 | 8.328035141 | -0.53729939 | 0.857305126 | -0.626730639 | 0.530835816 | 0.689822116 |
| Mmp3 | 3.699787913 | 0.748978881 | 1.199525775 | 0.624395821 | 0.532367669 | 0.689822116 |
| Cebpd | 9.194660963 | -0.469111471 | 0.757526236 | -0.619267622 | 0.53574007 | 0.689822116 |
| Csf1r | 113.3547657 | 0.145101976 | 0.236344623 | 0.613942362 | 0.539253416 | 0.689822116 |
| Dock2 | 9.324058641 | 0.453441179 | 0.751797762 | 0.603142496 | 0.546413898 | 0.689822116 |
| Itgb2 | 36.40840346 | 0.235535005 | 0.391339019 | 0.601869463 | 0.547261033 | 0.689822116 |
| Cd14 | 13.20212413 | -0.379215429 | 0.635703624 | -0.596528657 | 0.550822115 | 0.689822116 |
| Fcer1g | 24.09275392 | 0.278964437 | 0.468114328 | 0.595932275 | 0.551220471 | 0.689822116 |
| Crp | 2.448997565 | 0.840813351 | 1.43750269 | 0.584912541 | 0.558606526 | NA |
| Npy | 140.4071794 | -0.135967025 | 0.232494254 | -0.584818863 | 0.558669519 | 0.689822116 |
| Crhr1 | 36.20544386 | -0.220360137 | 0.378110041 | -0.582793667 | 0.560032209 | 0.689822116 |
| Fcgr1 | 32.97114039 | 0.279434541 | 0.480556036 | 0.5814817 | 0.560915848 | 0.689822116 |
| Ptafr | 10.92563295 | 0.433818905 | 0.747832557 | 0.580101656 | 0.561846067 | 0.689822116 |
| Tnfsf1a | 76.73768768 | 0.165947942 | 0.288689627 | 0.574831677 | 0.565405141 | 0.690356554 |
| Mertk | 59.96106761 | -0.173298949 | 0.305805297 | -0.566697017 | 0.570920048 | 0.693260058 |
| Il1b | 3.137826598 | 0.783645113 | 1.387881542 | 0.564634005 | 0.572322731 | NA |
| Hspb1 | 221.8170085 | 0.107376264 | 0.193729281 | 0.554259344 | 0.57940137 | 0.699714223 |
| Gusb | 52.46634121 | -0.177569527 | 0.335984565 | -0.528505014 | 0.597148868 | 0.717227717 |
| Ms4a4a | 4.119359355 | 0.651989479 | 1.309977342 | 0.497710501 | 0.618688105 | 0.738116016 |
| Il4 | 12.67159836 | 0.330734154 | 0.669336636 | 0.494122294 | 0.621219814 | 0.738116016 |
| Ccr4 | 11.52524151 | 0.396107503 | 0.811937249 | 0.487854823 | 0.625652676 | 0.739407708 |
| APOE_h | 3.084753879 | 0.651344703 | 1.343410346 | 0.484844191 | 0.627786873 | NA |
| Timp1 | 7.681766968 | -0.392758086 | 0.864431287 | -0.454354316 | 0.649573828 | 0.758835353 |
| Tubb5 | 792.0450307 | -0.063737864 | 0.140657932 | -0.453140917 | 0.650447274 | 0.758835353 |
| Treml4 | 15.34347474 | 0.257078567 | 0.579841635 | 0.443359964 | 0.657505389 | 0.758835353 |
| Cfb | 6.672617311 | -0.40369562 | 0.914415314 | -0.441479505 | 0.658865896 | 0.758835353 |
| Cxcr4 | 25.20989567 | 0.207781433 | 0.471228606 | 0.440935527 | 0.659259673 | 0.758835353 |
| Hck | 7.983519811 | 0.358197679 | 0.839837349 | 0.426508394 | 0.669737433 | 0.766901413 |
| Il1r1 | 41.16806211 | 0.154833385 | 0.387553513 | 0.399514853 | 0.689513882 | 0.785477154 |
| Plat | 137.1052242 | -0.096598428 | 0.249138579 | -0.387729706 | 0.69821607 | 0.791311546 |
| Entpd1 | 52.25559796 | -0.132912443 | 0.350620989 | -0.379077257 | 0.704630494 | 0.794506833 |
| Mafk | 20.91751111 | 0.176814537 | 0.492570625 | 0.358962812 | 0.719622909 | 0.807292705 |
| Il1a | 3.645849763 | -0.404622015 | 1.197769245 | -0.337812994 | 0.735504117 | 0.81647426 |
| Gba | 102.7608173 | -0.08712763 | 0.260548894 | -0.334400306 | 0.738077508 | 0.81647426 |
| Tlr2 | 10.3457675 | 0.232027128 | 0.697248861 | 0.332775198 | 0.739303981 | 0.81647426 |
| Ifng | 4.616612105 | -0.351853455 | 1.071313534 | -0.328431821 | 0.742585186 | 0.81647426 |
| Cd33 | 10.43424752 | 0.232027896 | 0.740041181 | 0.313533763 | 0.753875174 | 0.820210356 |
| Casp1 | 22.1655785 | -0.179660751 | 0.579221483 | -0.310176255 | 0.756426926 | 0.820210356 |
| Jak3 | 14.09371413 | 0.189229128 | 0.611860235 | 0.309268551 | 0.757117252 | 0.820210356 |
| Mmp11 | 16.86200501 | -0.155839728 | 0.565903955 | -0.275381938 | 0.783022819 | 0.844136796 |
| Aqp4 | 860.9740772 | -0.040048935 | 0.150731304 | -0.265697529 | 0.790472166 | 0.848030819 |
| Ptger4 | 10.13487136 | -0.186098024 | 0.718029298 | -0.259178873 | 0.795497231 | 0.849298976 |
| Cxcl15 | 3.188572815 | -0.361897294 | 1.399389657 | -0.258610811 | 0.795935541 | NA |
| Fcrls | 26.85689994 | 0.113524809 | 0.449888252 | 0.252340016 | 0.800778261 | 0.850826902 |
| Treml1 | 1.324643607 | 0.446803714 | 1.835141492 | 0.243470989 | 0.807640557 | NA |
| IL6 | 6.103069055 | 0.214686305 | 0.939683383 | 0.228466639 | 0.819283488 | 0.865540215 |
| Ly96 | 28.56767125 | 0.10251088 | 0.456856006 | 0.224383347 | 0.822459028 | 0.865540215 |
| C5ar1 | 8.97494579 | 0.16102236 | 0.784255224 | 0.205318824 | 0.837323038 | 0.873898693 |
| Creb1 | 103.8084728 | 0.051723039 | 0.253475235 | 0.20405559 | 0.838310058 | 0.873898693 |

| | | | | | | |
|---|---|---|---|---|---|---|
| Itm2b | 3060.489289 | 0.023653192 | 0.120071602 | 0.196992391 | 0.843833492 | 0.875526769 |
| Tnfrsf1b | 4.970493371 | -0.188273743 | 1.01059899 | -0.18629916 | 0.852210166 | 0.8800862 |
| Mbl2 | 1.876805606 | -0.272269617 | 1.55824363 | -0.174728529 | 0.86129295 | NA |
| FTL_h | 5.718376902 | 0.171257527 | 1.00134266 | 0.171027895 | 0.864201832 | 0.885917741 |
| Ltbr | 36.66906553 | -0.065917559 | 0.390272881 | -0.168901203 | 0.865874353 | 0.885917741 |
| Il10 | 2.038360007 | -0.218773723 | 1.48770794 | -0.147054215 | 0.883089228 | NA |
| Treml2 | 3.389179665 | 0.183454044 | 1.265797501 | 0.14493159 | 0.884764885 | NA |
| Tlr9 | 7.182794591 | -0.108531547 | 0.820539515 | -0.132268519 | 0.894771906 | 0.911265398 |
| Nos2 | 2.985984958 | -0.154191075 | 1.343861986 | -0.114737285 | 0.90865336 | NA |
| Alox5 | 15.73066783 | -0.062744532 | 0.603962272 | -0.103888164 | 0.9172581 | 0.929880918 |
| Mme | 69.94092337 | 0.028943873 | 0.321102545 | 0.090139034 | 0.92817673 | 0.93665323 |
| Card9 | 27.26259559 | -0.037995941 | 0.448785867 | -0.084663853 | 0.932528634 | 0.9367674 |
| Il7 | 9.455494477 | 0.023379813 | 0.79854562 | 0.029277993 | 0.976642879 | 0.976642879 |
| ITM2b_h | 2.730324383 | -0.032621323 | 1.33472671 | -0.024440451 | 0.980501283 | NA |
| Tnf | 3.132971244 | -0.024933689 | 1.324700741 | -0.018822129 | 0.984983 | NA |

**Supplementary Table 4: Overlapping gene expression changes in Il-10 expressing homozygous M83 mice vs aged, paralyzed homozygous M83 mice**

*Base Mean=gene counts normalized to library depth; lfcSE-log (Fold Change) Standard Error; stat=Wald test statistics*

**Il-10 expressing homozygous M83 mice**

| | baseMean | log2FoldChange | lfcSE | stat | pvalue | padj |
|---|---|---|---|---|---|---|
| Fcgr2b | 243.50367 | 2.199739729 | 0.277773 | 7.919187 | 2.39E-15 | 2.81E-13 |
| Fcgr4 | 118.16825 | 1.785232879 | 0.43249 | 4.127801 | 3.66E-05 | 0.002152 |
| Ms4a6d | 62.848231 | 1.284898175 | 0.325842 | 3.943311 | 8.04E-05 | 0.003777 |
| Ccl5 | 44.787646 | 2.47553824 | 0.65639 | 3.771443 | 0.000162 | 0.006357 |

**Aged, paralyzed homozygous M83 mice**

| | baseMean | log2FoldChange | lfcSE | stat | pvalue | padj |
|---|---|---|---|---|---|---|
| Fcgr2b | 57.116014 | 1.238220275 | 0.338151 | 3.661741 | 2.51E-04 | 1.68E-03 |
| Fcgr4 | 32.798138 | 1.731137258 | 0.571557 | 3.028809 | 2.46E-03 | 0.011796 |
| Ms4a6d | 24.354106 | 1.208626676 | 0.493923 | 2.446993 | 1.44E-02 | 0.046817 |
| Ccl5 | 7.6240585 | 2.85716738 | 1.126912 | 2.535395 | 0.011232 | 0.040579 |

**Supplementary Table 5: Differential gene expression in spinal cord between Il-10-injected vs GFP-injected hemizygous M83 transgenic mice**

*Base Mean=gene counts normalized to library depth; lfcSE-log (Fold Change) Standard Error; stat=Wald test statistics*

| | baseMean | log2FoldChange | lfcSE | stat | pvalue | padj |
|---|---|---|---|---|---|---|
| C1qa | 408.1870973 | 1.259016141 | 0.206992467 | 6.082424936 | 1.18E-09 | 2.72E-07 |
| Il10 | 3106.858481 | 10.53761631 | 1.80346376 | 5.842987558 | 5.13E-09 | 5.90E-07 |
| Ptgds | 10889.65285 | -1.17033923 | 0.216663426 | -5.401646463 | 6.60E-08 | 5.06E-06 |
| C4a | 735.3011699 | 1.326245368 | 0.256591158 | 5.168710331 | 2.36E-07 | 1.36E-05 |
| C1qb | 555.3733337 | 1.174757675 | 0.253688244 | 4.63071389 | 3.64E-06 | 0.000167627 |
| Fcgr2b | 197.1973481 | 1.251605018 | 0.277198999 | 4.515185929 | 6.33E-06 | 0.000242501 |
| Ccl8 | 48.26045983 | 2.986870228 | 0.741140425 | 4.030100269 | 5.58E-05 | 0.001831886 |
| Itgam | 74.99031832 | 1.443786428 | 0.383160029 | 3.768102934 | 0.000164493 | 0.00472917 |
| Ly86 | 253.758417 | 0.82465312 | 0.223401746 | 3.691345903 | 0.000223071 | 0.005700691 |
| Ctsc | 111.168607 | 0.904862556 | 0.252399852 | 3.585035995 | 0.000337032 | 0.00775173 |
| Trem2 | 70.71441104 | 1.009838394 | 0.310389233 | 3.253458202 | 0.001140095 | 0.023838349 |
| Ccr4 | 14.61058246 | -2.195352196 | 0.723863957 | -3.032824298 | 0.002422766 | 0.046436352 |
| Il4 | 14.76007042 | -1.964626615 | 0.669530886 | -2.934333064 | 0.003342653 | 0.059139247 |
| B2m | 2164.991529 | 1.039262354 | 0.362739554 | 2.865037305 | 0.004169601 | 0.068500591 |
| Igfbp2 | 256.057837 | -0.693075868 | 0.253345051 | -2.735699257 | 0.006224789 | 0.088806399 |
| C3ar1 | 42.8424141 | 0.980525343 | 0.359375378 | 2.72841548 | 0.00636394 | 0.088806399 |
| Fcgr4 | 60.81914194 | 0.975838126 | 0.360405124 | 2.707614459 | 0.00677687 | 0.088806399 |
| Plg | 14.02151282 | -1.789572427 | 0.663331789 | -2.69785416 | 0.006978801 | 0.088806399 |
| Ptgs2 | 24.44040547 | -1.183836041 | 0.441534532 | -2.681185631 | 0.007336181 | 0.088806399 |
| Ccl5 | 25.2491704 | 2.149564626 | 0.816984431 | 2.631096194 | 0.008510994 | 0.097876433 |
| Tnfsf11 | 17.37017343 | -1.37146344 | 0.539584266 | -2.541703911 | 0.011031358 | 0.120819634 |
| Igf1 | 52.06466676 | -0.941445056 | 0.378450591 | -2.487630034 | 0.012859743 | 0.132229583 |
| Fcgrt | 136.2603715 | -0.608984519 | 0.246928318 | -2.466240097 | 0.013653978 | 0.132229583 |
| Ccl3 | 16.17533245 | -1.505750593 | 0.611476532 | -2.462483047 | 0.01379787 | 0.132229583 |
| Tyrobp | 328.7432756 | 0.488031686 | 0.204557303 | 2.385794488 | 0.017042272 | 0.154271252 |
| Cx3cr1 | 172.1124041 | 0.575373117 | 0.24202682 | 2.377311394 | 0.017439359 | 0.154271252 |
| Zbp1 | 28.49939952 | 1.527671079 | 0.647028152 | 2.361058128 | 0.018222874 | 0.15523189 |
| Egr1 | 76.82402032 | -0.822285527 | 0.356125005 | -2.308980038 | 0.020944689 | 0.172045659 |
| Ctsd | 3059.272763 | 0.411804872 | 0.180953516 | 2.2757495 | 0.022861007 | 0.180584321 |
| Cxcl5 | 25.76778247 | -1.280204506 | 0.565381589 | -2.26431941 | 0.023554477 | 0.180584321 |
| Cd163 | 24.10118181 | -0.988015473 | 0.441665067 | -2.237024267 | 0.025284754 | 0.18565011 |
| Fcrls | 57.73952698 | 1.015382792 | 0.455581022 | 2.228764463 | 0.025829581 | 0.18565011 |
| Fcgr3 | 53.13111747 | 0.749830777 | 0.341977066 | 2.192634683 | 0.02833371 | 0.197477374 |
| Fcgr1 | 49.11483131 | 0.810599829 | 0.372227592 | 2.177699469 | 0.029428418 | 0.199074595 |
| Cxcr2 | 20.70283823 | -1.277257232 | 0.591762145 | -2.158396313 | 0.030897032 | 0.20085836 |
| Ccl4 | 10.26387565 | -1.470917033 | 0.683678312 | -2.151475345 | 0.0314387 | 0.20085836 |
| C3 | 175.3044766 | 0.733531994 | 0.345100766 | 2.12555887 | 0.033540007 | 0.208491937 |
| Vwf | 151.3719857 | -0.762534544 | 0.361164259 | -2.11132338 | 0.034744526 | 0.208680063 |
| MAPT_h_ex1 | 8.378165554 | -1.77068795 | 0.843607536 | -2.098947525 | 0.035821527 | 0.208680063 |
| Crh | 21.00186902 | -1.014701824 | 0.48465949 | -2.093638616 | 0.036292185 | 0.208680063 |
| Igfbp5 | 193.5542753 | -0.7305549 | 0.358326709 | -2.038795549 | 0.041470437 | 0.232639035 |
| Ptprc | 44.63115438 | 0.755290015 | 0.377314505 | 2.001751869 | 0.045311425 | 0.248133993 |
| Cd68 | 38.14499063 | 0.747911263 | 0.382117362 | 1.95728155 | 0.050314375 | 0.265676347 |
| Cd55 | 155.4823852 | -0.991901629 | 0.510681961 | -1.942307943 | 0.052099832 | 0.265676347 |
| Tlr9 | 12.72434494 | 1.188912539 | 0.61929025 | 1.919798574 | 0.054883347 | 0.265676347 |
| Mmp2 | 43.12178163 | -0.856110715 | 0.446280296 | -1.918325149 | 0.055069795 | 0.265676347 |
| Th | 13.88526834 | -1.654217208 | 0.866409117 | -1.909279549 | 0.056226039 | 0.265676347 |
| Il1r1 | 52.22696356 | -0.682207445 | 0.357722055 | -1.907088016 | 0.056509191 | 0.265676347 |
| Cd36 | 33.80906179 | -0.988496619 | 0.518519594 | -1.906382381 | 0.056600613 | 0.265676347 |
| Ptpn6 | 48.85914991 | 0.684546265 | 0.36314576 | 1.885045459 | 0.059423701 | 0.273349023 |
| P2ry12 | 128.6627708 | -0.544330964 | 0.295310225 | -1.843251327 | 0.065292324 | 0.294455579 |
| Arg1 | 20.20261919 | -0.893515946 | 0.494369019 | -1.807386611 | 0.07070202 | 0.312720474 |
| ITM2b_h | 3.278552875 | -2.139803397 | 1.212075432 | -1.765404479 | 0.077495811 | NA |
| S100a11 | 362.4622866 | -0.541092118 | 0.308566151 | -1.753569266 | 0.079504341 | 0.345018839 |
| Treml4 | 14.30325112 | -0.97519144 | 0.567080064 | -1.719671527 | 0.085492166 | 0.361282945 |
| Tnfrsf1b | 9.675980806 | -1.260401083 | 0.735041146 | -1.714735414 | 0.086393748 | 0.361282945 |

| | | | | | | |
|---|---|---|---|---|---|---|
| Grn | 361.1612043 | 0.371134269 | 0.218496987 | 1.698578426 | 0.089398644 | 0.367173003 |
| Hck | 11.59265787 | 1.081566495 | 0.661986639 | 1.633819222 | 0.102296815 | 0.412776622 |
| Masp2 | 6.162967503 | 1.473187984 | 0.916922525 | 1.606665715 | 0.108127718 | 0.424927823 |
| Il1b | 10.53051442 | 1.134819231 | 0.708368886 | 1.602017329 | 0.109151777 | 0.424927823 |
| Fos | 44.23460379 | -0.595471347 | 0.373481223 | -1.59438095 | 0.110850737 | 0.424927823 |
| Ackr3 | 32.387154 | -0.656370445 | 0.416699331 | -1.575165585 | 0.115218229 | 0.429080006 |
| Tlr7 | 19.32924657 | -0.850226031 | 0.540432615 | -1.573232272 | 0.115665045 | 0.429080006 |
| Csf1r | 152.103514 | 0.35542834 | 0.227809762 | 1.560198023 | 0.118713093 | 0.433397005 |
| Fcer1g | 48.840242 | 0.61443241 | 0.396593931 | 1.549273356 | 0.121316022 | 0.435979454 |
| Ms4a6d | 44.97626123 | 0.667450328 | 0.437221143 | 1.526573769 | 0.126867034 | 0.44891412 |
| Tgfbr1 | 159.5500412 | -0.33028843 | 0.221815387 | -1.48902398 | 0.136481054 | 0.475615795 |
| Ccl21b | 5.345623944 | -1.471813019 | 0.997489511 | -1.475517288 | 0.140073524 | 0.48084941 |
| Cxcr4 | 34.88531195 | -0.606392285 | 0.41453326 | -1.462831438 | 0.143513508 | 0.485413337 |
| Trem1 | 18.99334743 | -0.922167581 | 0.636235982 | -1.449411234 | 0.147222773 | 0.490742577 |
| Tlr1 | 8.962257006 | 1.025747551 | 0.722659705 | 1.419406042 | 0.155780672 | 0.51185078 |
| Lrp1 | 785.0355534 | 0.397773207 | 0.282034941 | 1.410368537 | 0.158430891 | 0.513226829 |
| IL6 | 4.582405507 | -1.301024294 | 0.951855432 | -1.36682972 | 0.171678685 | 0.545250259 |
| Cxcl9 | 43.87238718 | 0.959206181 | 0.705232238 | 1.360128096 | 0.173789392 | 0.545250259 |
| Ubc | 1758.544479 | 0.268069484 | 0.197842201 | 1.354966143 | 0.175428344 | 0.545250259 |
| Ccr1 | 23.84170261 | -0.681075914 | 0.530491907 | -1.283857314 | 0.199191885 | 0.60570649 |
| Ccl2 | 29.43779217 | 0.721775931 | 0.56338818 | 1.281134316 | 0.200146492 | 0.60570649 |
| Gfap | 2246.378396 | 0.383427785 | 0.303651897 | 1.262721521 | 0.206689277 | 0.614265352 |
| Ccl7 | 22.79746325 | -0.663146097 | 0.527055476 | -1.258209291 | 0.208316076 | 0.614265352 |
| Prph | 267.5675747 | -0.717893754 | 0.584341937 | -1.2285508 | 0.219240272 | 0.636678374 |
| Itgb2 | 61.82257044 | 0.474118875 | 0.387772525 | 1.222672686 | 0.221453348 | 0.636678374 |
| Ltbr | 45.55400207 | -0.421237512 | 0.35600942 | -1.183220128 | 0.236721918 | 0.672173347 |
| Stat3 | 359.0998586 | 0.29093974 | 0.248362752 | 1.171430648 | 0.241425719 | 0.6771697 |
| C1ra | 52.10397689 | 0.558813486 | 0.481920893 | 1.159554387 | 0.246230281 | 0.682324876 |
| Chil3 | 14.55504297 | -0.792654678 | 0.723728966 | -1.095236911 | 0.273412859 | 0.748630447 |
| Ccl19 | 37.77808256 | -0.563570307 | 0.518319874 | -1.087302138 | 0.276903304 | 0.749267764 |
| Aqp4 | 1026.216996 | 0.226204115 | 0.210752344 | 1.073317197 | 0.283128822 | 0.75351775 |
| Ifng | 5.822568491 | 0.946537227 | 0.885361991 | 1.069096298 | 0.285026279 | 0.75351775 |
| Mme | 70.6691849 | -0.308511783 | 0.294443075 | -1.047780739 | 0.294739639 | 0.75680817 |
| H2Eb1 | 41.13993097 | 0.570365053 | 0.545535767 | 1.04551358 | 0.295785667 | 0.75680817 |
| Serpina1a | 179.986284 | -0.410275254 | 0.394325213 | -1.04044895 | 0.29813137 | 0.75680817 |
| Mapt_ex1 | 332.5289835 | 0.244522985 | 0.235650622 | 1.0376505 | 0.299432798 | 0.75680817 |
| Gjc2 | 277.5029816 | 0.407933165 | 0.400869706 | 1.017620336 | 0.308858419 | 0.759918905 |
| Eif2s1 | 188.4707293 | -0.298012648 | 0.296102985 | -1.006449321 | 0.314199479 | 0.759918905 |
| Gusb | 76.55387484 | 0.28451668 | 0.283375002 | 1.004028858 | 0.315364704 | 0.759918905 |
| IL10rb | 116.8336258 | -0.250379786 | 0.251242608 | -0.996565784 | 0.318975321 | 0.759918905 |
| Ncf1 | 51.81428391 | 0.380374859 | 0.389357443 | 0.976929723 | 0.328603946 | 0.759918905 |
| Limk1 | 406.4101689 | 0.29557768 | 0.305636211 | 0.967089857 | 0.333499116 | 0.759918905 |
| H2-Ea-ps | 51.9095377 | 1.462690904 | 1.512701006 | 0.966939863 | 0.333574097 | 0.759918905 |
| Cebpd | 8.728360017 | -0.708883237 | 0.736625397 | -0.962338849 | 0.33587942 | 0.759918905 |
| Atp13a2 | 36.40759056 | 0.374344163 | 0.394093228 | 0.949887327 | 0.342169507 | 0.759918905 |
| Prkcb | 444.6138436 | 0.282571783 | 0.298887992 | 0.945410289 | 0.344449451 | 0.759918905 |
| Dlg4 | 370.5296313 | 0.248413792 | 0.263260427 | 0.943604759 | 0.345371659 | 0.759918905 |
| Cxcl15 | 4.010491729 | -0.94981386 | 1.008685822 | -0.941634986 | 0.346379551 | 0.759918905 |
| Cd14 | 17.5919848 | 0.532437096 | 0.571507992 | 0.931635434 | 0.351524964 | 0.759918905 |
| App | 2350.635578 | 0.213054986 | 0.229110752 | 0.929921377 | 0.352411794 | 0.759918905 |
| S100a9 | 105.3488854 | -0.554306128 | 0.600728832 | -0.922722696 | 0.356151735 | 0.759918905 |
| Il1rn | 6.477076287 | 0.779212876 | 0.84533948 | 0.921775091 | 0.356645903 | 0.759918905 |
| Crp | 4.317121584 | -0.908912015 | 0.986425921 | -0.921419435 | 0.356831486 | 0.759918905 |
| Casp1 | 29.11867486 | 0.44441423 | 0.493917477 | 0.899774256 | 0.368240397 | 0.777021021 |
| SOD1_h | 2.919338555 | -1.146088226 | 1.307555687 | -0.876511981 | 0.380751766 NA | |
| Sod1 | 2697.747848 | -0.179388222 | 0.205736943 | -0.871930042 | 0.383246543 | 0.800227917 |
| LRRK2_h | 18.99048417 | -0.448215593 | 0.520962026 | -0.860361351 | 0.389589884 | 0.800227917 |
| Mapt_e9 | 54.0456452 | 0.280894802 | 0.330646694 | 0.849531561 | 0.395585576 | 0.800227917 |
| Ccl11 | 12.30842342 | -0.760654115 | 0.902157724 | -0.843149812 | 0.399144663 | 0.800227917 |

| | | | | | | |
|---|---|---|---|---|---|---|
| APP_h | 3773.913389 | 0.178667912 | 0.215077542 | 0.830713941 | 0.406135249 | 0.800227917 |
| IL6ra | 34.38456053 | -0.326810381 | 0.395425233 | -0.826478318 | 0.408532813 | 0.800227917 |
| Hprt1 | 1539.437356 | -0.153535749 | 0.186118432 | -0.824935753 | 0.409408067 | 0.800227917 |
| Dock2 | 10.99728816 | 0.520141313 | 0.634619956 | 0.819610711 | 0.412438065 | 0.800227917 |
| Grin2a | 94.1457068 | 0.343743451 | 0.419898359 | 0.8186349 | 0.412994749 | 0.800227917 |
| Creb1 | 104.7440298 | -0.214037655 | 0.262037538 | -0.816820586 | 0.414030966 | 0.800227917 |
| Tnfsf9 | 4.293391396 | -0.750839135 | 0.954786432 | -0.786394852 | 0.431636199 | 0.827302714 |
| Mfn1 | 192.3549081 | 0.185849398 | 0.23935239 | 0.776467693 | 0.437472889 | 0.831560037 |
| C2 | 50.78237511 | 0.395107967 | 0.520825341 | 0.758618937 | 0.448080541 | 0.844742004 |
| Adam10 | 473.7963142 | -0.161684456 | 0.217591076 | -0.743065657 | 0.45744193 | 0.846644052 |
| Il7 | 9.623369464 | -0.495123539 | 0.667086768 | -0.742217598 | 0.457955508 | 0.846644052 |
| Msr1 | 11.16893388 | 0.494899869 | 0.670025452 | 0.738628461 | 0.460132637 | 0.846644052 |
| Timp1 | 21.79527694 | 0.485684848 | 0.6656387 | 0.729652359 | 0.465602709 | 0.84748171 |
| Cfl1 | 1682.409834 | -0.148397902 | 0.207506498 | -0.715148218 | 0.474517462 | 0.84748171 |
| Cat | 584.3194363 | -0.16609863 | 0.236717345 | -0.701674946 | 0.482881901 | 0.84748171 |
| Scna | 548.2017076 | 0.122825418 | 0.17531146 | 0.700612602 | 0.483544812 | 0.84748171 |
| Ide | 188.3006696 | -0.149326254 | 0.214149468 | -0.697299207 | 0.485615564 | 0.84748171 |
| Bsn | 131.663783 | 0.228641646 | 0.329129186 | 0.694686632 | 0.487251707 | 0.84748171 |
| Igfbp3 | 192.2393994 | -0.224511676 | 0.324289882 | -0.692317856 | 0.488737739 | 0.84748171 |
| Mef2b | 7.756828036 | -0.520918579 | 0.754730633 | -0.690204632 | 0.490065511 | 0.84748171 |
| Il1a | 5.951041934 | -0.701058328 | 1.034924509 | -0.677400449 | 0.498151913 | 0.853776144 |
| MAPT_h_ex10 | 10.32830872 | -0.429883514 | 0.639028966 | -0.672713659 | 0.501129476 | 0.853776144 |
| Snap91 | 900.5756638 | 0.128288447 | 0.193654622 | 0.662460029 | 0.507676443 | 0.855408539 |
| Il12a | 4.364158241 | -0.720043735 | 1.117512113 | -0.644327455 | 0.519363116 | 0.855408539 |
| C5ar1 | 17.60358008 | 0.354276117 | 0.550859856 | 0.643132937 | 0.520137844 | 0.855408539 |
| Mbl2 | 3.21096495 | -0.70775332 | 1.100558748 | -0.643085452 | 0.520168654 | NA |
| Plat | 184.9131625 | -0.169838482 | 0.266327418 | -0.637705587 | 0.523665348 | 0.855408539 |
| Calb | 122.7654627 | -0.189679993 | 0.299243062 | -0.633865968 | 0.526168297 | 0.855408539 |
| Jak3 | 20.80077325 | 0.316295543 | 0.503915614 | 0.627675616 | 0.530216462 | 0.855408539 |
| Ptafr | 13.0328557 | 0.367142194 | 0.586497557 | 0.625991003 | 0.531320843 | 0.855408539 |
| P2ry6 | 28.73850703 | -0.265724592 | 0.426796373 | -0.622602742 | 0.533545609 | 0.855408539 |
| Cfb | 11.06485593 | 0.411302371 | 0.663882583 | 0.619540836 | 0.535560129 | 0.855408539 |
| Bace1 | 450.8736 | 0.150317745 | 0.245552558 | 0.612161188 | 0.540431118 | 0.857235566 |
| S100a8 | 763.1594344 | -0.503989001 | 0.832831362 | -0.605151323 | 0.545078455 | 0.857402238 |
| Cltc | 3956.45938 | 0.127478545 | 0.212191228 | 0.600771986 | 0.547991865 | 0.857402238 |
| Ccnd1 | 61.20936403 | -0.179556511 | 0.311869178 | -0.575743048 | 0.564788873 | 0.864999699 |
| Hspb1 | 272.128422 | -0.193277874 | 0.343081682 | -0.563358184 | 0.573191005 | 0.864999699 |
| Mmp9 | 33.52998768 | 0.216613026 | 0.384903069 | 0.562772926 | 0.573589519 | 0.864999699 |
| Csf1 | 170.8637254 | 0.155215609 | 0.276410749 | 0.561539698 | 0.574429676 | 0.864999699 |
| Hspb2 | 19.87558401 | -0.274701586 | 0.494940149 | -0.555019807 | 0.578881113 | 0.864999699 |
| Ace | 105.9968788 | -0.148078356 | 0.267911618 | -0.552713457 | 0.580459637 | 0.864999699 |
| Nlrp3 | 7.787269387 | -0.423308453 | 0.769202435 | -0.550321259 | 0.582099045 | 0.864999699 |
| Mef2c | 97.50657323 | -0.146035829 | 0.265953303 | -0.549103272 | 0.58293458 | 0.864999699 |
| Ppp1r2 | 481.1767512 | -0.118217065 | 0.225271873 | -0.52477508 | 0.599739557 | 0.884231398 |
| Apoe | 7451.385463 | 0.104332329 | 0.201878324 | 0.51680798 | 0.605290209 | 0.886225373 |
| Tubb5 | 901.1046686 | -0.115314861 | 0.225316948 | -0.511789558 | 0.608798299 | 0.886225373 |
| Mpo | 45.57742202 | -0.273090871 | 0.539910655 | -0.505807523 | 0.612991777 | 0.886717665 |
| Tnfsf1a | 100.9238119 | -0.127682376 | 0.260772941 | -0.489630465 | 0.624395415 | 0.890209563 |
| Ece2 | 342.2962129 | 0.11884789 | 0.244410441 | 0.486263554 | 0.626780316 | 0.890209563 |
| Hmox1 | 49.27100264 | -0.162778513 | 0.334983823 | -0.485929474 | 0.627017171 | 0.890209563 |
| Lrrk2 | 51.61621521 | 0.155082093 | 0.32746471 | 0.47358414 | 0.635796487 | 0.89236253 |
| Serping1 | 57.39299711 | -0.219589336 | 0.464358291 | -0.472887726 | 0.636293282 | 0.89236253 |
| SCNA_h | 15636.38429 | 0.083432931 | 0.181876355 | 0.458734345 | 0.646424947 | 0.898226655 |
| Crhr1 | 29.18827175 | -0.181902257 | 0.398781181 | -0.456145539 | 0.648285325 | 0.898226655 |
| GRN_h | 2.325321892 | 0.581756477 | 1.329304335 | 0.437639795 | 0.661647419 | NA |
| Adam17 | 123.1245077 | 0.109438255 | 0.25124678 | 0.435580726 | 0.663140958 | 0.913307906 |
| Treml1 | 3.183376613 | -0.469608297 | 1.125728626 | -0.417159417 | 0.676561807 | NA |
| Serpina3n | 230.7903682 | 0.158659705 | 0.400587208 | 0.396067827 | 0.692054992 | 0.945661317 |
| APOE_h | 4.696060464 | -0.368232817 | 0.938847069 | -0.392218103 | 0.694897069 | 0.945661317 |

| | | | | | | |
|---|---|---|---|---|---|---|
| Treml2 | 6.538063086 | -0.35669664 | 0.922375529 | -0.386715203 | 0.69896706 | 0.945661317 |
| Entpd1 | 55.7612626 | -0.148519174 | 0.390919248 | -0.3799229 | 0.704002648 | 0.945797147 |
| Cxcl10 | 32.32340857 | 0.212550817 | 0.5660538 | 0.375495786 | 0.70729178 | 0.945797147 |
| Card9 | 38.5295721 | 0.146400858 | 0.396192384 | 0.369519619 | 0.711740453 | 0.946244533 |
| Arc | 41.7226257 | -0.142369746 | 0.397769587 | -0.357920139 | 0.720403079 | 0.952256943 |
| Nos2 | 3.401035139 | -0.384918107 | 1.153860231 | -0.333591623 | 0.73868774 | NA |
| Ptger1 | 4.55611192 | -0.348086238 | 1.058629685 | -0.32880831 | 0.742300582 | 0.97559505 |
| Alox5 | 23.11309674 | -0.167544537 | 0.540845532 | -0.309782604 | 0.756726282 | 0.980941994 |
| Ubqln1 | 131.4872447 | 0.080228461 | 0.266322378 | 0.301245663 | 0.763227172 | 0.980941994 |
| Tlr2 | 15.27927476 | -0.168534264 | 0.567520659 | -0.296965866 | 0.766492569 | 0.980941994 |
| Flii | 252.5653125 | 0.066045927 | 0.224173936 | 0.294619118 | 0.768284856 | 0.980941994 |
| Trf | 9636.229019 | 0.079966792 | 0.272710231 | 0.293229895 | 0.769346435 | 0.980941994 |
| Alox5ap | 29.51501896 | 0.127199379 | 0.455783996 | 0.279078205 | 0.780184809 | 0.980941994 |
| C1s1 | 17.87086616 | 0.18746392 | 0.675641089 | 0.277460804 | 0.781426299 | 0.980941994 |
| Gls | 606.4698624 | -0.053748609 | 0.195897438 | -0.274371169 | 0.783799397 | 0.980941994 |
| Ctsb | 209.1781476 | 0.060807305 | 0.232526943 | 0.261506493 | 0.793701943 | 0.980941994 |
| Mrc1 | 55.37567291 | -0.097874331 | 0.376834456 | -0.259727659 | 0.795073856 | 0.980941994 |
| Itm2b | 3319.024442 | 0.047683263 | 0.184584264 | 0.258327889 | 0.796153864 | 0.980941994 |
| Marco | 21.67532131 | -0.16842654 | 0.657306289 | -0.25623753 | 0.797767431 | 0.980941994 |
| Tlr4 | 19.93365868 | 0.142306554 | 0.587559703 | 0.242199308 | 0.808625731 | 0.980941994 |
| Dnm1l | 532.9253883 | -0.044955547 | 0.187659099 | -0.239559644 | 0.810671653 | 0.980941994 |
| Maff | 9.528285724 | -0.156331784 | 0.675078041 | -0.23157587 | 0.816867451 | 0.980941994 |
| Map2k4 | 847.2931296 | 0.052154372 | 0.229612166 | 0.227141153 | 0.820313985 | 0.980941994 |
| Myl2 | 7.773741083 | -0.171188283 | 0.756065847 | -0.226419806 | 0.820874925 | 0.980941994 |
| Mertk | 62.04213552 | -0.064259379 | 0.294326847 | -0.218326596 | 0.827174654 | 0.980941994 |
| Sox10 | 183.0240443 | -0.047152646 | 0.225529836 | -0.209074982 | 0.834389705 | 0.980941994 |
| Tgfb3 | 90.93107963 | 0.068621311 | 0.331123219 | 0.207237991 | 0.835823999 | 0.980941994 |
| Mmp11 | 17.92159258 | 0.105189749 | 0.521283539 | 0.201789892 | 0.840080984 | 0.980941994 |
| Tlr8 | 2.772973128 | 0.235441805 | 1.200463781 | 0.196125704 | 0.844511778 | NA |
| Mapk8 | 270.2716052 | 0.052632064 | 0.271273367 | 0.194018545 | 0.846161361 | 0.980941994 |
| Mafk | 22.73882322 | -0.085878197 | 0.448861887 | -0.191324323 | 0.848271506 | 0.980941994 |
| Itgax | 3.790167845 | 0.21713541 | 1.138374907 | 0.190741564 | 0.848728073 | 0.980941994 |
| Myc | 26.35331675 | -0.076433712 | 0.437271548 | -0.174796902 | 0.861239223 | 0.98273587 |
| Map2k6 | 38.30780804 | 0.061945308 | 0.357095715 | 0.17346976 | 0.862282196 | 0.98273587 |
| Syp | 819.5605808 | 0.033297287 | 0.196947381 | 0.169066918 | 0.865744006 | 0.98273587 |
| Tubb3 | 1657.779 | -0.040039679 | 0.261930885 | -0.152863525 | 0.8785059 | 0.98273587 |
| Ifih1 | 31.28343385 | -0.062274505 | 0.408406215 | -0.152481775 | 0.878806964 | 0.98273587 |
| Ly96 | 30.53671081 | 0.065651283 | 0.436935502 | 0.150253946 | 0.880564266 | 0.98273587 |
| Axin1 | 32.91214772 | -0.061338402 | 0.408676488 | -0.150090363 | 0.880693323 | 0.98273587 |
| Pink1 | 813.9150588 | -0.036742447 | 0.26098681 | -0.140782775 | 0.888041551 | 0.98273587 |
| Clu | 5244.048954 | 0.031429608 | 0.224649637 | 0.139905004 | 0.888735047 | 0.98273587 |
| Tnf | 2.333077062 | 0.177705463 | 1.281605136 | 0.138658514 | 0.889720003 | NA |
| Gba | 103.3123003 | 0.032982216 | 0.247143759 | 0.133453568 | 0.893834683 | 0.982742186 |
| Ece1 | 121.9162518 | -0.043057061 | 0.333542662 | -0.129090115 | 0.897286343 | 0.982742186 |
| Tgfb1 | 44.93029752 | 0.037111561 | 0.365269149 | 0.101600589 | 0.919073712 | 0.994739976 |
| Ms4a4a | 6.457878812 | 0.089777564 | 0.915093538 | 0.098107527 | 0.921846911 | 0.994739976 |
| Lyz2 | 177.2085129 | 0.038639267 | 0.39756008 | 0.097191013 | 0.922574705 | 0.994739976 |
| Cd33 | 15.60779091 | -0.044678475 | 0.553047361 | -0.080785984 | 0.935612155 | 0.994739976 |
| Gapdh | 7948.216974 | 0.015978094 | 0.201376186 | 0.079344507 | 0.936758606 | 0.994739976 |
| Ccl17 | 12.89988592 | 0.052701561 | 0.746405333 | 0.07060716 | 0.943710412 | 0.994739976 |
| TARDBP_h | 1.893180995 | 0.093962937 | 1.419608421 | 0.066189335 | 0.947227088 | NA |
| Tardbp | 419.2847743 | -0.012824666 | 0.210479384 | -0.060930745 | 0.951414364 | 0.994739976 |
| Npy | 104.2284588 | 0.020833771 | 0.360459882 | 0.057797751 | 0.953909729 | 0.994739976 |
| Tlr6 | 13.81099647 | 0.036375886 | 0.657755114 | 0.055303083 | 0.955897006 | 0.994739976 |
| Casp8 | 30.23289105 | -0.024093766 | 0.45926651 | -0.052461403 | 0.958161049 | 0.994739976 |
| Ptger4 | 9.769784431 | -0.034196988 | 0.673845975 | -0.050748969 | 0.959525555 | 0.994739976 |
| Mmp3 | 3.757848346 | 0.047810003 | 1.018080562 | 0.046960923 | 0.962544372 | NA |
| Igfbp4 | 182.4620204 | 0.01087555 | 0.267650775 | 0.040633359 | 0.967588189 | 0.994739976 |
| Bdnf | 21.89635134 | -0.016087435 | 0.502061542 | -0.032042755 | 0.974437955 | 0.994739976 |

| | | | | | | |
|---|---|---|---|---|---|---|
| Aifm1 | 164.6382644 | 0.006537169 | 0.275877787 | 0.023695887 | 0.981095187 | 0.994739976 |
| Stat6 | 65.12183187 | -0.008588424 | 0.374708043 | -0.022920308 | 0.981713841 | 0.994739976 |
| FTL_h | 3.851541424 | 0.022220199 | 1.084374416 | 0.020491261 | 0.983651483 | 0.994739976 |
| Pgk1 | 3184.979255 | 0.003311447 | 0.200177091 | 0.016542587 | 0.986801528 | 0.994739976 |
| Ftl | 616.5315055 | -0.00262608 | 0.19677968 | -0.013345278 | 0.989352325 | 0.994739976 |
| Ppp2ca | 1936.308609 | 0.002459471 | 0.20472931 | 0.012013281 | 0.990415019 | 0.994739976 |
| Abca1 | 193.5085123 | -0.001049966 | 0.227094247 | -0.004623482 | 0.996311008 | 0.996311008 |

**Supplementary Table 6: Differential gene expression in spinal cord between Il-10 injected, alpha-synuclein seeded vs Il-10 injected, non-seeded (PBS) hemizygous M83 mice**

*Base Mean=gene counts normalized to library depth; lfcSE-log (Fold Change) Standard Error; stat=Wald test statistics*

| | baseMean | log2FoldChange | lfcSE | stat | pvalue | padj |
|---|---|---|---|---|---|---|
| Serpina3n | 1107.715207 | 2.316968978 | 0.600536335 | 3.85816618 | 0.000114241 | 0.01396338 |
| Cd68 | 181.9037449 | 1.995619209 | 0.524066799 | 3.807948169 | 0.000140125 | 0.01396338 |
| Hspb1 | 806.7824896 | 1.685366789 | 0.449039507 | 3.75327062 | 0.000174542 | 0.01396338 |
| Fcrls | 268.0452392 | 1.834853398 | 0.505648099 | 3.628716103 | 0.000284834 | 0.015282635 |
| Timp1 | 107.1407386 | 2.218824137 | 0.616363962 | 3.59986027 | 0.000318388 | 0.015282635 |
| Mmp9 | 35.53329644 | -1.744481496 | 0.493261731 | -3.536624449 | 0.000405276 | 0.01621102 |
| Msr1 | 47.53746296 | 1.977648787 | 0.59202652 | 3.340473305 | 0.000836357 | 0.028675104 |
| Il10 | 4529.896042 | -6.711111992 | 2.05897608 | -3.259441456 | 0.001116318 | 0.030737441 |
| Lyz2 | 591.2669701 | 1.798948023 | 0.553463851 | 3.250344211 | 0.001152654 | 0.030737441 |
| Ctsd | 10165.05661 | 1.517428312 | 0.472932891 | 3.208548911 | 0.001334066 | 0.032017588 |
| Tyrobp | 1071.759644 | 1.452621773 | 0.49285766 | 2.94734543 | 0.003205149 | 0.069930533 |
| Fcgr2b | 643.0209573 | 1.068916843 | 0.375558667 | 2.8462047 | 0.004424374 | 0.081975266 |
| Ccl3 | 24.9457389 | 1.609559025 | 0.565738466 | 2.845058487 | 0.004440327 | 0.081975266 |
| S100a8 | 642.5263649 | -1.360374149 | 0.495861817 | -2.743454128 | 0.006079654 | 0.098512319 |
| C1qa | 1439.383312 | 1.216407604 | 0.444057858 | 2.739299804 | 0.00615702 | 0.098512319 |
| C3ar1 | 150.8249473 | 1.347063203 | 0.517750013 | 2.601763727 | 0.009274572 | 0.137643922 |
| Ms4a6d | 122.7485635 | 1.035086499 | 0.40048543 | 2.584579664 | 0.009749778 | 0.137643922 |
| Igfbp4 | 212.266004 | -0.939113018 | 0.371917708 | -2.525055941 | 0.011567987 | 0.154239831 |
| Cd14 | 52.97200934 | 1.290972163 | 0.516800441 | 2.498009017 | 0.012489302 | 0.1577596 |
| Ccnd1 | 129.5707606 | 0.980229677 | 0.403742949 | 2.427855837 | 0.015188379 | 0.172193453 |
| Bsn | 158.6558681 | -1.071800992 | 0.442727023 | -2.420907096 | 0.015481833 | 0.172193453 |
| Limk1 | 496.8077864 | -1.110714249 | 0.460139738 | -2.413862913 | 0.0157844 | 0.172193453 |
| Trem2 | 225.1772192 | 1.127848732 | 0.473314212 | 2.382875272 | 0.017178013 | 0.179248836 |
| Masp2 | 9.319313421 | -1.594154683 | 0.679604848 | -2.345708229 | 0.018990967 | 0.189909673 |
| Pink1 | 940.0682015 | -0.844795824 | 0.368433052 | -2.292942556 | 0.021851312 | 0.196346467 |
| C2 | 61.70469306 | -1.16610469 | 0.508701699 | -2.292315306 | 0.021887455 | 0.196346467 |
| Tlr7 | 31.36361175 | 1.056052425 | 0.461393117 | 2.288834372 | 0.022088978 | 0.196346467 |
| S100a9 | 91.31921882 | -1.11570085 | 0.495127106 | -2.253362496 | 0.024236302 | 0.200929861 |
| Gusb | 180.8917267 | 0.890852182 | 0.395462405 | 2.252684882 | 0.024279025 | 0.200929861 |
| C1qb | 1651.606801 | 0.907189658 | 0.406518686 | 2.231606294 | 0.025640995 | 0.202010391 |
| Arc | 44.12657268 | -1.063171113 | 0.478994554 | -2.219589147 | 0.02644667 | 0.202010391 |
| Fcer1g | 128.4980084 | 0.971586452 | 0.439142546 | 2.212462583 | 0.026934719 | 0.202010391 |
| Cd55 | 122.2250605 | -0.858257496 | 0.395488119 | -2.170122071 | 0.0299976 | 0.209955816 |
| Atp13a2 | 48.29156962 | -0.933820675 | 0.434039432 | -2.151465064 | 0.03143951 | 0.209955816 |
| Bace1 | 559.1676143 | -0.837999962 | 0.390067139 | -2.148348008 | 0.03168612 | 0.209955816 |
| Igfbp5 | 168.68702 | -0.863659583 | 0.403279832 | -2.14158883 | 0.032226583 | 0.209955816 |
| Apoe | 16116.61394 | 0.822479876 | 0.386098952 | 2.130230789 | 0.033152564 | 0.209955816 |
| Tubb3 | 1883.437422 | -0.928972584 | 0.436314333 | -2.129136071 | 0.033243004 | 0.209955816 |
| Fos | 74.69954519 | 0.829376012 | 0.395300747 | 2.098088654 | 0.035897315 | 0.220906552 |
| Mpo | 45.1834093 | -1.00675496 | 0.482484832 | -2.086604373 | 0.036923912 | 0.22154347 |
| Grin2a | 120.8524001 | -0.964522136 | 0.471159985 | -2.047122351 | 0.040646073 | 0.237928232 |
| Hmox1 | 95.27251117 | 0.831598656 | 0.414605011 | 2.005761228 | 0.044881729 | 0.256467024 |
| Fcgr3 | 133.3284406 | 0.767951213 | 0.385848991 | 1.990289545 | 0.046559049 | 0.25986446 |
| Grn | 823.8716915 | 0.765977863 | 0.390956198 | 1.959242151 | 0.050084435 | 0.273187827 |
| Abca1 | 389.2620291 | 0.733057924 | 0.378067839 | 1.938958696 | 0.052506368 | 0.274427894 |
| Calb | 139.6674365 | -0.734255681 | 0.378833569 | -1.938201206 | 0.05259868 | 0.274427894 |
| Ece2 | 434.794067 | -0.708271672 | 0.375790946 | -1.884749165 | 0.059463711 | 0.286488354 |
| Snap91 | 1110.286113 | -0.823085292 | 0.437269257 | -1.882330576 | 0.059791145 | 0.286488354 |
| App | 3069.172239 | -0.712421085 | 0.378953199 | -1.87997116 | 0.060112009 | 0.286488354 |
| Ace | 118.3526832 | -0.828178433 | 0.441001755 | -1.877948158 | 0.060388257 | 0.286488354 |
| Mfn1 | 250.6778236 | -0.707320973 | 0.378206082 | -1.870199891 | 0.061456065 | 0.286488354 |
| H2Eb1 | 54.10817 | -0.931500569 | 0.499312141 | -1.865567633 | 0.062101882 | 0.286488354 |
| Dlg4 | 477.7231685 | -0.800459572 | 0.431098987 | -1.856788338 | 0.063341274 | 0.286488354 |
| Map2k4 | 1040.417637 | -0.752619929 | 0.407044757 | -1.848985686 | 0.06445988 | 0.286488354 |
| Ccl2 | 83.77941478 | 1.051878295 | 0.585024284 | 1.798007919 | 0.072175753 | 0.303080091 |
| APP_h | 4920.506574 | -0.678374814 | 0.377373353 | -1.797622458 | 0.072236857 | 0.303080091 |
| Il7 | 8.317992723 | -1.176401975 | 0.655871443 | -1.793647196 | 0.072869498 | 0.303080091 |

| | | | | | | |
|---|---|---|---|---|---|---|
| Tubb5 | 1069.922392 | -0.652450307 | 0.365293339 | -1.786099658 | 0.074083124 | 0.303080091 |
| Lrp1 | 1109.84624 | -0.622411249 | 0.349520492 | -1.780757534 | 0.07495207 | 0.303080091 |
| Itgax | 10.59570586 | 1.395172025 | 0.792450472 | 1.760579462 | 0.078309607 | 0.303080091 |
| Syp | 1001.043598 | -0.741173684 | 0.421500644 | -1.758416492 | 0.078676669 | 0.303080091 |
| Gjc2 | 380.3220751 | -0.772297234 | 0.440336888 | -1.753878122 | 0.079451393 | 0.303080091 |
| Gfap | 4920.483037 | 0.644739099 | 0.369703908 | 1.743933688 | 0.081170654 | 0.303080091 |
| S100a11 | 572.9452617 | 0.670263598 | 0.385054686 | 1.740697159 | 0.081736677 | 0.303080091 |
| Scna | 699.9802561 | -0.68041519 | 0.391331299 | -1.738719063 | 0.082084191 | 0.303080091 |
| Ctsc | 272.1421005 | 0.599257769 | 0.350550134 | 1.709478075 | 0.08736243 | 0.31626023 |
| Cltc | 5096.954171 | -0.658046419 | 0.387535208 | -1.698030026 | 0.089502095 | 0.31626023 |
| Ppp1r2 | 574.0100641 | -0.637141799 | 0.375346994 | -1.697474092 | 0.089607065 | 0.31626023 |
| Mapt_ex1 | 448.6014194 | -0.620996377 | 0.368806449 | -1.683800214 | 0.092220286 | 0.316549933 |
| Cxcr4 | 54.78250704 | 0.783358449 | 0.466080616 | 1.680735953 | 0.092814214 | 0.316549933 |
| Flii | 324.5883541 | -0.602792362 | 0.359797966 | -1.675363452 | 0.093862943 | 0.316549933 |
| Ly86 | 626.084436 | 0.641326943 | 0.384081125 | 1.669769486 | 0.09496498 | 0.316549933 |
| Igfbp2 | 373.053755 | 0.618001358 | 0.372211578 | 1.660349635 | 0.096844136 | 0.31839168 |
| Ptafr | 29.62498465 | 0.78271586 | 0.487699109 | 1.6049155 | 0.108512402 | 0.350505461 |
| SCNA_h | 20100.7803 | -0.609774679 | 0.381038707 | -1.600295897 | 0.109532957 | 0.350505461 |
| Hprt1 | 1838.734552 | -0.571858743 | 0.365285113 | -1.565513411 | 0.117462575 | 0.370934448 |
| Ftl | 1177.592686 | 0.620342176 | 0.398255746 | 1.557647775 | 0.119316767 | 0.371896417 |
| Gls | 750.4879336 | -0.57609096 | 0.375215322 | -1.535360969 | 0.124695186 | 0.381182439 |
| Ccl4 | 11.66090675 | 0.92164556 | 0.601516939 | 1.532202172 | 0.125472553 | 0.381182439 |
| Cebpd | 14.03320328 | 0.933865964 | 0.612736867 | 1.524089726 | 0.127486299 | 0.382458897 |
| Prph | 247.1882388 | -0.733617989 | 0.484471484 | -1.514264539 | 0.129958759 | 0.384485555 |
| Mapk8 | 349.9663143 | -0.578403795 | 0.383683722 | -1.507501522 | 0.131682154 | 0.384485555 |
| Gapdh | 10257.47548 | -0.518028072 | 0.344777324 | -1.502500413 | 0.132967921 | 0.384485555 |
| Tnfsf11 | 20.44102358 | 0.840536614 | 0.563992333 | 1.490333406 | 0.136136593 | 0.388961694 |
| Plg | 12.58069086 | 0.843107773 | 0.574557247 | 1.467404298 | 0.142266106 | 0.395644558 |
| Ubqln1 | 169.2172601 | -0.613817536 | 0.419108035 | -1.464580693 | 0.143035353 | 0.395644558 |
| Trem1 | 15.42893919 | -0.818499672 | 0.570230161 | -1.435384741 | 0.151177492 | 0.395644558 |
| Zbp1 | 47.75514018 | -0.847781184 | 0.595249644 | -1.424244757 | 0.154375633 | 0.395644558 |
| Serping1 | 65.76341449 | -0.601976797 | 0.42345866 | -1.421571582 | 0.15515066 | 0.395644558 |
| Prkcb | 622.0198766 | -0.536874813 | 0.379210512 | -1.415769858 | 0.156842897 | 0.395644558 |
| Alox5 | 40.18135269 | 0.633233759 | 0.448237104 | 1.41272053 | 0.157737913 | 0.395644558 |
| Ms4a4a | 13.85459257 | 0.834684046 | 0.592953204 | 1.407672714 | 0.159228005 | 0.395644558 |
| Dnm1l | 666.0141912 | -0.558927115 | 0.397257211 | -1.406965308 | 0.159437676 | 0.395644558 |
| Ubc | 2455.154339 | -0.533116452 | 0.37918964 | -1.405936227 | 0.159743063 | 0.395644558 |
| C1ra | 74.77821055 | -0.639096347 | 0.455234949 | -1.403882431 | 0.160353862 | 0.395644558 |
| Map2k6 | 47.80802567 | -0.68892366 | 0.491442676 | -1.401839306 | 0.160963238 | 0.395644558 |
| Mme | 81.16954973 | -0.539396481 | 0.384964066 | -1.401160599 | 0.161166054 | 0.395644558 |
| Il1b | 17.74051975 | -0.736422813 | 0.526068418 | -1.399861286 | 0.161554861 | 0.395644558 |
| Ppp2ca | 2475.026491 | -0.529512146 | 0.381502442 | -1.387965284 | 0.165147617 | 0.400357858 |
| Mef2b | 7.015834782 | -0.967293534 | 0.7063273 | -1.369469275 | 0.170852633 | 0.405913308 |
| Tnfsf9 | 7.482853994 | 0.968580382 | 0.708774748 | 1.366555998 | 0.171764517 | 0.405913308 |
| P2ry6 | 47.32068067 | 0.550198326 | 0.403320083 | 1.364172898 | 0.172513156 | 0.405913308 |
| Lrrk2 | 67.9007215 | -0.570414879 | 0.421064062 | -1.354698561 | 0.175513616 | 0.408963767 |
| Tgfb3 | 118.965802 | -0.568420491 | 0.427748068 | -1.328867465 | 0.183891699 | 0.421024558 |
| Casp8 | 54.74940105 | 0.559203025 | 0.421105943 | 1.327939047 | 0.184198244 | 0.421024558 |
| Itgb2 | 130.1777276 | 0.561452906 | 0.425217422 | 1.320390176 | 0.186704782 | 0.421489709 |
| Il1a | 8.855713984 | 0.880644332 | 0.668788932 | 1.316774681 | 0.187914162 | 0.421489709 |
| Crhr1 | 34.47606047 | -0.600619963 | 0.466081348 | -1.288659084 | 0.197516633 | 0.438925851 |
| Mertk | 109.8529276 | 0.4956759 | 0.39199052 | 1.264509915 | 0.206047064 | 0.449783596 |
| MAPT_h_ex1 | 8.066813412 | 0.882646442 | 0.698845174 | 1.263007138 | 0.206586615 | 0.449783596 |
| Sod1 | 3305.421579 | -0.449031806 | 0.356936892 | -1.258014559 | 0.208386491 | 0.449783596 |
| SOD1_h | 4.349474099 | 1.156566669 | 0.922417324 | 1.253843178 | 0.209899011 | 0.449783596 |
| Mapt_e9 | 76.29599372 | -0.499758898 | 0.404675907 | -1.234960841 | 0.216845078 | 0.452291575 |
| Il1r1 | 71.23688722 | 0.480747815 | 0.389956932 | 1.232822847 | 0.217641866 | 0.452291575 |
| Cd163 | 30.05987033 | 0.576556428 | 0.468173441 | 1.231501784 | 0.21813525 | 0.452291575 |
| Tardbp | 546.6827874 | -0.44199101 | 0.359272455 | -1.230239067 | 0.218607594 | 0.452291575 |
| Aifm1 | 214.9575835 | -0.483540784 | 0.396760435 | -1.218722284 | 0.222949617 | 0.457332548 |
| Ece1 | 158.0949909 | -0.452191858 | 0.375226868 | -1.205115884 | 0.228158569 | 0.464051327 |

| | | | | | | |
|---|---|---|---|---|---|---|
| Arg1 | 25.97549728 | 0.5498899 | 0.462123388 | 1.189920082 | 0.234077805 | 0.47208969 |
| Tlr2 | 26.58352416 | 0.613104283 | 0.524686545 | 1.168515353 | 0.24259895 | 0.474928889 |
| Tlr4 | 38.01062536 | 0.551258559 | 0.472010273 | 1.167895256 | 0.242849018 | 0.474928889 |
| Ptpn6 | 107.0048296 | 0.466750217 | 0.400640342 | 1.16501053 | 0.24401473 | 0.474928889 |
| Npy | 138.383451 | -0.443651504 | 0.380813877 | -1.165008762 | 0.244015446 | 0.474928889 |
| Ptgs2 | 29.07104825 | 0.640505012 | 0.551377852 | 1.161644433 | 0.245379926 | 0.474928889 |
| C3 | 397.0893854 | 0.516701359 | 0.45729172 | 1.129916281 | 0.258511503 | 0.494344174 |
| Maff | 17.38588287 | 0.668485301 | 0.592891061 | 1.127501063 | 0.259530691 | 0.494344174 |
| Marco | 23.97896004 | -0.69671939 | 0.622240072 | -1.11969547 | 0.262843556 | 0.49634467 |
| Ncf1 | 99.86826667 | 0.409306617 | 0.366988762 | 1.115311038 | 0.264717157 | 0.49634467 |
| MAPT_h_ex10 | 10.90842616 | -0.647053129 | 0.587179211 | -1.101968729 | 0.270475267 | 0.501255271 |
| Cat | 739.2805925 | -0.366265157 | 0.333283059 | -1.09896122 | 0.271784981 | 0.501255271 |
| C5ar1 | 35.68070709 | 0.516040604 | 0.471353662 | 1.094805548 | 0.273601835 | 0.501255271 |
| Sox10 | 241.8110554 | -0.371515165 | 0.345549609 | -1.075142774 | 0.282310811 | 0.513292384 |
| Pgk1 | 4272.912453 | -0.367979315 | 0.347932182 | -1.057617934 | 0.290229663 | 0.5237227 |
| Il1rn | 9.188111943 | -0.692512807 | 0.664974847 | -1.041412033 | 0.29768436 | 0.533166018 |
| Aqp4 | 1903.015634 | 0.353509421 | 0.346679373 | 1.019701339 | 0.307870127 | 0.545238927 |
| C1s1 | 24.40404021 | -0.486924269 | 0.482589059 | -1.008983233 | 0.312982675 | 0.545238927 |
| Cfb | 23.23087144 | 0.502842467 | 0.499970928 | 1.005743413 | 0.314539014 | 0.545238927 |
| Fcgr4 | 138.5914857 | 0.415435641 | 0.414219843 | 1.002935152 | 0.315892151 | 0.545238927 |
| Bdnf | 29.25357689 | -0.45648712 | 0.455848585 | -1.001400761 | 0.316633096 | 0.545238927 |
| Ptprc | 96.53208796 | 0.400868717 | 0.40148675 | 0.998460641 | 0.318056041 | 0.545238927 |
| Tlr1 | 21.01745942 | 0.481792161 | 0.48718858 | 0.988923347 | 0.322700647 | 0.549277697 |
| Ide | 241.2926973 | -0.343953635 | 0.352077961 | -0.97692464 | 0.328606462 | 0.555015385 |
| Ptger4 | 17.42593774 | 0.506771236 | 0.520988178 | 0.972711583 | 0.330696667 | 0.555015385 |
| Axin1 | 41.70002877 | -0.422261733 | 0.442971379 | -0.953248344 | 0.340464257 | 0.564163051 |
| Serpina1a | 198.3671029 | -0.434263154 | 0.459125763 | -0.945847934 | 0.344226153 | 0.564163051 |
| Jak3 | 29.67919116 | -0.43418537 | 0.460120136 | -0.943634794 | 0.345356306 | 0.564163051 |
| Clu | 8979.402242 | 0.322266589 | 0.341653292 | 0.94325621 | 0.345549869 | 0.564163051 |
| Nlrp3 | 11.83125731 | 0.52961554 | 0.570263398 | 0.928720906 | 0.353033743 | 0.565605546 |
| Creb1 | 129.8511513 | -0.358811818 | 0.387801537 | -0.925245991 | 0.354837965 | 0.565605546 |
| Ptger1 | 6.944964116 | 0.654147504 | 0.709684269 | 0.921744404 | 0.356661913 | 0.565605546 |
| Treml2 | 6.666602742 | -0.675805507 | 0.734012497 | -0.920700274 | 0.357206933 | 0.565605546 |
| Tlr8 | 5.778656822 | 0.705132874 | 0.767476388 | 0.918768167 | 0.358216846 | 0.565605546 |
| C4a | 1794.257809 | 0.358045244 | 0.392514894 | 0.91218257 | 0.361672618 | 0.567329597 |
| Fcgr1 | 104.5659453 | 0.351796091 | 0.399151879 | 0.881358974 | 0.378123556 | 0.589283463 |
| Adam10 | 613.2576425 | -0.288882504 | 0.339855299 | -0.850016183 | 0.395316089 | 0.60949307 |
| Mmp2 | 41.36469034 | -0.372683738 | 0.43923672 | -0.848480378 | 0.396170496 | 0.60949307 |
| Cd36 | 29.44045474 | -0.382899761 | 0.470873205 | -0.813169569 | 0.416120844 | 0.631956237 |
| Tgfb1 | 76.48319568 | 0.339190442 | 0.419300463 | 0.808943639 | 0.418547566 | 0.631956237 |
| Egr1 | 75.92129403 | -0.314826769 | 0.389285835 | -0.808729063 | 0.418671007 | 0.631956237 |
| Ccl21b | 3.533581677 | -0.723102732 | 0.910168803 | -0.794471014 | 0.426921277 | 0.637314632 |
| Ifng | 9.814170791 | -0.480681024 | 0.60583261 | -0.793422169 | 0.427531899 | 0.637314632 |
| Treml1 | 3.256411804 | -0.75398027 | 0.957947338 | -0.787079039 | 0.431235599 | 0.638867554 |
| Cd33 | 25.90691705 | 0.368476325 | 0.472037569 | 0.780608049 | 0.435033056 | 0.64053947 |
| Tlr6 | 23.83321934 | 0.398243015 | 0.515241813 | 0.772924489 | 0.439567071 | 0.641097952 |
| Hck | 21.0295271 | -0.384505011 | 0.498761769 | -0.770919173 | 0.440754842 | 0.641097952 |
| Entpd1 | 89.54926483 | 0.324509126 | 0.431274284 | 0.75244256 | 0.451784961 | 0.653183075 |
| Tgfbr1 | 233.4783983 | 0.268063466 | 0.358854269 | 0.746998124 | 0.455064693 | 0.653985187 |
| Il4 | 10.72384915 | 0.46427082 | 0.645677387 | 0.719044571 | 0.472113458 | 0.674447797 |
| Tnfsf1a | 158.0508442 | 0.255890426 | 0.358802757 | 0.713178539 | 0.475735285 | 0.675600404 |
| Ccl8 | 110.356564 | -0.432733524 | 0.614969478 | -0.703666669 | 0.481640382 | 0.676571919 |
| Ccr4 | 9.213718182 | 0.465348997 | 0.661949997 | 0.702997204 | 0.482057492 | 0.676571919 |
| Myc | 42.54413678 | 0.306809386 | 0.441879998 | 0.694327392 | 0.487476916 | 0.680200348 |
| Cfl1 | 2234.248382 | -0.224296632 | 0.331121114 | -0.677385472 | 0.498161414 | 0.691050691 |
| LRRK2_h | 21.25248251 | -0.347372036 | 0.516232304 | -0.672898681 | 0.501011751 | 0.691050691 |
| Card9 | 55.35710624 | -0.306745179 | 0.459134085 | -0.668094985 | 0.504072963 | 0.691176028 |
| Cxcl9 | 74.01715158 | -0.424393827 | 0.639406254 | -0.663731116 | 0.50686242 | 0.691176028 |
| Igfbp3 | 236.5715393 | -0.338518956 | 0.519513128 | -0.651608089 | 0.514654028 | 0.69783597 |
| FTL_h | 6.317152438 | 0.466207187 | 0.721765663 | 0.645925971 | 0.518327299 | 0.698868268 |
| Csf1r | 277.6127493 | 0.224317417 | 0.364068983 | 0.616139874 | 0.537802207 | 0.721075585 |

| | | | | | | |
|---|---|---|---|---|---|---|
| Crh | 22.88263068 | 0.291562599 | 0.488506967 | 0.596844301 | 0.550611338 | 0.727816939 |
| GRN_h | 3.363079878 | -0.550866305 | 0.928039552 | -0.593580633 | 0.552792638 | 0.727816939 |
| Stat6 | 90.99689093 | -0.221981386 | 0.375861257 | -0.590593954 | 0.554792517 | 0.727816939 |
| IL6ra | 48.93913078 | 0.235293593 | 0.398570685 | 0.590343449 | 0.554960416 | 0.727816939 |
| IL6 | 4.842965836 | 0.47657069 | 0.820365025 | 0.580925168 | 0.561290889 | 0.732118551 |
| Cxcl15 | 4.677749475 | 0.444712868 | 0.796056498 | 0.558644857 | 0.57640412 | 0.747767507 |
| Ltbr | 63.11998952 | 0.225446087 | 0.413465706 | 0.545259459 | 0.585575083 | 0.755580752 |
| ITM2b_h | 2.218491178 | 0.604772906 | 1.121515243 | 0.539246265 | 0.589716941 | 0.756855967 |
| Alox5ap | 48.61661178 | 0.210472308 | 0.405097807 | 0.519559237 | 0.603370815 | 0.770260615 |
| Vwf | 179.6643888 | 0.217643326 | 0.425202267 | 0.511858339 | 0.608750157 | 0.770367227 |
| Hspb2 | 24.94789881 | -0.245469047 | 0.481072976 | -0.510253244 | 0.609874055 | 0.770367227 |
| Th | 9.268220205 | -0.321398899 | 0.644809373 | -0.498440178 | 0.618173824 | 0.77676292 |
| Itm2b | 4828.01219 | -0.157043376 | 0.32948531 | -0.476632405 | 0.633623904 | 0.79202988 |
| Casp1 | 54.50580316 | 0.187798621 | 0.4078948 | 0.460409452 | 0.645222351 | 0.794687074 |
| Myl2 | 10.23390771 | -0.267879473 | 0.59374424 | -0.451169805 | 0.65186717 | 0.794687074 |
| Ctsb | 342.8539201 | 0.158317782 | 0.353787602 | 0.447493867 | 0.654518515 | 0.794687074 |
| Tnfrsf1b | 8.741554236 | 0.281772012 | 0.631154701 | 0.446438902 | 0.655280238 | 0.794687074 |
| Ccl19 | 41.25028818 | -0.243976234 | 0.550110108 | -0.443504366 | 0.657400962 | 0.794687074 |
| Ccl11 | 12.68510197 | -0.269135601 | 0.610176484 | -0.441078292 | 0.659156318 | 0.794687074 |
| Crp | 4.848439262 | 0.35702745 | 0.816245901 | 0.43740183 | 0.661819957 | 0.794687074 |
| Dock2 | 20.95192332 | 0.206833422 | 0.482621399 | 0.428562477 | 0.668241657 | 0.794687074 |
| TARDBP_h | 3.490512175 | 0.405343789 | 0.947253428 | 0.427914829 | 0.66871313 | 0.794687074 |
| Trf | 15757.93644 | 0.143377367 | 0.340225295 | 0.421418893 | 0.673449226 | 0.794687074 |
| Mrc1 | 84.57264969 | 0.158810873 | 0.378082701 | 0.420042684 | 0.674454272 | 0.794687074 |
| Ccl5 | 65.23150087 | 0.252129437 | 0.606235873 | 0.415893298 | 0.677488084 | 0.794687074 |
| Tnf | 4.167494066 | 0.348362565 | 0.841236559 | 0.414107734 | 0.678795209 | 0.794687074 |
| Ptgds | 9434.903142 | -0.176184268 | 0.480946542 | -0.366328174 | 0.714120209 | 0.83198471 |
| H2-Ea-ps | 142.3330366 | 0.515336601 | 1.432538317 | 0.359736696 | 0.719044047 | 0.833674257 |
| Adam17 | 201.0988724 | 0.125239974 | 0.353466732 | 0.354318986 | 0.723099841 | 0.83434597 |
| Csf1 | 260.9590474 | -0.1165681 | 0.345207318 | -0.337675633 | 0.735607639 | 0.844716906 |
| Itgam | 169.4976275 | 0.126002262 | 0.382977104 | 0.329007296 | 0.742150173 | 0.848171626 |
| Cx3cr1 | 322.5386019 | 0.124976101 | 0.387618689 | 0.322420216 | 0.747134371 | 0.849072379 |
| Eif2s1 | 247.364975 | -0.107442884 | 0.337212197 | -0.318620989 | 0.750013935 | 0.849072379 |
| Ccl7 | 27.54282666 | 0.148294447 | 0.487982585 | 0.303892908 | 0.761209481 | 0.857700823 |
| Cxcr2 | 17.14358276 | -0.169510279 | 0.5708149 | -0.296961904 | 0.766495594 | 0.859186553 |
| Ccr1 | 28.90088431 | 0.143749649 | 0.490976606 | 0.292783091 | 0.769687953 | 0.859186553 |
| Cxcl10 | 51.9761112 | 0.119533648 | 0.436976266 | 0.273547232 | 0.78443259 | 0.871591767 |
| Stat3 | 581.1728304 | -0.083971639 | 0.330867124 | -0.253792635 | 0.799655764 | 0.884411905 |
| Mmp3 | 6.53097256 | 0.17772304 | 0.736610168 | 0.241271499 | 0.809344699 | 0.891021687 |
| IL10rb | 164.0457057 | 0.081122972 | 0.34930047 | 0.232244096 | 0.816348431 | 0.894628417 |
| Gba | 153.2336923 | -0.075318091 | 0.351607501 | -0.214210705 | 0.830382749 | 0.905817513 |
| Cxcl5 | 23.13751449 | 0.110410268 | 0.540788001 | 0.204165529 | 0.838224148 | 0.905817513 |
| Il12a | 4.340000665 | -0.16889391 | 0.841658871 | -0.20066789 | 0.840958269 | 0.905817513 |
| Mef2c | 142.5346713 | 0.072653458 | 0.363673832 | 0.199776426 | 0.841655439 | 0.905817513 |
| Tlr9 | 24.96244618 | -0.086684001 | 0.469839066 | -0.18449722 | 0.853623408 | 0.914596509 |
| APOE_h | 5.758495048 | -0.127360044 | 0.73691523 | -0.172828622 | 0.862786138 | 0.920305214 |
| Chil3 | 15.52080604 | -0.095379525 | 0.600421031 | -0.158854405 | 0.873783584 | 0.92539998 |
| Fcgrt | 159.1034231 | -0.05847117 | 0.372516046 | -0.156962824 | 0.875274147 | 0.92539998 |
| Ifih1 | 46.27399236 | 0.044391972 | 0.416131482 | 0.106677753 | 0.915044632 | 0.963204876 |
| Mbl2 | 3.582423616 | -0.088457768 | 0.890868019 | -0.099293909 | 0.920904914 | 0.965140522 |
| P2ry12 | 156.2608608 | -0.037765882 | 0.40127221 | -0.094115369 | 0.925017512 | 0.965235664 |
| Mmp11 | 28.68748798 | 0.040103181 | 0.475921665 | 0.084264247 | 0.932846338 | 0.969191 |
| Ackr3 | 36.91710483 | -0.02835767 | 0.472697979 | -0.059991096 | 0.952162726 | 0.984995924 |
| Igf1 | 53.02446279 | 0.020940078 | 0.39036881 | 0.05364178 | 0.957220569 | 0.985978269 |
| Nos2 | 4.078323139 | -0.038163271 | 0.845326871 | -0.04514617 | 0.9639908 | 0.986889865 |
| Ccl17 | 19.18015273 | 0.014948405 | 0.494326407 | 0.030239948 | 0.975875689 | 0.986889865 |
| B2m | 4302.851882 | -0.011373987 | 0.387386331 | -0.029360838 | 0.976576806 | 0.986889865 |
| Treml4 | 14.39278716 | -0.015188714 | 0.577034236 | -0.026322033 | 0.979000481 | 0.986889865 |
| Mafk | 33.1852568 | -0.009524888 | 0.436170933 | -0.021837512 | 0.982577571 | 0.986889865 |
| Ly96 | 46.53235791 | -0.00916617 | 0.424625644 | -0.021586473 | 0.982777824 | 0.986889865 |
| Plat | 258.0619061 | -0.004478468 | 0.362945898 | -0.012339216 | 0.99015498 | 0.99015498 |

**Supplementary Table 7: Differential gene expression in spinal cord between Il-10-injected, alpha-synuclein seeded vs GFP-injected, alpha-synuclein seeded hemizygous transgenic M83 mice**

*Base Mean=gene counts normalized to library depth; lfcSE-log (Fold Change) Standard Error; stat=Wald test statistics*

| | baseMean | log2FoldChange | lfcSE | stat | pvalue | padj |
|---|---|---|---|---|---|---|
| Fcgr4 | 116.8322379 | 1.729939173 | 0.53276745 | 3.247081206 | 0.001165951 | 0.279828282 |
| Il10 | 53.2990376 | 5.383793401 | 2.026687361 | 2.656449882 | 0.007896819 | 0.947618254 |
| Ms4a6d | 133.3880519 | 1.071105225 | 0.469757093 | 2.280125712 | 0.022600234 | 0.998034871 |
| Ccl8 | 70.06470523 | 1.684293566 | 0.749872869 | 2.246105487 | 0.024697252 | 0.998034871 |
| Fcgr2b | 727.9573023 | 0.879056267 | 0.442591655 | 1.986156444 | 0.047015956 | 0.998034871 |
| Cxcl9 | 48.74468798 | 1.391248252 | 0.701015823 | 1.984617474 | 0.047187047 | 0.998034871 |
| Csf1 | 323.8220365 | -0.746894104 | 0.385631761 | -1.936806507 | 0.052769 | 0.998034871 |
| Arg1 | 24.72732356 | 1.093814039 | 0.565157646 | 1.935414033 | 0.052939509 | 0.998034871 |
| Alox5ap | 43.53835781 | 0.876505021 | 0.484262684 | 1.809978449 | 0.070299129 | 0.998034871 |
| C5ar1 | 34.65569443 | 0.957453324 | 0.555195269 | 1.724534371 | 0.08461142 | 0.998034871 |
| Mpo | 24.67617425 | 0.937502065 | 0.580724383 | 1.61436663 | 0.106447925 | 0.998034871 |
| Tnfsf9 | 7.499352068 | 1.466295976 | 0.918314237 | 1.596725737 | 0.110326856 | 0.998034871 |
| P2ry12 | 193.8573925 | -0.688679505 | 0.463470678 | -1.485918177 | 0.137300765 | 0.998034871 |
| Grn | 1300.293205 | -0.672665258 | 0.467151462 | -1.439929685 | 0.149887294 | 0.998034871 |
| Ccl5 | 58.45551602 | 0.998474951 | 0.712100881 | 1.402153794 | 0.160869326 | 0.998034871 |
| H2-Ea-ps | 118.0926861 | 2.299157664 | 1.674732342 | 1.372850817 | 0.169798729 | 0.998034871 |
| Tgfb1 | 106.8531059 | -0.647590773 | 0.487174101 | -1.329279967 | 0.183755621 | 0.998034871 |
| S100a8 | 312.8362318 | 0.6832842 | 0.519361935 | 1.315622409 | 0.188300808 | 0.998034871 |
| Card9 | 42.31785822 | 0.732658264 | 0.58378094 | 1.255022584 | 0.209470562 | 0.998034871 |
| Il7 | 7.359907355 | -1.006749914 | 0.815140267 | -1.235063406 | 0.216806907 | 0.998034871 |
| Msr1 | 64.16757401 | 0.876759381 | 0.714151608 | 1.227693631 | 0.219561998 | 0.998034871 |
| Ccl4 | 19.44669999 | -0.733457463 | 0.609365029 | -1.203642198 | 0.228727902 | 0.998034871 |
| Tnfsf1a | 203.3301832 | -0.487690583 | 0.406092333 | -1.200935212 | 0.229776334 | 0.998034871 |
| S100a9 | 50.38291519 | 0.637689357 | 0.545335282 | 1.169352833 | 0.242261505 | 0.998034871 |
| Tgfbr1 | 299.6730491 | -0.475433638 | 0.411853367 | -1.154375989 | 0.248346055 | 0.998034871 |
| Il4 | 10.11793393 | 0.932225962 | 0.80994215 | 1.150978452 | 0.2497411 | 0.998034871 |
| Cd14 | 64.54677789 | 0.702627005 | 0.610627564 | 1.150663754 | 0.249870593 | 0.998034871 |
| Gfap | 7090.53993 | -0.490340903 | 0.428096947 | -1.145396869 | 0.252044795 | 0.998034871 |
| Igfbp3 | 177.6462993 | 0.73654045 | 0.643447537 | 1.144678328 | 0.252342432 | 0.998034871 |
| Ctsc | 297.6573081 | 0.465233227 | 0.407767492 | 1.140927701 | 0.253900011 | 0.998034871 |
| Lrp1 | 1018.988171 | -0.451748935 | 0.398937212 | -1.132381039 | 0.257474273 | 0.998034871 |
| Cd36 | 30.88735305 | -0.573405149 | 0.508976118 | -1.12658557 | 0.259917743 | 0.998034871 |
| Mrc1 | 80.77798323 | 0.472176136 | 0.426567262 | 1.106920709 | 0.268328212 | 0.998034871 |
| Trf | 19042.69576 | -0.403578188 | 0.36533163 | -1.104689971 | 0.269293951 | 0.998034871 |
| Treml2 | 6.916201078 | -0.895577602 | 0.814613603 | -1.099389451 | 0.27159823 | 0.998034871 |
| Mmp2 | 43.35938847 | -0.527483532 | 0.488127224 | -1.080627151 | 0.279863 | 0.998034871 |
| SOD1_h | 7.94437098 | -0.859338414 | 0.795455223 | -1.080310228 | 0.280004056 | 0.998034871 |
| Ccl19 | 32.30865314 | 0.719156094 | 0.67462458 | 1.066009328 | 0.286419425 | 0.998034871 |
| Npy | 105.7696803 | 0.483690176 | 0.459669775 | 1.052255775 | 0.292682216 | 0.998034871 |
| Vwf | 173.6977169 | 0.471902417 | 0.452473769 | 1.042938729 | 0.296976671 | 0.998034871 |
| Tlr6 | 23.68033172 | 0.597281111 | 0.573610799 | 1.041265457 | 0.297752364 | 0.998034871 |
| Il1r1 | 75.05394447 | 0.460098176 | 0.441982925 | 1.040986315 | 0.297881899 | 0.998034871 |
| Ifng | 6.930362366 | 0.863886406 | 0.841668067 | 1.026397982 | 0.304704029 | 0.998034871 |
| SCNA_h | 14327.58482 | 0.490031909 | 0.487823961 | 1.004526116 | 0.31512509 | 0.998034871 |
| Chil3 | 12.63795258 | 0.81387026 | 0.811782816 | 1.002571431 | 0.316067686 | 0.998034871 |
| Mapk8 | 256.0167502 | 0.429994247 | 0.435073447 | 0.988325649 | 0.322993188 | 0.998034871 |
| Tlr9 | 29.23326653 | -0.534823615 | 0.542647209 | -0.985582541 | 0.324338009 | 0.998034871 |
| Lyz2 | 802.6811879 | 0.645112074 | 0.677111204 | 0.952741692 | 0.340720964 | 0.998034871 |
| Ccr1 | 26.69670751 | 0.562945786 | 0.596800211 | 0.943273436 | 0.345541059 | 0.998034871 |
| Ccl7 | 25.85394775 | 0.524570846 | 0.560072944 | 0.936611654 | 0.348958355 | 0.998034871 |
| Cx3cr1 | 390.3377023 | -0.434874443 | 0.465016652 | -0.935180367 | 0.349695355 | 0.998034871 |
| Tlr7 | 48.93717938 | -0.43477719 | 0.468979539 | -0.927070701 | 0.353889827 | 0.998034871 |
| Tgfb3 | 86.49236737 | 0.476123834 | 0.522626884 | 0.911020554 | 0.362284544 | 0.998034871 |
| Ccl3 | 44.7255997 | -0.549162765 | 0.603850242 | -0.909435365 | 0.363120361 | 0.998034871 |

| | | | | | | |
|---|---|---|---|---|---|---|
| Tlr8 | 5.873880638 | 0.877495375 | 0.976665693 | 0.898460324 | 0.368940189 | 0.998034871 |
| Il1b | 11.60747021 | 0.645137088 | 0.72320802 | 0.892049133 | 0.372366598 | 0.998034871 |
| Ptgs2 | 30.74024734 | 0.613383839 | 0.698048525 | 0.878712321 | 0.379557277 | 0.998034871 |
| Cebpd | 22.59250835 | -0.581497133 | 0.664380477 | -0.875247171 | 0.381439432 | 0.998034871 |
| Itgam | 163.7830211 | 0.394636705 | 0.455377677 | 0.866614076 | 0.386153498 | 0.998034871 |
| Ptafr | 44.09299928 | -0.476097671 | 0.549605186 | -0.866253964 | 0.386350906 | 0.998034871 |
| Igfbp5 | 136.2942746 | -0.382337875 | 0.443924074 | -0.861268622 | 0.389090113 | 0.998034871 |
| Cxcl10 | 62.58540057 | -0.400387559 | 0.465385727 | -0.860334849 | 0.389604488 | 0.998034871 |
| Ccl17 | 17.18842434 | 0.528159744 | 0.615482519 | 0.858123063 | 0.390824514 | 0.998034871 |
| Egr1 | 77.0452321 | -0.381170828 | 0.444334118 | -0.85784731 | 0.390976782 | 0.998034871 |
| Marco | 16.08140874 | 0.65178866 | 0.793887469 | 0.821008879 | 0.411641211 | 0.998034871 |
| Myl2 | 8.069458109 | 0.636565856 | 0.789088683 | 0.806710159 | 0.41983349 | 0.998034871 |
| Calb | 97.00364216 | 0.366873827 | 0.455988421 | 0.804568297 | 0.421068843 | 0.998034871 |
| Mef2c | 163.0151711 | -0.320111348 | 0.398840279 | -0.802605366 | 0.422202866 | 0.998034871 |
| C2 | 34.75255572 | 0.453956264 | 0.566151682 | 0.801827988 | 0.422652467 | 0.998034871 |
| C3 | 543.9757401 | -0.448318069 | 0.562295742 | -0.79729942 | 0.425277157 | 0.998034871 |
| Hprt1 | 1378.522453 | 0.340486615 | 0.427701911 | 0.796083923 | 0.425983258 | 0.998034871 |
| Th | 7.153200097 | 0.678529337 | 0.853524586 | 0.794973394 | 0.426628979 | 0.998034871 |
| Ltbr | 76.93232676 | -0.353779991 | 0.457989217 | -0.772463581 | 0.439839909 | 0.998034871 |
| Myc | 53.66649648 | -0.381408003 | 0.501597386 | -0.760386742 | 0.447023446 | 0.998034871 |
| Scna | 496.7140854 | 0.382906306 | 0.513578932 | 0.745564668 | 0.455930433 | 0.998034871 |
| Csf1r | 332.6292555 | -0.3149124 | 0.424574289 | -0.741713307 | 0.458261056 | 0.998034871 |
| Map2k4 | 719.7405631 | 0.357875574 | 0.4924589 | 0.726711557 | 0.467402672 | 0.998034871 |
| Masp2 | 4.109320696 | 0.811749168 | 1.175353695 | 0.690642461 | 0.489790257 | 0.998034871 |
| Gapdh | 8022.74969 | 0.258135179 | 0.374781605 | 0.688761603 | 0.490973303 | 0.998034871 |
| Ly96 | 52.38139345 | -0.326757403 | 0.483934196 | -0.675210402 | 0.499542099 | 0.998034871 |
| Treml4 | 12.85200371 | 0.488448605 | 0.731337371 | 0.667884104 | 0.504207575 | 0.998034871 |
| Itgax | 18.17319074 | -0.561422124 | 0.878520905 | -0.639053802 | 0.522787932 | 0.998034871 |
| Plg | 14.78814146 | 0.412287287 | 0.647803875 | 0.636438439 | 0.524490693 | 0.998034871 |
| Gls | 569.3051904 | 0.279424557 | 0.447398648 | 0.62455387 | 0.532263904 | 0.998034871 |
| Prph | 172.1055687 | 0.373295815 | 0.602046823 | 0.620044491 | 0.535228496 | 0.998034871 |
| Atp13a2 | 30.9895659 | 0.342185132 | 0.552856283 | 0.618940478 | 0.535955571 | 0.998034871 |
| Lrrk2 | 51.33953521 | 0.322592823 | 0.528391968 | 0.61051803 | 0.541518703 | 0.998034871 |
| MAPT_h_ex10 | 9.858114204 | -0.433378582 | 0.711756854 | -0.608885717 | 0.542600192 | 0.998034871 |
| Ctsb | 394.415019 | -0.235218513 | 0.388117488 | -0.606049765 | 0.544481708 | 0.998034871 |
| Syp | 702.5393914 | 0.309486075 | 0.514714196 | 0.601277519 | 0.54765516 | 0.998034871 |
| Cd163 | 33.78935516 | 0.314790007 | 0.543594157 | 0.579090124 | 0.562528364 | 0.998034871 |
| Igfbp2 | 491.3766793 | -0.241473119 | 0.418116481 | -0.577525954 | 0.563584211 | 0.998034871 |
| Adam17 | 201.7028914 | 0.222322928 | 0.388376115 | 0.572442331 | 0.567022345 | 0.998034871 |
| Ccl2 | 105.2301558 | 0.408333543 | 0.717133333 | 0.569396964 | 0.569086777 | 0.998034871 |
| Tardbp | 442.1568498 | 0.238498606 | 0.418952887 | 0.569273092 | 0.569170824 | 0.998034871 |
| Sod1 | 2673.891866 | 0.234527232 | 0.417306395 | 0.562002488 | 0.574114324 | 0.998034871 |
| Tubb3 | 1218.701967 | 0.3111622 | 0.554492662 | 0.561165587 | 0.57468466 | 0.998034871 |
| Aqp4 | 2320.687832 | -0.219802882 | 0.39507343 | -0.556359565 | 0.577965075 | 0.998034871 |
| Mafk | 36.35516602 | -0.268080872 | 0.485912078 | -0.55170654 | 0.581149428 | 0.998034871 |
| C1s1 | 22.90143841 | -0.298098453 | 0.541688315 | -0.550313611 | 0.58210429 | 0.998034871 |
| Hck | 20.18022146 | -0.330550046 | 0.60251579 | -0.548616403 | 0.583268726 | 0.998034871 |
| Igfbp4 | 157.28647 | -0.215910791 | 0.402716611 | -0.536135797 | 0.591864702 | 0.998034871 |
| Tubb5 | 798.058454 | 0.226892535 | 0.423554409 | 0.535686869 | 0.59217498 | 0.998034871 |
| Cxcr2 | 14.79958998 | 0.355667144 | 0.667273453 | 0.533015576 | 0.594022798 | 0.998034871 |
| IL6 | 6.446871532 | -0.450522498 | 0.877042047 | -0.513684035 | 0.607472916 | 0.998034871 |
| Casp1 | 55.42589249 | 0.248537136 | 0.484628596 | 0.512840426 | 0.608062949 | 0.998034871 |
| Nlrp3 | 15.68802929 | -0.310409908 | 0.605997906 | -0.51222934 | 0.608490511 | 0.998034871 |
| Serpina3n | 2112.779373 | -0.385299299 | 0.754016632 | -0.510995756 | 0.609354028 | 0.998034871 |
| Pink1 | 730.1320409 | -0.225287849 | 0.446274524 | -0.504818979 | 0.613685984 | 0.998034871 |
| Ccl11 | 13.23725996 | -0.373736076 | 0.741133282 | -0.504276471 | 0.614067109 | 0.998034871 |
| FTL_h | 8.415383227 | -0.386820476 | 0.777329748 | -0.497627265 | 0.618746783 | 0.998034871 |
| Fcrls | 398.9089378 | 0.275274053 | 0.569400372 | 0.483445508 | 0.628779442 | 0.998034871 |

| | | | | | | |
|---|---|---|---|---|---|---|
| GRN_h | 3.157330779 | -0.540779502 | 1.128968238 | -0.479003292 | 0.631936287 | 0.998034871 |
| APOE_h | 4.894747772 | 0.457536511 | 0.960464712 | 0.476369934 | 0.633810851 | 0.998034871 |
| Crhr1 | 25.89214926 | 0.285025408 | 0.599851837 | 0.475159683 | 0.634673161 | 0.998034871 |
| Tyrobp | 1733.276417 | -0.295380555 | 0.624030099 | -0.473343442 | 0.635968173 | 0.998034871 |
| Ccnd1 | 185.8524388 | -0.216376335 | 0.463388704 | -0.466943481 | 0.640540315 | 0.998034871 |
| Dlg4 | 331.8671078 | 0.248387196 | 0.535531294 | 0.463814531 | 0.642780632 | 0.998034871 |
| Il1rn | 8.03666454 | -0.391700683 | 0.857277681 | -0.45691226 | 0.647734112 | 0.998034871 |
| Mef2b | 5.440369753 | -0.418624695 | 0.918476516 | -0.455781599 | 0.648547037 | 0.998034871 |
| Dnm1l | 517.138503 | 0.21387246 | 0.477061073 | 0.448312538 | 0.653927653 | 0.998034871 |
| Gusb | 253.5113586 | -0.204784867 | 0.467265377 | -0.438262446 | 0.661196046 | 0.998034871 |
| Ifih1 | 50.72466008 | -0.203315239 | 0.469380992 | -0.433156099 | 0.66490137 | 0.998034871 |
| Cxcr4 | 66.13084124 | 0.256360199 | 0.594389843 | 0.431299764 | 0.666250422 | 0.998034871 |
| Cfl1 | 2013.643154 | 0.152570805 | 0.368171054 | 0.414401956 | 0.678579756 | 0.998034871 |
| Creb1 | 110.1692143 | 0.188604528 | 0.46130358 | 0.408851212 | 0.682648855 | 0.998034871 |
| Tlr4 | 43.12144045 | 0.228698089 | 0.561229331 | 0.407494899 | 0.68364454 | 0.998034871 |
| TARDBP_h | 4.624868428 | -0.417278805 | 1.024016313 | -0.407492341 | 0.683646418 | 0.998034871 |
| Abca1 | 521.6387541 | -0.18107 | 0.44465195 | -0.407217374 | 0.683848342 | 0.998034871 |
| Ace | 92.41352041 | -0.221915537 | 0.54938397 | -0.403935224 | 0.686260353 | 0.998034871 |
| S100a11 | 753.9748691 | -0.177609234 | 0.443432071 | -0.400533127 | 0.688763889 | 0.998034871 |
| Arc | 31.08761686 | -0.231039041 | 0.58301911 | -0.396280391 | 0.691898192 | 0.998034871 |
| Casp8 | 70.07829237 | -0.175810627 | 0.446063348 | -0.394138248 | 0.693478974 | 0.998034871 |
| Il12a | 4.601750886 | -0.383272691 | 0.997990587 | -0.384044395 | 0.700945543 | 0.998034871 |
| Pgk1 | 3643.208668 | 0.150485689 | 0.393271377 | 0.382651009 | 0.701978544 | 0.998034871 |
| Crh | 24.1401542 | 0.215617803 | 0.569308145 | 0.37873655 | 0.704883509 | 0.998034871 |
| Ncf1 | 111.2770764 | 0.15140516 | 0.412479055 | 0.367061451 | 0.713573181 | 0.998034871 |
| Entpd1 | 106.2370418 | -0.176753149 | 0.485477165 | -0.364081282 | 0.715797314 | 0.998034871 |
| Ece1 | 141.8878244 | -0.157863518 | 0.43373277 | -0.36396493 | 0.715884197 | 0.998034871 |
| Fcer1g | 164.8616595 | 0.193986209 | 0.533803784 | 0.363403586 | 0.716303424 | 0.998034871 |
| Ly86 | 813.3302823 | -0.173453848 | 0.481860277 | -0.359967104 | 0.718871734 | 0.998034871 |
| Cxcl5 | 22.77887627 | 0.251593285 | 0.699484545 | 0.359683837 | 0.71908358 | 0.998034871 |
| Dock2 | 21.59242714 | 0.197465322 | 0.560094018 | 0.352557455 | 0.724420238 | 0.998034871 |
| Ide | 208.1969736 | 0.145837139 | 0.413719309 | 0.352502617 | 0.724461356 | 0.998034871 |
| Adam10 | 582.3391107 | -0.130602795 | 0.37658088 | -0.346812071 | 0.728732508 | 0.998034871 |
| Stat3 | 596.7386241 | -0.129610205 | 0.375018315 | -0.345610335 | 0.729635578 | 0.998034871 |
| Ms4a4a | 17.20310499 | 0.210189588 | 0.610577337 | 0.34424728 | 0.730660328 | 0.998034871 |
| P2ry6 | 54.94267819 | 0.150151848 | 0.44129664 | 0.34025151 | 0.73366713 | 0.998034871 |
| Trem1 | 12.30259563 | -0.24879134 | 0.733593119 | -0.339140777 | 0.734503682 | 0.998034871 |
| Fcgr1 | 125.0069797 | -0.16035814 | 0.47465407 | -0.337842125 | 0.735482163 | 0.998034871 |
| Ptger1 | 7.910038324 | 0.264251621 | 0.783062144 | 0.337459323 | 0.735770671 | 0.998034871 |
| Igf1 | 56.62202535 | -0.148360625 | 0.440015744 | -0.337171174 | 0.735987866 | 0.998034871 |
| Snap91 | 776.5545096 | 0.181273965 | 0.544673365 | 0.332812245 | 0.739276014 | 0.998034871 |
| C1qb | 2297.485519 | -0.167214381 | 0.502822689 | -0.332551384 | 0.739472946 | 0.998034871 |
| Ptpn6 | 132.9338717 | -0.15740549 | 0.477068609 | -0.329943089 | 0.741442965 | 0.998034871 |
| Cat | 679.7506712 | -0.116699774 | 0.360952663 | -0.323310467 | 0.746460127 | 0.998034871 |
| Serping1 | 51.31564881 | 0.145719283 | 0.451042242 | 0.323072362 | 0.746640441 | 0.998034871 |
| Ubqln1 | 130.0179371 | 0.165395118 | 0.514276446 | 0.321607414 | 0.747750126 | 0.998034871 |
| Treml1 | 2.654164615 | -0.379107862 | 1.204817081 | -0.314660099 | 0.753019743 | 0.998034871 |
| Timp1 | 192.3628774 | -0.210612917 | 0.67606973 | -0.311525436 | 0.755401209 | 0.998034871 |
| ITM2b_h | 2.439043794 | 0.392530798 | 1.282090249 | 0.306164717 | 0.759479235 | 0.998034871 |
| Ptger4 | 19.75157476 | 0.17749794 | 0.588343058 | 0.301691229 | 0.762887455 | 0.998034871 |
| Mmp9 | 15.91182271 | 0.208792219 | 0.714262463 | 0.292318622 | 0.770043022 | 0.998034871 |
| Limk1 | 305.8418281 | 0.165485533 | 0.572045335 | 0.289287445 | 0.772361419 | 0.998034871 |
| Alox5 | 47.53686004 | 0.13900925 | 0.500492821 | 0.277744742 | 0.781208312 | 0.998034871 |
| Cd55 | 85.36934221 | 0.134553228 | 0.487713916 | 0.275885562 | 0.782635964 | 0.998034871 |
| Mmp3 | 6.672931452 | 0.244073762 | 0.889515561 | 0.274389536 | 0.783785284 | 0.998034871 |
| Il1a | 10.9185951 | 0.205022785 | 0.751255477 | 0.272906875 | 0.784924801 | 0.998034871 |
| App | 2447.771341 | -0.120367381 | 0.442512742 | -0.272008847 | 0.785615217 | 0.998034871 |
| Hspb1 | 1300.632132 | -0.150684535 | 0.55628071 | -0.270878591 | 0.786484412 | 0.998034871 |

| | | | | | | |
|---|---|---|---|---|---|---|
| Plat | 270.1316634 | -0.108172083 | 0.408472445 | -0.264821005 | 0.791147355 | 0.998034871 |
| Bdnf | 26.15149184 | -0.141685882 | 0.544397813 | -0.260261666 | 0.79466194 | 0.998034871 |
| C3ar1 | 212.3000312 | 0.167491676 | 0.657225078 | 0.254846752 | 0.798841464 | 0.998034871 |
| Aifm1 | 176.4559087 | 0.117926698 | 0.479178352 | 0.24610189 | 0.80560336 | 0.998034871 |
| Mmp11 | 30.87589168 | -0.135302128 | 0.550026559 | -0.245991991 | 0.805688432 | 0.998034871 |
| Fcgr3 | 166.0421028 | 0.108470369 | 0.450620051 | 0.240713587 | 0.809777108 | 0.998034871 |
| LRRK2_h | 18.36388879 | 0.157553571 | 0.659624213 | 0.238853529 | 0.811219161 | 0.998034871 |
| Mbl2 | 3.779792205 | -0.24817551 | 1.060340513 | -0.234052653 | 0.814944103 | 0.998034871 |
| Ccr4 | 11.44872929 | -0.183318888 | 0.795287534 | -0.230506427 | 0.81769827 | 0.998034871 |
| Clu | 10413.34922 | -0.088344806 | 0.387743335 | -0.22784352 | 0.819767893 | 0.998034871 |
| Trem2 | 325.5044194 | -0.130922399 | 0.581565238 | -0.225120744 | 0.821885344 | 0.998034871 |
| Eif2s1 | 237.1477275 | 0.082937204 | 0.379930651 | 0.218295639 | 0.827198772 | 0.998034871 |
| C1ra | 61.63391237 | -0.096183385 | 0.458731119 | -0.209672683 | 0.833923147 | 0.998034871 |
| Ece2 | 327.1256974 | 0.089994839 | 0.439081872 | 0.204961408 | 0.837602277 | 0.998034871 |
| Map2k6 | 36.24272332 | 0.11723367 | 0.639890015 | 0.183209095 | 0.854633959 | 0.998034871 |
| Cltc | 3932.770251 | 0.083143928 | 0.459541314 | 0.180928081 | 0.856424027 | 0.998034871 |
| Nos2 | 3.984463937 | 0.173796711 | 1.037749986 | 0.167474549 | 0.866996672 | 0.998034871 |
| Bace1 | 416.7091949 | -0.076165861 | 0.456346258 | -0.166903661 | 0.867445852 | 0.998034871 |
| Mapt_e9 | 62.95097997 | 0.081176441 | 0.486900226 | 0.166720894 | 0.867589665 | 0.998034871 |
| Ubc | 1998.991424 | 0.074866144 | 0.457681301 | 0.163577021 | 0.870064136 | 0.998034871 |
| Tnf | 4.599745116 | 0.160334099 | 0.998957146 | 0.160501478 | 0.872486057 | 0.998034871 |
| Tlr1 | 24.22302834 | 0.086587918 | 0.544983946 | 0.158881594 | 0.873762162 | 0.998034871 |
| Apoe | 20596.24403 | 0.07395609 | 0.467657248 | 0.158141652 | 0.87434518 | 0.998034871 |
| Hspb2 | 23.90845352 | -0.086332362 | 0.555925642 | -0.155294801 | 0.876588914 | 0.998034871 |
| Mapt_ex1 | 365.8370002 | -0.064509441 | 0.430395431 | -0.149884121 | 0.88085604 | 0.998034871 |
| Cfb | 27.09560986 | 0.084904233 | 0.592703551 | 0.143249071 | 0.886093476 | 0.998034871 |
| Itm2b | 4582.808992 | 0.049207739 | 0.356153019 | 0.1381646 | 0.890110332 | 0.998034871 |
| Axin1 | 37.0186775 | -0.072934088 | 0.531942986 | -0.137108844 | 0.890944762 | 0.998034871 |
| Mfn1 | 190.4054279 | 0.059795017 | 0.438736872 | 0.136289016 | 0.891592807 | 0.998034871 |
| Crp | 5.911455292 | -0.126080036 | 0.937305774 | -0.13451324 | 0.892996743 | 0.998034871 |
| Gjc2 | 292.0588584 | -0.069284745 | 0.53072514 | -0.130547321 | 0.896133417 | 0.998034871 |
| Ctsd | 15660.42239 | -0.077454826 | 0.597897926 | -0.129545233 | 0.896926236 | 0.998034871 |
| Ppp2ca | 2028.79032 | 0.058235333 | 0.454820589 | 0.12804023 | 0.898117136 | 0.998034871 |
| IL6ra | 54.62432823 | -0.05352023 | 0.441966867 | -0.121095572 | 0.903615337 | 0.998034871 |
| Tnfrsf1b | 9.430928818 | 0.0924662 | 0.786754929 | 0.117528593 | 0.906441187 | 0.998034871 |
| Prkcb | 509.4506744 | 0.051392879 | 0.452219181 | 0.113645952 | 0.909518459 | 0.998034871 |
| Cxcl15 | 5.739307141 | -0.09821332 | 0.893945769 | -0.109864964 | 0.912516469 | 0.998034871 |
| C1qa | 2014.539005 | 0.059392797 | 0.549122384 | 0.108159491 | 0.913869178 | 0.998034871 |
| Zbp1 | 35.85888413 | -0.0778211 | 0.723460601 | -0.107567849 | 0.914338502 | 0.998034871 |
| Stat6 | 86.53105795 | -0.043559227 | 0.413867393 | -0.105249236 | 0.916178043 | 0.998034871 |
| Hmox1 | 126.2115801 | -0.046736884 | 0.501805224 | -0.093137501 | 0.925794326 | 0.998034871 |
| Flii | 259.2966824 | 0.036984801 | 0.405235214 | 0.09126749 | 0.927280049 | 0.998034871 |
| Maff | 21.91020697 | -0.058040888 | 0.691990358 | -0.083875284 | 0.933155591 | 0.998034871 |
| C4a | 2040.794179 | 0.04029304 | 0.489713989 | 0.08227872 | 0.934425076 | 0.998034871 |
| Fos | 98.27767368 | -0.034571815 | 0.434133395 | -0.079634084 | 0.936528286 | 0.998034871 |
| Cd33 | 29.94355708 | -0.037424965 | 0.54165645 | -0.069093546 | 0.944915159 | 0.998034871 |
| B2m | 4414.217352 | -0.027550113 | 0.400760588 | -0.068744567 | 0.945192943 | 0.998034871 |
| Ackr3 | 37.94580714 | -0.034514202 | 0.577116792 | -0.059804537 | 0.952311312 | 0.998034871 |
| Itgb2 | 159.5417036 | -0.028949404 | 0.505379686 | -0.057282484 | 0.954320173 | 0.998034871 |
| Sox10 | 215.7405484 | -0.02077078 | 0.378139732 | -0.054928847 | 0.95619515 | 0.998034871 |
| Ftl | 1466.39608 | -0.026009812 | 0.484935617 | -0.053635599 | 0.957225494 | 0.998034871 |
| Fcgrt | 159.1497778 | -0.022425107 | 0.425839203 | -0.052660974 | 0.958002034 | 0.998034871 |
| Cd68 | 299.5642545 | -0.033696817 | 0.658140137 | -0.051200063 | 0.959166101 | 0.998034871 |
| Ptgds | 9028.341495 | -0.029208132 | 0.60047822 | -0.048641451 | 0.961205036 | 0.998034871 |
| Mertk | 131.023588 | -0.020661404 | 0.452981324 | -0.045612045 | 0.963619469 | 0.998034871 |
| Bsn | 103.4197844 | 0.023033721 | 0.545315007 | 0.042239293 | 0.966307939 | 0.998034871 |
| Ppp1r2 | 458.942002 | -0.016674239 | 0.442491298 | -0.037682638 | 0.969940719 | 0.998034871 |
| Mme | 67.62462579 | -0.013664306 | 0.455512883 | -0.029997628 | 0.976068945 | 0.998034871 |

| | | | | | | |
|---|---|---|---|---|---|---|
| Jak3 | 25.89754032 | -0.012721224 | 0.535498399 | -0.023755858 | 0.98104735 | 0.998034871 |
| Ccl21b | 2.750656064 | 0.027643945 | 1.207749178 | 0.022888813 | 0.981738964 | 0.998034871 |
| APP_h | 3857.389443 | -0.007965966 | 0.436137112 | -0.01826482 | 0.985427592 | 0.998034871 |
| H2Eb1 | 38.10360563 | 0.010667045 | 0.605466512 | 0.017617895 | 0.985943681 | 0.998034871 |
| MAPT_h_ex1 | 10.35424625 | 0.011544948 | 0.732305363 | 0.015765211 | 0.987421703 | 0.998034871 |
| Ptprc | 112.3109495 | -0.007181028 | 0.457680521 | -0.015690045 | 0.987481669 | 0.998034871 |
| Tlr2 | 32.57031971 | 0.008365306 | 0.647971384 | 0.012909993 | 0.989699602 | 0.998034871 |
| IL10rb | 172.2294505 | -0.005130043 | 0.401119711 | -0.012789307 | 0.989795887 | 0.998034871 |
| Grin2a | 83.2340164 | 0.005796484 | 0.579268018 | 0.010006566 | 0.992016049 | 0.998034871 |
| Serpina1a | 173.4543574 | -0.003937432 | 0.488467781 | -0.008060781 | 0.993568497 | 0.998034871 |
| Gba | 151.6347544 | 0.001365862 | 0.392150217 | 0.003483007 | 0.997220968 | 0.998034871 |
| Tnfsf11 | 26.26696894 | -0.0015805 | 0.641716211 | -0.002462927 | 0.998034871 | 0.998034871 |

**Supplementary Table 8: Differential gene expression in spinal cord between alpha-Synuclein seeded versus sham-seeded (PBS) hemizygous M83 transgenic mice**

*Base Mean=gene counts normalized to library depth; lfcSE-log (Fold Change) Standard Error; stat=Wald test statistics*

| | baseMean | log2FoldChange | lfcSE | stat | pvalue | padj |
|---|---|---|---|---|---|---|
| C1qa | 663.5805922 | 2.570220191 | 0.264048783 | 9.733883873 | 2.16E-22 | 5.19E-20 |
| Tyrobp | 677.7501635 | 2.373049588 | 0.262242136 | 9.049078169 | 1.44E-19 | 1.73E-17 |
| Ctsd | 5819.154271 | 2.154739123 | 0.271018527 | 7.950523343 | 1.86E-15 | 1.49E-13 |
| C1qb | 853.3748024 | 2.390177102 | 0.31690789 | 7.542182382 | 4.62E-14 | 2.77E-12 |
| Cd68 | 97.51379482 | 2.912921634 | 0.388641268 | 7.495142368 | 6.62E-14 | 3.18E-12 |
| Serpina3n | 786.0019375 | 3.008151559 | 0.406106464 | 7.40729791 | 1.29E-13 | 5.16E-12 |
| Fcrls | 116.9867572 | 2.774988108 | 0.388705122 | 7.139057219 | 9.40E-13 | 3.22E-11 |
| Timp1 | 66.58740151 | 3.030986163 | 0.425820255 | 7.117994335 | 1.10E-12 | 3.29E-11 |
| Trem2 | 118.9611134 | 2.417513441 | 0.344579355 | 7.015839483 | 2.29E-12 | 6.10E-11 |
| Grn | 627.1273883 | 1.943703449 | 0.290276185 | 6.696048613 | 2.14E-11 | 5.14E-10 |
| C3ar1 | 69.47493092 | 2.31126638 | 0.392180351 | 5.89337629 | 3.78E-09 | 8.26E-08 |
| C4a | 775.3807495 | 1.795361808 | 0.326157951 | 5.504577779 | 3.70E-08 | 7.40E-07 |
| Ly86 | 336.5940275 | 1.794061385 | 0.333482447 | 5.379777561 | 7.46E-08 | 1.38E-06 |
| Fcgr2b | 195.5121425 | 1.609234093 | 0.313225771 | 5.137617149 | 2.78E-07 | 4.77E-06 |
| Ptgds | 10825.8052 | -1.179476346 | 0.240509713 | -4.904069495 | 9.39E-07 | 1.50E-05 |
| C3 | 248.6508681 | 1.813808319 | 0.38776226 | 4.677629844 | 2.90E-06 | 4.35E-05 |
| S100a8 | 549.8719864 | -2.392183514 | 0.519431079 | -4.605391576 | 4.12E-06 | 5.81E-05 |
| Hspb1 | 534.9665176 | 1.769530901 | 0.401359284 | 4.408845068 | 1.04E-05 | 0.000136035 |
| Gusb | 112.609631 | 1.518335279 | 0.34498856 | 4.401117763 | 1.08E-05 | 0.000136035 |
| Gfap | 3410.380911 | 1.651688483 | 0.381027374 | 4.334828924 | 1.46E-05 | 0.000175048 |
| Lyz2 | 257.9224335 | 1.342071083 | 0.310533452 | 4.321824505 | 1.55E-05 | 0.000176851 |
| Cx3cr1 | 201.9408027 | 1.27397943 | 0.317030406 | 4.018477115 | 5.86E-05 | 0.000639005 |
| Fcgr3 | 65.14747521 | 1.543576633 | 0.396804751 | 3.890015504 | 0.000100238 | 0.00104596 |
| B2m | 1981.36537 | 1.217589179 | 0.318778289 | 3.819548637 | 0.000133696 | 0.001336961 |
| S100a9 | 78.52184708 | -2.154741077 | 0.576931247 | -3.734831644 | 0.000187841 | 0.001803272 |
| Fcer1g | 62.74375319 | 1.555881582 | 0.426854019 | 3.644996915 | 0.000267395 | 0.002468262 |
| Igfbp3 | 149.6278667 | -1.188148227 | 0.329994458 | -3.600509636 | 0.000317594 | 0.002823058 |
| Ctsc | 108.7262571 | 1.183808671 | 0.329836855 | 3.58907336 | 0.000331855 | 0.002844475 |
| Ptpn6 | 59.13899707 | 1.474575666 | 0.412896576 | 3.571295455 | 0.00035522 | 0.002939751 |
| Mpo | 31.67806764 | -2.049301138 | 0.578289697 | -3.54372756 | 0.000394513 | 0.003156101 |
| Apoe | 9393.689621 | 1.003742488 | 0.28987114 | 3.462719637 | 0.000534745 | 0.004135961 |
| Abca1 | 259.2447004 | 1.061550861 | 0.307301488 | 3.454427988 | 0.000551461 | 0.004135961 |
| Fcgr1 | 56.28852381 | 1.471477282 | 0.427637244 | 3.440947444 | 0.000579681 | 0.004215863 |
| Hprt1 | 1223.295433 | -0.913442629 | 0.269869288 | -3.384759479 | 0.000712406 | 0.005028752 |
| Csf1r | 176.0710446 | 1.034508147 | 0.316485909 | 3.268733666 | 0.001080299 | 0.007407767 |
| Cd55 | 136.4821938 | -1.83355111 | 0.573336378 | -3.198037281 | 0.001383664 | 0.009095894 |
| Ccnd1 | 90.78776286 | 1.162277951 | 0.364162898 | 3.191642965 | 0.001414661 | 0.009095894 |
| Aqp4 | 1212.528103 | 0.949026398 | 0.297829618 | 3.186474218 | 0.001440183 | 0.009095894 |
| Tubb3 | 1227.926364 | -1.13501952 | 0.35817484 | -3.168897963 | 0.001530181 | 0.009416499 |
| Ptprc | 49.06117425 | 1.310876144 | 0.416294875 | 3.148912519 | 0.001638792 | 0.009832755 |
| Itgb2 | 72.55311175 | 1.219863527 | 0.399258452 | 3.05532299 | 0.002248183 | 0.012973886 |
| Syp | 617.6048883 | -0.869221083 | 0.284769356 | -3.052368748 | 0.00227043 | 0.012973886 |
| Ptafr | 20.65507704 | 1.775337644 | 0.590214238 | 3.007954621 | 0.002630124 | 0.014679762 |
| Itgam | 60.37958929 | 1.337102932 | 0.44770883 | 2.986545815 | 0.002821486 | 0.015389921 |
| Ftl | 742.3608177 | 0.801337413 | 0.26914468 | 2.977348138 | 0.002907536 | 0.015506861 |
| Il4 | 14.57849517 | -2.267653847 | 0.76841011 | -2.951098402 | 0.00316646 | 0.016520662 |
| Ccl2 | 35.94085902 | 1.495629473 | 0.511543557 | 2.923757816 | 0.003458337 | 0.017659593 |
| Map2k4 | 631.1308136 | -0.909560761 | 0.313781967 | -2.898703103 | 0.003747095 | 0.018735477 |
| Tgfb1 | 61.78040929 | 1.158978381 | 0.405843471 | 2.85572755 | 0.004293834 | 0.021031025 |
| Igfbp5 | 177.7400209 | -1.090074547 | 0.383380265 | -2.84332462 | 0.004464557 | 0.021429875 |
| Tubb5 | 716.2203498 | -0.837355919 | 0.29566918 | -2.832070353 | 0.004624767 | 0.02176361 |
| Vwf | 143.6181735 | -0.900558643 | 0.322529081 | -2.792178119 | 0.005235453 | 0.024163628 |
| Calb | 95.48406742 | -1.14748914 | 0.41353807 | -2.774808956 | 0.005523414 | 0.025011688 |

| | | | | | | |
|---|---|---|---|---|---|---|
| Msr1 | 16.8714202 | 1.748222853 | 0.636546872 | 2.746416531 | 0.006025022 | 0.026436778 |
| SCNA_h | 11543.4386 | -0.861812814 | 0.314002812 | -2.744602218 | 0.006058428 | 0.026436778 |
| Mmp9 | 21.17772447 | -1.583257708 | 0.600027914 | -2.63864009 | 0.00832393 | 0.035673986 |
| Cxcr2 | 19.3987441 | -1.641785084 | 0.625637587 | -2.624179107 | 0.00868581 | 0.03657183 |
| Csf1 | 205.7948923 | 0.92576578 | 0.355611018 | 2.603310169 | 0.009232838 | 0.038204848 |
| Ccl19 | 31.32642684 | -1.395643884 | 0.53956373 | -2.586615456 | 0.009692369 | 0.039426587 |
| Tlr9 | 14.27391394 | 1.779994205 | 0.690412774 | 2.57815943 | 0.009932816 | 0.039731263 |
| Gls | 480.9485754 | -0.764776377 | 0.297488582 | -2.570775565 | 0.010147106 | 0.039923041 |
| Th | 12.81349236 | -2.497628251 | 0.985286673 | -2.534925438 | 0.011247126 | 0.043537263 |
| Snap91 | 674.1097348 | -0.730460305 | 0.289150453 | -2.52622916 | 0.011529425 | 0.043921618 |
| Sod1 | 2250.664471 | -0.714077519 | 0.292071058 | -2.444875997 | 0.014490195 | 0.054338232 |
| Dnm1l | 428.3759071 | -0.667013775 | 0.279321493 | -2.387978698 | 0.016941324 | 0.061986444 |
| Itgax | 8.031928128 | 2.305265522 | 0.967463689 | 2.38279281 | 0.017181862 | 0.061986444 |
| Scna | 404.4602073 | -0.787884376 | 0.331019904 | -2.380172208 | 0.017304549 | 0.061986444 |
| Tnfsf1a | 125.0461588 | 0.755336985 | 0.323787285 | 2.332818553 | 0.019657671 | 0.068513932 |
| Mapk8 | 205.0472633 | -0.805036345 | 0.34520458 | -2.332055806 | 0.019697756 | 0.068513932 |
| Serpina1a | 161.1634872 | -0.696791257 | 0.302374965 | -2.304394668 | 0.021200499 | 0.072687425 |
| Gapdh | 6331.883202 | -0.601605826 | 0.261722779 | -2.298637616 | 0.021525526 | 0.07276234 |
| Trf | 11294.12354 | 0.770767781 | 0.347533513 | 2.217822889 | 0.026566911 | 0.088556369 |
| Hmox1 | 63.8188783 | 0.862162983 | 0.39067703 | 2.206843292 | 0.027325009 | 0.089835647 |
| Cd14 | 20.78988102 | 1.28012905 | 0.58927414 | 2.172382873 | 0.029826796 | 0.096735554 |
| Limk1 | 282.3902219 | -0.844828798 | 0.390400799 | -2.164003766 | 0.03046406 | 0.097484993 |
| C2 | 31.9948017 | -1.083258259 | 0.501885016 | -2.158379359 | 0.030898349 | 0.097573734 |
| Arg1 | 18.47790879 | -1.290589115 | 0.60788836 | -2.123069301 | 0.033748045 | 0.105188712 |
| Ctsb | 230.8006684 | 0.609469379 | 0.304588036 | 2.000962964 | 0.045396381 | 0.138499506 |
| Ccl5 | 15.3537254 | 1.617125423 | 0.808896375 | 1.999175017 | 0.045589421 | 0.138499506 |
| Tlr1 | 9.657815001 | 1.557788656 | 0.799946414 | 1.947366259 | 0.05149084 | 0.154472521 |
| Marco | 16.0098829 | -1.358693661 | 0.70701906 | -1.921721405 | 0.054640822 | 0.161898733 |
| Tgfb3 | 68.51475205 | -0.835021736 | 0.437676648 | -1.907850783 | 0.056410505 | 0.162903563 |
| Treml4 | 13.25343428 | -1.331036856 | 0.698797712 | -1.90475274 | 0.05681222 | 0.162903563 |
| Cxcl5 | 25.53366991 | -1.255792762 | 0.659837031 | -1.903186248 | 0.057016247 | 0.162903563 |
| Ptgs2 | 25.02641306 | -1.029197965 | 0.544274117 | -1.890955187 | 0.058630327 | 0.165544452 |
| Ppp1r2 | 403.7240795 | -0.590389351 | 0.314065314 | -1.879829849 | 0.060131271 | 0.167808198 |
| Clu | 5820.825749 | 0.582539287 | 0.310853775 | 1.873997793 | 0.060930729 | 0.16808477 |
| Dlg4 | 270.3651288 | -0.662788376 | 0.355303718 | -1.865413567 | 0.062123457 | 0.169169505 |
| Tgfbr1 | 196.2073859 | 0.555625691 | 0.29855106 | 1.861074254 | 0.062733691 | 0.169169505 |
| Casp8 | 37.33739129 | 0.846852117 | 0.459076972 | 1.844684375 | 0.065083465 | 0.173555907 |
| Ncf1 | 53.41984008 | 0.785371037 | 0.435710265 | 1.80250754 | 0.07146559 | 0.188480676 |
| Tardbp | 342.6618183 | -0.552443314 | 0.308542078 | -1.790495861 | 0.073374232 | 0.19141104 |
| Chil3 | 12.38669692 | -1.51120912 | 0.849762646 | -1.77838968 | 0.075339876 | 0.193649445 |
| Trem1 | 17.17294813 | -1.308673943 | 0.737150635 | -1.775314137 | 0.075846032 | 0.193649445 |
| Ccr4 | 16.30842937 | -1.370472313 | 0.784330509 | -1.74731481 | 0.080582746 | 0.203577462 |
| Bsn | 95.63553621 | -0.722828419 | 0.416945325 | -1.733628792 | 0.082983968 | 0.204483033 |
| Serping1 | 47.04288563 | -0.82936089 | 0.478629342 | -1.732783214 | 0.083134207 | 0.204483033 |
| Mme | 61.5117462 | -0.683381585 | 0.3948482 | -1.730745094 | 0.083497239 | 0.204483033 |
| Creb1 | 90.10226364 | -0.605797616 | 0.35263988 | -1.717893098 | 0.085816116 | 0.208039069 |
| Ace | 89.40349693 | -0.617756124 | 0.366971411 | -1.683390328 | 0.092299554 | 0.214657097 |
| Cxcl10 | 36.63502215 | 0.91332606 | 0.543551755 | 1.680292725 | 0.092900376 | 0.214657097 |
| Ms4a6d | 41.19158229 | 0.7918936 | 0.471363674 | 1.680005575 | 0.092956231 | 0.214657097 |
| Crhr1 | 23.24998845 | -0.907345321 | 0.540538262 | -1.67859592 | 0.093230822 | 0.214657097 |
| Igfbp4 | 146.9639961 | -0.553042104 | 0.329741818 | -1.67719735 | 0.093503897 | 0.214657097 |
| Arc | 33.58658056 | -0.807840748 | 0.48247618 | -1.674364005 | 0.094059083 | 0.214657097 |
| Plg | 15.34769777 | -1.191730705 | 0.713368239 | -1.670568775 | 0.094806884 | 0.214657097 |
| Ccl21b | 5.010252623 | -2.020616028 | 1.215062151 | -1.662973393 | 0.096317762 | 0.215072798 |
| Npy | 81.23790272 | -0.778284278 | 0.468661032 | -1.660654984 | 0.096782759 | 0.215072798 |
| Fcgrt | 134.5931683 | -0.508274257 | 0.313712191 | -1.620192877 | 0.105190851 | 0.231612883 |
| Mertk | 70.88025035 | 0.590320786 | 0.366757248 | 1.609568151 | 0.107492166 | 0.233022352 |

| | | | | | | |
|---|---|---|---|---|---|---|
| Ece2 | 268.3090353 | -0.538976698 | 0.335125177 | -1.608284709 | 0.107772838 | 0.233022352 |
| Ccl7 | 20.9250585 | -0.903099461 | 0.569720558 | -1.585162144 | 0.112929499 | 0.240888717 |
| Prph | 224.8059641 | -1.656787358 | 1.046603575 | -1.58301328 | 0.113418437 | 0.240888717 |
| Cltc | 3142.253506 | -0.468079956 | 0.297387968 | -1.573970727 | 0.115494217 | 0.24314572 |
| Ccr1 | 21.63825776 | -0.945355195 | 0.610339556 | -1.54890042 | 0.12140566 | 0.252976319 |
| Ccl3 | 27.96878825 | 0.835243457 | 0.542403109 | 1.539894301 | 0.12358612 | 0.252976319 |
| Tlr7 | 29.27726604 | 0.785125125 | 0.510882874 | 1.536800636 | 0.124342138 | 0.252976319 |
| Myc | 32.0323488 | 0.745465626 | 0.485125155 | 1.536645992 | 0.124380023 | 0.252976319 |
| Ide | 162.9568153 | -0.491165796 | 0.321731353 | -1.526633298 | 0.126852222 | 0.255836415 |
| Ubqln1 | 103.9216591 | -0.556373774 | 0.367028809 | -1.515885838 | 0.129548222 | 0.259096444 |
| Ppp2ca | 1616.822101 | -0.440564779 | 0.29689798 | -1.483892816 | 0.137837358 | 0.271782435 |
| Hck | 10.2334753 | 1.177349966 | 0.794062016 | 1.482692714 | 0.138156071 | 0.271782435 |
| Pink1 | 678.5739658 | -0.519659167 | 0.353740328 | -1.469041341 | 0.141821576 | 0.276725027 |
| Bace1 | 355.079073 | -0.466102843 | 0.322093874 | -1.447102476 | 0.147868225 | 0.284117557 |
| Zbp1 | 18.27605475 | 0.919584303 | 0.635637891 | 1.446710958 | 0.147977894 | 0.284117557 |
| Egr1 | 78.0507044 | -0.591712783 | 0.412071912 | -1.43594544 | 0.151017867 | 0.28765308 |
| Cebpd | 13.6778219 | 0.989884862 | 0.693788279 | 1.426782338 | 0.153642644 | 0.290348303 |
| Crh | 21.4966041 | -0.795087798 | 0.561632365 | -1.41567304 | 0.156871255 | 0.292422667 |
| Stat3 | 350.0977104 | 0.485710176 | 0.343348 | 1.414629402 | 0.157177183 | 0.292422667 |
| Igf1 | 54.44201694 | -0.625100272 | 0.44383854 | -1.408395658 | 0.159013944 | 0.293564205 |
| Card9 | 28.57760427 | -0.731890674 | 0.527203008 | -1.388252083 | 0.165060298 | 0.302317844 |
| ITM2b_h | 3.534688128 | -1.814426797 | 1.310743273 | -1.384273209 | 0.166274814 | 0.302317844 |
| Lrrk2 | 39.19579775 | -0.592516054 | 0.436312664 | -1.358007921 | 0.174461171 | 0.31481715 |
| Flii | 206.4162265 | -0.425732664 | 0.31472394 | -1.352717764 | 0.176145813 | 0.315485038 |
| Atp13a2 | 24.58534156 | -0.741642397 | 0.555722748 | -1.334554687 | 0.182022137 | 0.32177972 |
| S100a11 | 457.0152013 | 0.449139793 | 0.336792769 | 1.333579084 | 0.182341842 | 0.32177972 |
| Mfn1 | 150.030143 | -0.430778151 | 0.329477889 | -1.307456937 | 0.191057571 | 0.334699395 |
| Cfb | 12.21944164 | 0.9621459 | 0.740865151 | 1.298678848 | 0.194054166 | 0.337485506 |
| Ccl8 | 14.27775277 | 1.057545888 | 0.822187735 | 1.286258409 | 0.198352892 | 0.340305739 |
| Cfl1 | 1496.31922 | -0.37655061 | 0.29367364 | -1.282207727 | 0.199769786 | 0.340305739 |
| Pgk1 | 2698.011735 | -0.367280642 | 0.286545769 | -1.281752102 | 0.199929622 | 0.340305739 |
| Il1r1 | 52.22503068 | -0.521488382 | 0.413232873 | -1.261972162 | 0.206958804 | 0.349789528 |
| Map2k6 | 30.07316775 | -0.600378558 | 0.481564805 | -1.246724328 | 0.212498595 | 0.356640999 |
| Aifm1 | 136.0597781 | -0.444572851 | 0.361202599 | -1.230812991 | 0.218392816 | 0.363988026 |
| LRRK2_h | 16.78655151 | -0.78945272 | 0.644255669 | -1.225371786 | 0.220435172 | 0.364858216 |
| Mef2c | 106.9773159 | 0.396433352 | 0.332013661 | 1.194027231 | 0.232467316 | 0.38168432 |
| Entpd1 | 63.10388552 | 0.480908886 | 0.403896403 | 1.190673854 | 0.233781646 | 0.38168432 |
| APP_h | 3025.51307 | -0.343652724 | 0.292521839 | -1.174793394 | 0.240077384 | 0.389314676 |
| Cd36 | 35.09356285 | -0.60472302 | 0.528829928 | -1.143511341 | 0.252826349 | 0.4069183 |
| Cd163 | 25.72165432 | -0.584563858 | 0.512815949 | -1.139909667 | 0.254323937 | 0.4069183 |
| Cxcl9 | 23.14794283 | -0.676626426 | 0.603944479 | -1.120345411 | 0.262566598 | 0.416715003 |
| Ly96 | 32.67282591 | 0.541275046 | 0.484503358 | 1.117175014 | 0.263919502 | 0.416715003 |
| Mmp2 | 45.01547102 | -0.533251754 | 0.483803508 | -1.102207292 | 0.270371563 | 0.424112255 |
| Ackr3 | 32.55075593 | -0.519601823 | 0.482690311 | -1.076470382 | 0.28171694 | 0.439039387 |
| Tnfrsf1b | 10.04799451 | -0.919714574 | 0.862163726 | -1.066751645 | 0.286083999 | 0.442968773 |
| Igfbp2 | 321.7760219 | 0.322244182 | 0.307342279 | 1.04848634 | 0.294414592 | 0.452945527 |
| Fos | 56.2544845 | 0.427510713 | 0.416166597 | 1.027258594 | 0.304298714 | 0.465170009 |
| Tlr4 | 21.06072282 | 0.639314261 | 0.640994849 | 0.997378158 | 0.318580989 | 0.483166504 |
| Mef2b | 6.82982584 | -0.927860112 | 0.934807089 | -0.992568545 | 0.32092026 | 0.483166504 |
| Maff | 11.59065012 | 0.698116054 | 0.70507576 | 0.990129138 | 0.322111003 | 0.483166504 |
| Lrp1 | 708.7850882 | 0.360448258 | 0.366224502 | 0.984227587 | 0.325003624 | 0.484477453 |
| Grin2a | 69.12312256 | -0.482891779 | 0.497213432 | -0.971196167 | 0.331450602 | 0.49103793 |
| Casp1 | 26.8451647 | 0.543727635 | 0.575465694 | 0.944848044 | 0.34473646 | 0.50392149 |
| FTL_h | 4.888996953 | 1.04430109 | 1.116152962 | 0.935625425 | 0.349466079 | 0.50392149 |
| Il10 | 2.911398641 | -1.363044481 | 1.457388105 | -0.935265271 | 0.349651608 | 0.50392149 |
| Ms4a4a | 7.730174816 | 0.809938281 | 0.873461088 | 0.927274601 | 0.353783978 | 0.50392149 |
| SOD1_h | 5.159170042 | 0.962389631 | 1.038562488 | 0.92665549 | 0.354105434 | 0.50392149 |

| | | | | | | |
|---|---|---|---|---|---|---|
| Tnfsf9 | 3.926141761 | -1.099548973 | 1.189595995 | -0.924304535 | 0.355327782 | 0.50392149 |
| Myl2 | 6.123325679 | -0.905914388 | 0.982661032 | -0.921899168 | 0.356581173 | 0.50392149 |
| Tlr2 | 17.8898174 | 0.593355976 | 0.64410982 | 0.921203121 | 0.356944389 | 0.50392149 |
| Eif2s1 | 177.9050037 | -0.345340809 | 0.384937891 | -0.89713384 | 0.369647503 | 0.518803514 |
| MAPT_h_ex1 | 9.779331495 | -0.740763279 | 0.830146242 | -0.892328654 | 0.3722168 | 0.519372279 |
| Dock2 | 10.38878769 | 0.664424252 | 0.753950078 | 0.881257621 | 0.378178398 | 0.524640552 |
| Alox5 | 25.68591062 | 0.480004979 | 0.565151406 | 0.849338732 | 0.395692835 | 0.544216236 |
| Cat | 537.5384425 | -0.268347338 | 0.31670636 | -0.847306439 | 0.396824339 | 0.544216236 |
| Cd33 | 17.11051892 | 0.494335693 | 0.620659404 | 0.796468545 | 0.425759751 | 0.580581479 |
| Sox10 | 161.991883 | -0.241854077 | 0.309971428 | -0.780246356 | 0.435245882 | 0.590163907 |
| App | 1913.656561 | -0.234937714 | 0.310117345 | -0.757576825 | 0.448704358 | 0.60499464 |
| TARDBP_h | 2.456902106 | 1.088344926 | 1.462183071 | 0.744328769 | 0.4566776 | 0.609899046 |
| Nlrp3 | 9.76861577 | 0.588569667 | 0.794006636 | 0.741265426 | 0.458532521 | 0.609899046 |
| Ltbr | 52.89774594 | 0.289121086 | 0.3912839 | 0.738903609 | 0.45996553 | 0.609899046 |
| Mmp11 | 18.40844027 | 0.423459582 | 0.598404539 | 0.707647676 | 0.479164078 | 0.63156013 |
| Alox5ap | 23.57970374 | -0.386713429 | 0.549474053 | -0.703788336 | 0.481564599 | 0.63156013 |
| APOE_h | 4.011681369 | -0.812653394 | 1.186551002 | -0.684887032 | 0.49341521 | 0.642892096 |
| Tnfsf11 | 20.77296875 | -0.382222805 | 0.560864925 | -0.681488159 | 0.495562657 | 0.642892096 |
| P2ry12 | 152.4834625 | 0.236975096 | 0.351624399 | 0.673943834 | 0.500347022 | 0.645609061 |
| Ifih1 | 32.76349915 | 0.318234443 | 0.478324835 | 0.665310308 | 0.505852039 | 0.649221868 |
| MAPT_h_ex10 | 9.676389329 | -0.498653893 | 0.772061877 | -0.645872963 | 0.51836163 | 0.661738252 |
| Ubc | 1413.323755 | -0.192978194 | 0.302135362 | -0.638714359 | 0.52300877 | 0.662407971 |
| Il7 | 9.17580725 | -0.51393154 | 0.807347502 | -0.636567945 | 0.52440631 | 0.662407971 |
| Mafk | 23.94740586 | 0.325987564 | 0.525781823 | 0.620005389 | 0.535254239 | 0.672570771 |
| Il1rn | 5.414091671 | 0.605812413 | 1.012008079 | 0.598624087 | 0.54942359 | 0.685640151 |
| P2ry6 | 31.5023376 | 0.289077798 | 0.485353 | 0.595603196 | 0.551440341 | 0.685640151 |
| Mrc1 | 49.28592898 | -0.257669045 | 0.435664037 | -0.591439787 | 0.554225788 | 0.685640151 |
| Adam10 | 444.8717527 | -0.174722611 | 0.30168766 | -0.57915067 | 0.562487513 | 0.692292324 |
| Ptger4 | 10.4736183 | 0.430083544 | 0.758020424 | 0.56737725 | 0.570457903 | 0.698519881 |
| Axin1 | 29.06379798 | -0.271693919 | 0.495706762 | -0.548094033 | 0.583627337 | 0.711018076 |
| Treml1 | 2.847925952 | -0.711585718 | 1.342806427 | -0.529924272 | 0.596164437 | 0.721231862 |
| Ccl4 | 15.14246457 | 0.346351552 | 0.656904511 | 0.527247943 | 0.598021419 | 0.721231862 |
| Mapt_ex1 | 271.5393367 | -0.168828805 | 0.324853561 | -0.519707417 | 0.603267516 | 0.723069011 |
| Adam17 | 115.5392567 | 0.166524422 | 0.322467504 | 0.51640683 | 0.605570296 | 0.723069011 |
| Ccl11 | 12.60712687 | -0.474492505 | 0.948625737 | -0.500189365 | 0.616941746 | 0.733000094 |
| Hspb2 | 18.88749546 | -0.28578301 | 0.584162884 | -0.489218021 | 0.624687353 | 0.738546624 |
| Ece1 | 110.2331408 | -0.205287588 | 0.426784299 | -0.481010169 | 0.630509271 | 0.741775613 |
| Prkcb | 360.3996508 | -0.170300736 | 0.384908739 | -0.442444452 | 0.658167618 | 0.768678996 |
| Masp2 | 2.495662589 | -0.678319579 | 1.54088928 | -0.440213056 | 0.659782805 | 0.768678996 |
| GRN_h | 2.09631321 | 0.68109152 | 1.567483395 | 0.43451275 | 0.663916139 | 0.769757842 |
| Fcgr4 | 36.36103401 | -0.186415661 | 0.477134412 | -0.390698419 | 0.696020167 | 0.803100193 |
| Mapt_e9 | 43.48629385 | -0.164969984 | 0.432252794 | -0.381651632 | 0.702719781 | 0.806950945 |
| Tnf | 2.38494146 | 0.52968972 | 1.477878281 | 0.358412277 | 0.720034805 | 0.82289692 |
| C1ra | 40.96699992 | 0.152628295 | 0.44039589 | 0.346570661 | 0.72891389 | 0.829096368 |
| Gjc2 | 216.1963225 | -0.159674935 | 0.467361728 | -0.341651713 | 0.732613015 | 0.829373225 |
| H2Eb1 | 28.87723161 | -0.188939005 | 0.566769157 | -0.33336148 | 0.738861436 | 0.832519928 |
| Gba | 97.61836261 | 0.107160391 | 0.331259274 | 0.32349401 | 0.746321143 | 0.836053796 |
| Ccl17 | 10.70478218 | -0.279835213 | 0.874472525 | -0.32000458 | 0.748964859 | 0.836053796 |
| Mbl2 | 3.341302897 | -0.390960971 | 1.262250563 | -0.309733252 | 0.756763814 | 0.840848683 |
| Nos2 | 3.231944231 | -0.407341963 | 1.33645939 | -0.304791874 | 0.760524671 | 0.841133276 |
| Bdnf | 19.46811516 | -0.170148774 | 0.616250806 | -0.276103127 | 0.782468859 | 0.861433607 |
| Il12a | 4.488576588 | -0.320655836 | 1.222672733 | -0.262258107 | 0.793122458 | 0.869175296 |
| Plat | 185.1691614 | 0.073367614 | 0.304601874 | 0.24086396 | 0.809660555 | 0.875058529 |
| C1s1 | 16.12105577 | 0.17299739 | 0.722324189 | 0.239501034 | 0.810717095 | 0.875058529 |
| IL6ra | 36.36348287 | 0.107035252 | 0.45158955 | 0.237018885 | 0.81264214 | 0.875058529 |
| IL6 | 5.638243616 | -0.233881596 | 0.990429357 | -0.236141623 | 0.813322777 | 0.875058529 |
| Crp | 4.914457681 | -0.244642467 | 1.055567011 | -0.231764033 | 0.816721294 | 0.875058529 |

| | | | | | | |
|---|---|---|---|---|---|---|
| Cxcl15 | 4.572044739 | -0.239306312 | 1.109488856 | -0.215690595 | 0.829228933 | 0.884510862 |
| Ifng | 3.511393911 | -0.232330204 | 1.218998638 | -0.190591028 | 0.84884602 | 0.901429402 |
| Ptger1 | 4.8140964 | 0.194024498 | 1.120735581 | 0.173122458 | 0.862555173 | 0.911952606 |
| Cxcr4 | 39.44216834 | 0.067130977 | 0.491928687 | 0.136464855 | 0.891453806 | 0.9318997 |
| C5ar1 | 14.67748826 | 0.086167459 | 0.642593545 | 0.134093253 | 0.893328835 | 0.9318997 |
| Il1a | 6.86136892 | 0.138875175 | 1.073953534 | 0.129312089 | 0.897110706 | 0.9318997 |
| Tlr8 | 2.439809408 | 0.189583093 | 1.466581895 | 0.129268671 | 0.89714506 | 0.9318997 |
| Mmp3 | 3.622888436 | 0.148703361 | 1.193399316 | 0.124604866 | 0.900836376 | 0.9318997 |
| Stat6 | 59.69079943 | -0.047186435 | 0.423950066 | -0.111301869 | 0.911376974 | 0.938757398 |
| Il1b | 5.815769985 | -0.07884296 | 0.99058357 | -0.079592436 | 0.936561411 | 0.960575806 |
| Jak3 | 17.40139333 | 0.031607714 | 0.590680021 | 0.053510721 | 0.957324989 | 0.977693606 |
| H2-Ea-ps | 24.61555869 | -0.076904184 | 1.779341967 | -0.043220575 | 0.965525704 | 0.981890546 |
| IL10rb | 117.0078367 | -0.01092374 | 0.319956112 | -0.034141371 | 0.972764418 | 0.985077892 |
| Tlr6 | 12.5547107 | 0.020349133 | 0.711332757 | 0.028607052 | 0.977177988 | 0.985389567 |
| Treml2 | 6.633700269 | 0.014535531 | 0.957589287 | 0.015179296 | 0.987889139 | 0.990777724 |
| Itm2b | 3026.650505 | -0.003021764 | 0.261428465 | -0.011558666 | 0.990777724 | 0.990777724 |

**Supplementary Table 9: Overlapping gene expression changes in Il-10 expressing αSyn-seeded M83+/- mice vs control αSyn-seeded M83+/- mice**

*Base Mean=gene counts normalized to library depth; lfcSE-log (Fold Change) Standard Error; stat=Wald test statistics*

**IL-10 expressing αSyn-seeded hemizygous M83 mice**

| | baseMean | log2FoldChange | lfcSE | stat | pvalue | padj |
|---|---|---|---|---|---|---|
| Serpina3n | 1107.7152 | 2.316968978 | 0.6005363 | 3.8581662 | 0.0001142 | 0.0139634 |
| Cd68 | 181.90374 | 1.995619209 | 0.5240668 | 3.8079482 | 0.0001401 | 0.0139634 |
| Hspb1 | 806.78249 | 1.685366789 | 0.4490395 | 3.7532706 | 0.0001745 | 0.0139634 |
| Fcrls | 268.04524 | 1.834853398 | 0.5056481 | 3.6287161 | 0.0002848 | 0.0152826 |
| Timp1 | 107.14074 | 2.218824137 | 0.616364 | 3.5998603 | 0.0003184 | 0.0152826 |
| Mmp9 | 35.533296 | -1.744481496 | 0.4932617 | -3.5366244 | 0.0004053 | 0.016211 |
| Msr1 | 47.537463 | 1.977648787 | 0.5920265 | 3.3404733 | 0.0008364 | 0.0286751 |
| Il10 | 4529.896 | -6.711111992 | 2.0589761 | -3.2594415 | 0.0011163 | 0.0307374 |
| Lyz2 | 591.26697 | 1.798948023 | 0.5534639 | 3.2503442 | 0.0011527 | 0.0307374 |
| Ctsd | 10165.057 | 1.517428312 | 0.4729329 | 3.2085489 | 0.0013341 | 0.0320176 |

**Control αSyn-seeded hemizygous M83 mice**

| | baseMean | log2FoldChange | lfcSE | stat | pvalue | padj |
|---|---|---|---|---|---|---|
| Serpina3n | 786.00194 | 3.008151559 | 0.4061065 | 7.4072979 | 1.29E-13 | 5.16E-12 |
| Cd68 | 97.513795 | 2.912921634 | 0.3886413 | 7.4951424 | 6.623E-14 | 3.179E-12 |
| Hspb1 | 534.96652 | 1.769530901 | 0.4013593 | 4.4088451 | 1.04E-05 | 0.000136 |
| Fcrls | 116.98676 | 2.774988108 | 0.3887051 | 7.1390572 | 9.397E-13 | 3.222E-11 |
| Timp1 | 20.747191 | 2.122012956 | 0.5998137 | 3.5377867 | 0.0004035 | 0.0100365 |
| Mmp9 | 21.177724 | -1.583257708 | 0.6000279 | -2.6386401 | 0.0083239 | 0.035674 |
| Msr1 | 16.87142 | 1.748222853 | 0.6365469 | 2.7464165 | 0.006025 | 0.0264368 |
| Il10 | 2.9113986 | -1.363044481 | 1.4573881 | -0.9352653 | 0.3496516 | 0.5039215 |
| Lyz2 | 257.92243 | 1.342071083 | 0.3105335 | 4.3218245 | 1.547E-05 | 0.0001769 |
| Ctsd | 5819.1543 | 2.154739123 | 0.2710185 | 7.9505233 | 1.857E-15 | 1.486E-13 |

**Supplementary Table 10: List of antibodies used in the study**

| Antibody | Host/Source | Vendor | Catalog | Dilution | Antigen Retrieval |
|---|---|---|---|---|---|
| anti p62 | Rabbit | ProteinTech | 18420-1-AP | 1:2000 | Steam |
| anti GFAP | Rabbit | Cell signaling | 80788S | 1:1000 | Steam |
| anti GFAP | Mouse | Abcam | ab190288 | 1:1000 | Steam |
| anti cd11b | Rabbit | Abcam | ab133357 | 1:1000 | Citrate, pH 6.0 |
| anti cleaved caspase 3 | Rabbit | Cell signaling | 9664S | 1:250 | Citrate, pH 6.0 |
| anti MAP1LC3B | Rabbit | Cell signaling | 3868S | 1:4000 | Steam |
| anti Tyrosine hydroxylase | Rabbit | Millipore | AB152 | 1:1000 | Steam |
| anti CD68 | Mouse | Abcam | ab201340 | 1:200 | Steam |
| anti Choline acetyl transferase | Goat | Millipore | AB144P | 1:500 | Steam |
| anti Myelin Basic Protein | Rat | Abcam | ab7349 | 1:500 | Steam |
| anti pSer129 αSyn (Clone 81A) | Mouse | In house (Ref. 26) | N/A | 1:10,000 | Steam |
| anti αSyn C terminal (clone 15-4E7) | Mouse | In house (Ref. 26) | N/A | 1:10,000 | Steam |
